# Patterns of Positive Selection in Six Mammalian Genomes

**DOI:** 10.1371/journal.pgen.1000144

**Published:** 2008-08-01

**Authors:** Carolin Kosiol, Tomáš Vinař, Rute R. da Fonseca, Melissa J. Hubisz, Carlos D. Bustamante, Rasmus Nielsen, Adam Siepel

**Affiliations:** 1 Department of Biological Statistics and Computational Biology, Cornell University, Ithaca, New York, United States of America; 2 Institute of Biology, University of Copenhagen, Copenhagen, Denmark; 3 Department of Human Genetics, University of Chicago, Chicago, Illinois, United States of America; University of Aarhus, Denmark

## Abstract

Genome-wide scans for positively selected genes (PSGs) in mammals have provided insight into the dynamics of genome evolution, the genetic basis of differences between species, and the functions of individual genes. However, previous scans have been limited in power and accuracy owing to small numbers of available genomes. Here we present the most comprehensive examination of mammalian PSGs to date, using the six high-coverage genome assemblies now available for eutherian mammals. The increased phylogenetic depth of this dataset results in substantially improved statistical power, and permits several new lineage- and clade-specific tests to be applied. Of ∼16,500 human genes with high-confidence orthologs in at least two other species, 400 genes showed significant evidence of positive selection (FDR<0.05), according to a standard likelihood ratio test. An additional 144 genes showed evidence of positive selection on particular lineages or clades. As in previous studies, the identified PSGs were enriched for roles in defense/immunity, chemosensory perception, and reproduction, but enrichments were also evident for more specific functions, such as complement-mediated immunity and taste perception. Several pathways were strongly enriched for PSGs, suggesting possible co-evolution of interacting genes. A novel Bayesian analysis of the possible “selection histories” of each gene indicated that most PSGs have switched multiple times between positive selection and nonselection, suggesting that positive selection is often episodic. A detailed analysis of Affymetrix exon array data indicated that PSGs are expressed at significantly lower levels, and in a more tissue-specific manner, than non-PSGs. Genes that are specifically expressed in the spleen, testes, liver, and breast are significantly enriched for PSGs, but no evidence was found for an enrichment for PSGs among brain-specific genes. This study provides additional evidence for widespread positive selection in mammalian evolution and new genome-wide insights into the functional implications of positive selection.

## Author Summary

Populations evolve as mutations arise in individual organisms and, through hereditary transmission, gradually become “fixed” (shared by all individuals) in the population. Many mutations have essentially no effect on organismal fitness and can become fixed only by the stochastic process of neutral drift. However, some mutations produce a selective advantage that boosts their chances of reaching fixation. Genes in which new mutations tend to be beneficial, rather than neutral or deleterious, tend to evolve rapidly and are said to be under positive selection. Genes involved in immunity and defense are a well-known example; rapid evolution in these genes presumably occurs because new mutations help organisms to prevail in evolutionary “arms races” with pathogens. Many mammalian genes show evidence of positive selection, but open questions remain about the overall impact of positive selection in mammals. For example, which key differences between species can be attributed to positive selection? How have patterns of selection changed across the mammalian phylogeny? What are the effects of population size and gene expression patterns on positive selection? Here we attempt to shed light on these and other questions in a comprehensive study of ∼16,500 genes in six mammalian genomes.

## Introduction

Positive darwinian selection is an important source of evolutionary innovation and a major force behind the divergence of species. The Neutralist-Selectionist debate of the past 30 years has gradually given way to a general consensus that both neutral drift and positive selection play major roles in evolutionary change. Interest has therefore shifted to questions of which genes positive selection has affected, how strong was the effect, when did it occur, and what were its functional consequences. Heightening interest in these questions is a growing appreciation that methods for detecting positive selection can also be valuable tools for gaining insight into gene function [1]. Consequently, a wide variety of methods for detecting positively selected genes (PSGs) have been introduced, including comparative or phylogenetic methods, which make use of patterns of substitutions between species, and population genetic methods, which primarily rely on patterns of intraspecies polymorphism [2],[3]. Using these techniques, strong evidence of positive selection has been found for various genes in various organisms, including many genes involved in sensory perception, immunity, host-pathogen interactions, and reproduction (reviewed in [1],[3]).

Phylogenetic and population genetic methods for detecting positive selection serve as complementary tools for functional and evolutionary analysis. These methods operate at different time scales, with phylogenetic methods being best suited for detecting selection that operates over relatively long periods in evolutionary history, and population genetic methods being best suited for detecting more recent selection. Population genetic methods can potentially detect selection operating at individual sites, through the effects of linkage with flanking alleles, while phylogenetic methods generally require multiple sites to have been affected in a sequence of interest. At the same time, decay of linkage disequilibrium at longer evolutionary time scales can allow phylogenetic methods to more accurately pinpoint the specific locations of functionally important substitutions. In some cases, phylogenetic methods also allow such substitutions to be mapped to particular branches of a phylogenetic tree, thereby providing useful insights about the evolutionary histories of the sequences in question.

With the availability of multiple complete genome sequences, it has become possible to apply phylogenetic methods for the detection of positive selection at a genome-wide scale. Within mammals, several genome-wide scans for positive selection on protein-coding genes have been conducted, using both phylogenetic [4],[5],[6],[7],[8],[9] and population genetic [10],[11],[12],[13],[14],[15] methods (reviewed in [16]). These analyses have identified many new genes showing strong evidence of positive selection and have revealed striking differences in the prevalence of positive selection on different lineages and among different classes of genes. For example, it has been reported that PSGs are enriched for roles in sensory perception, immunity and defense, tumor suppression, apoptosis, and spermatogenesis [4],[5]; that PSGs are associated with known Mendelian disorders [4]; that PSGs often coincide with segmental duplications [8]; and that more genes have undergone positive selection in chimpanzee evolution than in human evolution [9]. Genome-wide scans for PSGs have also helped to stimulate interest in detecting positive selection on noncoding sequences and on gene expression [17],[18],[19],[20].

Nevertheless, much remains to be learned about positive selection in mammalian genomes, even within protein-coding regions. The most comprehensive scans for PSGs so far [4],[5],[7],[8],[9] have been based on at most three genome sequences—typically the highly similar human, chimpanzee, and/or rhesus macaque genomes (>97% average identity in orthologous coding regions [8]). As a result, the power for detection of PSGs has been relatively weak [5],[8]. In addition, in several of these studies, at least one genome was of draft quality, which reduced the number of genes that could be examined and required additional care in avoiding false positive predictions.

Here we present a phylogenetic analysis of positive selection in the six eutherian mammalian genomes for which high-coverage, high-quality sequence assemblies are now available: the human [21], chimpanzee [6], macaque [8], mouse [22], rat [23], and dog [24] genomes. The phylogenetic depth of this data set helps considerably in addressing the problem of weak power. Indeed, these genomes have a near-optimal degree of divergence for PSG detection, being distant enough to produce a strong phylogenetic signal, yet close enough that gene structures are well conserved, alignment is fairly straightforward, and synonymous substitutions are not saturated (e.g., [25]). In addition, our data set for the first time allows positive selection of mammalian genes to be examined genome-wide on a nontrivial phylogeny, so that insight can be gained into the particular “selection histories” of individual genes—that is, the branches of the phylogeny on which they experienced positive selection. In our analysis, we employ models of codon substitution that account for variation of selective pressure over branches on the tree and across sites in a sequence, which can capture signatures of molecular adaptation that affect small numbers of sites [26],[27]. Using a series of likelihood ratio tests (LRTs) based on these models, we identify more than four hundred genes that show strong signatures of positive selection during mammalian evolution. Our detailed analysis of the functional roles, selection histories, and expression patterns of these genes follows.

## Results

### Orthologous Genes

Using the latest human, chimpanzee, macaque, mouse, rat, and dog genome assemblies, we identified 17,489 human genes with high-confidence orthologs in at least two of the remaining five species. These *ortholog sets* (human genes and non-human orthologs) were identified by an automatic pipeline that made use of syntenic whole-genome alignments, sequence quality scores, and other data (see Methods). Briefly, the pipeline began with 21,115 human genes drawn from the RefSeq [28], UCSC Known Genes [29], and VEGA [30] gene sets. These genes were mapped to the other genomes via syntenic pairwise alignments, then passed through a series of rigorous filters to ensure correct mapping, high sequence quality, and only minimal changes between species in gene structure. This approach exploits the fact that gene structures are generally well-conserved between mammalian species [22] and avoids any dependency on the non-human gene annotations, which—with the exception of mouse—are significantly less accurate and complete than those for human. Because low-quality sequence can produce a spurious signal for positive selection (e.g., [8]), all bases with low quality scores (Phred quality <20) were masked out for subsequent analyses. Masking (or truncation at the 5′ or 3′ end) was also used to exclude regions of genes in which minor differences in gene structure were apparent. Genes that showed signs of substantial disruptions to their exon-intron structures or open reading frames in one or more species (perhaps indicating pseudogenization) were masked out completely in those species. All masked bases were treated as missing data in the subsequent analysis of positive selection. This masking approach allowed the number of genes to be maximized while ensuring that the analyzed alignments were of high quality (Table 1).

**Table 1:** Numbers of ortholog sets.

		containing	containing	containing	containing	containing
	all	chimpanzee	macaque	mouse	rat	dog
Human + ≥ 2 orthologs	17,489	15,315	14,973	14,266	12,823	13,696
Incomplete transcripts	6,113	5,317	5,219	5,037	4,562	4,938
Recent duplications	2,273*	745	816	1,476	1,319	1,089
After duplication removal	16,529	14,570	14,157	12,790	11,504	12,607

*: Recently duplicated genes are removed, but orthologs sets are retained if they still contain a human gene and ≥2 orthologs.

**Figure 1:**
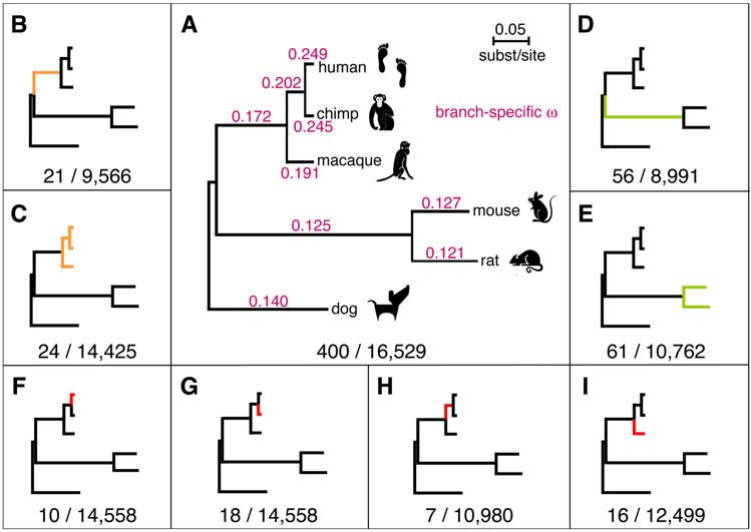
The LRTs used to detect positive selection in the six mammalian genomes. (A–I) Panel A shows the test for selection on any branch of the phylogeny, and panels B–I show the lineage- and clade-specific tests, with branches under positive selection highlighted. The numbers below each subfigure represent the number of positively selected genes identified by each LRT (FDR<0.05) and the total number of ortholog sets tested. In (A), branch lengths are drawn proportional to their estimates in substitutions per site, and each branch is labeled with the corresponding estimate of *ω*. All tests are based on an unrooted phylogeny; the trees are rooted for display purposes only. Nominal *P*-value thresholds for FDR<0.05 were: (A) 1.1×10^−3^, (B) 9.1×10^−5^, (C) 7.7×10^−5^, (D) 2.9×10^−4^, (E) 2.8×10^−4^, (F) 2.5×10^−5^, (G) 5.4×10^−5^, (H) 1.8×10^−5^, (I) 5.9×10^−5^.

For this study, we chose to avoid recently duplicated gene families and to focus on 1∶1 orthologs. This simplified the analysis, allowed for parameter sharing across genes (see Methods), and eliminated an important source of error by avoiding the need for a separate tree reconstruction for each gene family. (All ortholog sets were assumed to obey the species tree shown in Figure 1; because only an unrooted tree is needed, the topology is well accepted.) It was therefore necessary to discard any genes that showed evidence of recent duplication. This was accomplished in a pairwise fashion, by examining each human gene and orthologous non-human gene, and determining—based on BLAST matches to other genes and gene predictions in the same genome—whether either gene had a paralog that was more similar to it than the two orthologs were to each other (see Methods). Requiring that each human gene had a high-confidence 1∶1 ortholog in at least two other species reduced the total number of ortholog sets to 16,529. These sets contain a human gene and either five (42% of cases), four (28%), three (15%) or two (15%) non-human orthologs.

### Likelihood Ratio Tests for Positively Selected Genes

We performed a series of nine different LRTs to identify genes under positive selection on particular branches or clades of interest in the six-species phylogeny. In particular, we tested for selection on any branch of the tree (Figure 1A); on the branch leading to, and on any branch within, the primate clade (Figure 1B,C); on the branch leading to, and on any branch within, the rodent clade (Figure 1D,E); and on each of the four individual branches within the primate clade (Figure 1F–I). These LRTs were all based on widely used site or branch-site models of codon evolution [31],[26],[27] (see Methods). The test for all branches was applied to all 16,529 ortholog sets. For the branch- and clade-specific tests, ortholog sets were discarded if they did not contain adequate in-group or out-group data for the test in question, which somewhat reduced the number of tests (Text S1, Table S1).

The PSGs identified by each test ranged in number from only seven (the hominid branch) to 400 (the test for all branches; FDR<0.05 in all cases). As in previous studies, the numbers of genes identified by the tests for individual primate branches were small, primarily due to weak power caused by low levels of inter-species divergence. The inclusion of additional non-primate mammals does not appear to have improved the power of these tests substantially, but it does allow a distinction to be made between selection on the branches to the hominids and to macaque. The tests for selection on the branch to the primates and in the primate clade also yielded fairly small numbers of PSGs, but the tests for selection in, or on the branch to, the rodents identified somewhat (nearly three-fold) larger numbers. In general, even with the larger data set, our power to detect selection on individual lineages and clades is still fairly weak, and differences in numbers of identified PSGs almost certainly reflect differences in power more than differences in the prevalence of selection. Nevertheless, these LRTs together produced a fairly large set of high-confidence PSGs, permitting a more detailed and thorough functional analysis than has previously been possible in mammals (see below).

### Functional Analysis of Positively Selected Genes

**Figure 2:**
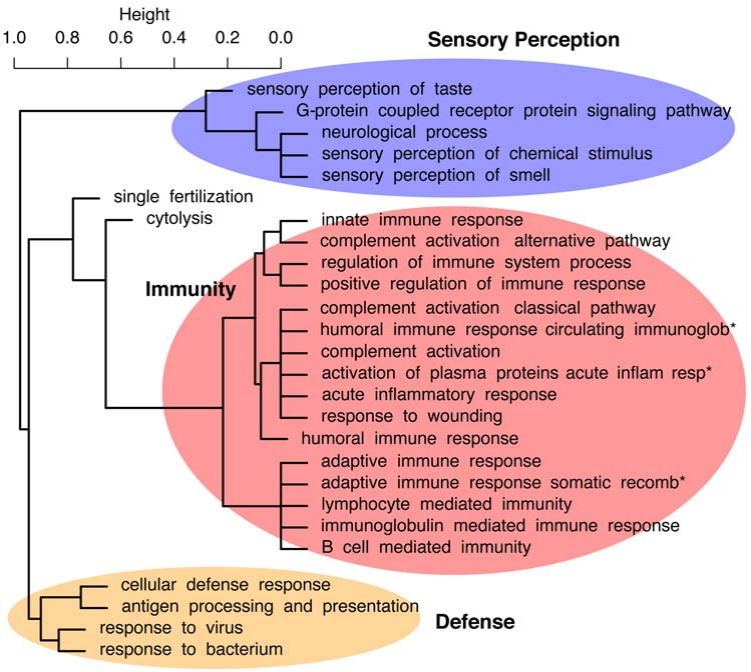
Hierarchical clustering of 27 over-represented GO categories identified by the Mann-Whitney *U* test (“biological process” group only), based on the genes assigned to each category. This dendrogram is derived from a dissimilarity matrix defined such that any two GO categories, *X* and *Y*, have dissimilarity 0 when all genes assigned to *X* are also assigned to *Y* (or vice-versa), and dissimilarity 1 when the sets of genes assigned to *X* and *Y* do not overlap. Specifically, *X* and *Y* have dissimilarity 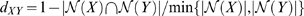, where 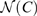 denotes the (nonempty) set of genes assigned to GO category *C*. Thus, GO categories associated with similar sets of genes group together in the dendrogram, even if these categories are not closely related in the GO hierarchy (such as “cytolysis” and “single fertilization”). Full names of abbreviated categories (*) are “humoral immune response mediated by circulating immunoglobulin,” “activation of plasma proteins during acute inflammatory response,” and “adaptive immune response based on somatic recombination of immune receptors built from immunoglobulin superfamily domains.” (Dendrogram produced by the *hclust* function in R with *method = “average”*.)

The identified PSGs are significantly enriched for a large number of functional categories, according to the Gene Ontology (GO) [32] and Protein Analysis Through Evolutionary Relationships (PANTHER) databases (Tables 2, S2, and S3). If these over-represented categories are clustered by the PSGs that are assigned to them, major groups corresponding to sensory perception, immunity, and defense emerge (Figure 2), in agreement with previous genome-wide scans [4],[5]. However, the increased power of our analysis allows biological processes and functions associated with positive selection to be identified at much finer resolution than in previous analyses, as discussed below. The increased power also seems to diminish the dependency of functional enrichments on the database or statistical methodology selected for the analysis. In particular, better agreement was observed between functional categories over-represented among the identified PSGs, as determined by Fisher's exact test (FET), and categories whose genes displayed significant shift toward smaller LRT *P*-values (whether or not they met the significance threshold for PSGs), as determined by the Mann-Whitney *U* (MWU) test (see Methods). Better agreement was also observed between analyses based on the GO and PANTHER databases (see Tables S2 and S3). The observed enrichments do not appear to be an artifact of differences between categories in gene length or alignment depth per gene (Text S1). In the discussion below, we focus on GO categories and nominal *P* -values based on the MWU test, as applied to *P*-values from the LRT for selection on any branch of the tree (except when otherwise indicated); full results are shown in Table 2 and Text S1.

**Table 2:** Selected GO categories over-represented among genes predicted to be under positive selection.

		Gene number			Fold	*P*-value	*P*-value
Category	Description	All	PSGs	E[PSGs]	Enrich.	MWU	FET
	**Biological process**						
GO:0007606	sensory perception of chemical stimulus	255	24	6.2	3.9	**4.3×10^−39^**	**1.5×10^−08^**
GO:0007608	sensory perception of smell	229	15	5.5	2.7	**6.1×10^−31^**	4.8×10^−04^
GO:0002526	acute inflammatory response	55	11	1.3	8.3	**4.7×10^−11^**	**6.7×10^−08^**
GO:0050909	sensory perception of taste	25	8	0.6	13.2	**1.4×10^−10^**	**8.3×10^−08^**
GO:0009611	response to wounding	321	23	7.8	3.0	**3.2×10^−10^**	**6.9×10^−06^**
GO:0002541	activation of plasma proteins during acute inflammatory response	28	7	0.7	10.3	**1.3×10^−09^**	**3.5×10^−06^**
GO:0006956	complement activation	28	7	0.7	10.3	**1.3×10^−09^**	**3.5×10^−06^**
GO:0045087	innate immune response	70	8	1.7	4.7	**1.9×10^−09^**	3.7×10^−04^
GO:0009615	response to virus	69	7	1.7	4.2	**3.0×10^−08^**	2.4×10^−03^
GO:0009617	response to bacterium	67	6	1.6	3.7	**4.2×10^−08^**	7.9×10^−03^
GO:0002682	regulation of immune system process	60	11	1.5	7.6	**8.5×10^−08^**	**1.0×10^−06^**
GO:0016064	immunoglobulin mediated immune response	36	8	0.9	9.2	**1.1×10^−07^**	**1.5×10^−05^**
GO:0007186	G-protein coupled receptor protein signaling pathway	792	39	19.2	2.0	**1.4×10^−07^**	2.5×10^−05^
GO:0006959	humoral immune response	56	9	1.4	6.6	**1.6×10^−07^**	**7.2×10^−06^**
GO:0050778	positive regulation of immune response	49	9	1.2	7.6	**1.7×10^−07^**	**8.4×10^−06^**
GO:0002455	humoral immune response mediated by circulating immunoglobulin	24	6	0.6	10.3	**3.0×10^−07^**	**1.8×10^−05^**
GO:0019724	B cell mediated immunity	37	8	0.9	8.9	**3.2×10^−07^**	**1.8×10^−05^**
GO:0006968	cellular defense response	55	5	1.3	3.8	**3.5×10^−07^**	1.1×10^−02^
GO:0019882	antigen processing and presentation	27	4	0.7	6.1	**5.7×10^−07^**	5.0×10^−03^
GO:0006958	complement activation, classical pathway	23	6	0.6	10.8	**6.1×10^−07^**	**1.4×10^−05^**
GO:0050877	neurological process	811	34	19.6	1.7	**7.5×10^−07^**	1.5×10^−03^
GO:0006957	complement activation, alternative pathway	11	2	0.3	7.5	**1.5×10^−06^**	2.8×10^−02^
GO:0019835	cytolysis	15	3	0.4	8.3	**2.2×10^−06^**	5.2×10^−03^
GO:0002449	lymphocyte mediated immunity	53	10	1.3	7.8	**5.7×10^−06^**	**2.5×10^−06^**
GO:0002460	adaptive immune response based on somatic recombination of immune receptors built from immunoglobulin superfamily domains	57	13	1.4	9.4	**8.0×10^−06^**	**1.1×10^−08^**
GO:0002250	adaptive immune response	58	13	1.4	9.3	**1.5×10^−05^**	**1.1×10^−08^**
GO:0007338	single fertilization	39	4	0.9	4.2	**1.7×10^−05^**	1.4×10^−02^
	**Molecular function**						
GO:0004984	olfactory receptor activity	229	15	5.5	2.7	**6.9×10^−36^**	6.8×10^−04^
GO:0004930	G-protein coupled receptor activity	625	37	15.1	2.4	**2.5×10^−14^**	**5.1×10^−07^**
GO:0004888	(*) transmembrane receptor activity	972	55	23.5	2.3	**4.0×10^−12^**	**3.3×10^−09^**
GO:0008527	taste receptor activity	14	5	0.3	14.8	**1.3×10^−08^**	**1.4×10^−05^**
GO:0008009	chemokine activity	34	5	0.8	6.1	**3.7×10^−07^**	1.3×10^−03^
GO:0004866	endopeptidase inhibitor activity	110	6	2.7	2.3	**4.1×10^−07^**	5.1×10^−02^
GO:0019965	interleukin binding	33	1	0.8	1.3	**2.2×10^−06^**	5.5×10^−01^
GO:0005125	cytokine activity	184	13	4.5	2.9	**7.8×10^−06^**	5.8×10^−04^
GO:0008173	RNA methyltransferase activity	19	1	0.5	2.2	**8.4×10^−06^**	3.7×10^−01^
GO:0004907	interleukin receptor activity	28	1	0.7	1.5	**1.1×10^−05^**	5.0×10^−01^
GO:0017171	serine hydrolase activity	150	9	3.6	2.5	**2.1×10^−05^**	1.1×10^−02^
	**Cellular component**						
GO:0005615	extracellular space	354	19	8.6	2.2	**6.8×10^−08^**	4.1×10^−03^
GO:0042611	MHC protein complex	14	4	0.3	11.8	**2.8×10^−07^**	2.8×10^−04^
GO:0016021	(*) integral to membrane	3799	168	91.9	1.8	**8.9×10^−06^**	**1.0×10^−16^**

Shown are numbers of PSGs and of all genes (out of 16,529 considered) assigned to each category or a descendant category, and one-sided (nominal) *P*-values from the Mann-Whitney *U* (MWU) and Fisher's exact (FET) tests (see Methods). Only the 400 PSGs from the test for selection on any branch of the phylogeny are considered here. Note that the MWU *P* -values do not consider whether or not each gene is predicted to be a PSG, but instead indicate the degree to which the LRT *P* -values for the genes of each category are shifted toward small values. Consequently, classes of genes experiencing relaxation of constraint but not positive selection may obtain small MWU *P* -values. In contrast, the FET *P* -values indicate over-representation of the identified PSGs within each category (or, equivalently, over-representation of each category among the PSGs). Bold indicates significance after a conservative correction for multiple testing (FWER<0.05, Holm correction). The two categories with asterisks are enriched for long genes (see Text S1). Only selected categories FWER-significant *P* -values under the MWU test are shown; see Table S2 for a complete list.

### Immunity and Defense

The PSGs are enriched for a wide variety of functions related to immunity and defense. Several over-represented categories describe activation in response to external or environmental stresses, such as from bacteria (*P* = 4.2×10^−8^), viruses (*P* = 3.0×10^−8^), wounding (*P* = 3.2×10^−8^), and acute inflammation (*P* = 4.7×10^−11^). In some cases, different categories reflect the same or very similar sets of genes (e.g., “response to wounding” and “acute inflammatory response,” or “response to virus” and “response to bacterium”), while in others they reflect quite distinct gene sets (“response to wounding” and “response to virus”) (Figure 2). Genes involved in both innate (*P* = 1.9×10^−9^) and adaptive (*P* = 1.5×10^−5^) immunity are over-represented, with many PSGs contributing to both classes. The conventional division of adaptive immunity into humoral (*P* = 1.6×10^−7^) and cellular (*P* = 3.5×10^−7^) responses is reflected in the enriched GO categories. Various mechanisms of immune response are represented, including previously identified categories for natural killer cell (*P* = 1.6×10^−8^), B-cell (*P* = 4.8×10^−7^), and T-cell (*P* = 1.2×10^−8^) mediated immunity [5],[8], and new categories such as cytokine/chemokine-mediated (7.6×10^−8^) and complement-mediated immunity (*P* = 6.0×10^−6^; see Table S3).

Some of the enriched categories point to particular pathways with large numbers of PSGs. A striking example is the complement immunity system, a biochemical cascade responsible for the elimination of pathogens. This system consists of several small proteins found in the blood that cooperate to kill target cells by disrupting their plasma membranes. Of 28 genes associated with this pathway in KEGG [33], nine are identified as PSGs (FDR<0.05), and five others have nominal *P*<0.05 (Figure S1). Most of these PSGs are inhibitors (*DAF*, *CFH*, *CFI*) and receptors (*C5AR1*, *CR2*), but some are part of the membrane attack complex (*C7*, *C9*, *C8A*), which punctures cell membranes to initiate cell lysis. Many of these PSGs are known to interact with one another, suggesting possible co-evolution. Two of three biochemical pathways known to activate the complement system are also enriched for PSGs (the classical complement pathway [*P* = 6.1×10^−7^] and the alternative complement pathway [*P* = 1.5×10^−6^]), as is the coagulation cascade that interacts with the complement system (“blood clotting,” MWU *P* = 2.2×10^−7^; Table S3). Other pathways that contain multiple interacting PSGs include those for apoptosis, taste transduction, antigen processing and presentation, and cytokine- and chemokine-mediated signaling (e.g., Figures. S4, S5).

**Figure 3:**
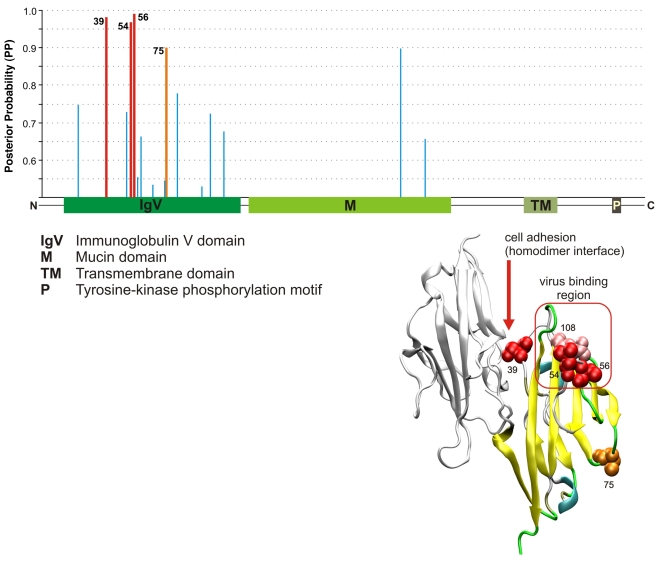
Structural analysis of the *HAVCR1* gene. At top is a graph showing the domain structure of the gene and corresponding Bayes Empirical Bayes [83] posterior probabilities (PP) of positive selection, based on our six-species alignments, with sites predicted to be under positive selection (PP>0.95) in red. At bottom right is a structural diagram (based on the structure of the *IgV* domain of the mouse gene) showing the interaction between two receptors that have been implicated in the regulation of *HAVCR1*'s immune function. It is thought that clustering of receptors within the same cell surface might facilitate phosphorylation of the cytoplasmic tail, and that interaction between receptors from different cells might be a mechanism for B–T cell adhesion [91]. Predicted residue 39 falls within the region of these receptors, very near residue 37, which directly interacts with the opposite receptor (according to the available mouse structure). In addition, predicted residues 54 and 56 are adjacent to the virus-binding surface (shown in pink), as defined by a polymorphism in macaque [91]. Interestingly, the residue that falls between them (55) appears to be critical for virus-binding at the homologous loop in the *CEA* coronavirus receptor [91]. Residue 75 in the IgV domain also shows evidence of positive selection (PP>0.90, shown in orange) but its function is unknown.

Several gene families of the immunoglobulin superfamily (“immunoglobulin mediated immune response,” *P* = 1.1×10^−7^) show particularly strong enrichments for PSGs. For example, five of the six *SIGLEC* genes included in our analysis are under positive selection (see [34]). A detailed examination of one immunoglobulin gene for which structural information was available—a cell-surface receptor for hepatitis A and other viruses called *HAVCR1* (LRT *P* = 6.9×10^−9^)—revealed several sites under positive selection in its N-terminal V-like immunoglobulin (IgV) domain. Three of these sites correspond to regions of the protein believed to play critical roles in binding to viruses or in regulating the immune function of the gene (Figure 3). In addition to its role in viral defense, *HAVCR1* is a key player in the hygiene hypothesis explaining the increase in allergies and asthma [35]. It also interacts with *IgA* (*CD79A*; *P* = 5.4×10^−9^), whose deficiency is associated with increased susceptibility to autoimmune and allergic diseases [36].

The hierarchical clustering of GO categories (Figure 2) reveals an unexpected similarity between the sets of PSGs involved in fertilization and cytolysis, and some similarity of both sets with immune-related PSGs. This association of immunity, fertilization, and cytolysis is driven by a group of genes that participate in sperm-egg interaction, but also have immune-related functions and destroy pathogens by cytolysis. Interestingly, PSGs with roles in both reproduction and immunity are often also related to cancer, and it has been hypothesized that most cancer genes under positive selection have been subject to antagonistic co-evolution, with lineage-specific variations in dynamics and strength [5],[37]. Several PSGs identified here are associated with both *FAS*/*p53* apoptosis and cancer (Da Fonseca et al., in prep.), such as the protein p53, which also regulates maternal reproduction [38]; the cell adhesion gene *ADAM2* (*P* = 2.9×10^−6^), which is integral to fertilization [39]; and the related genes *ADAM15* (*P* = 5.4×10^−4^) and *ADAM29* (*P* = 3.4×10^−4^), which are strong candidates for cancer evolution driven by sexual conflict. In addition, the testes development-related gene *CCDC54* (*P* = 3.3×10^−4^) is currently a target of cancer immunotherapy research [40].

### Sensory Perception

A smaller and somewhat less diverse group of enriched categories is associated with sensory perception. Among the most inclusive categories of this type are “sensory perception of chemical stimulus” (24 PSGs; *P* = 4.3×10^−39^) and “G-protein coupled receptor protein signaling pathway” (39 PSGs; *P* = 1.4×10^−7^). Previously, enrichments for such categories have been attributed primarily to olfactory receptors [4],[5]. Indeed, 15 PSGs are labeled as having “olfactory receptor activity” (*P* = 6.9×10^−36^). However, eight PSGs are involved in “sensory perception of taste,” including five taste receptors (*P* = 1.4×10^−10^). Interestingly, several of these are bitter taste receptors. The sense of bitter taste is critical in allowing organisms to avoid toxic and harmful substances, and extensive gene expansion of bitter taste receptors is known to have occurred during mammalian evolution [41], possibly driven by (or helping to drive) positive selection. Bitter taste receptors under positive selection include *TAS2R1*, *TAS2R5*, and a recently expanded cluster of genes at chr12p13 (*TAS2R13*, *TAS2R14*, *TAS2R42*, and *TAS2R49*). Another PSG, *TAS1R2*, is a receptor of sweet and umami taste, and the PSG *RTP3* is a transmembrane protein that is involved in the transport of taste receptors and apparently influences their expression.

The PSGs in the “neurological processes” category (*P* = 7.5×10^−7^) are dominated by olfactory and taste receptors, but they also include other types of genes. For example, *TMC2* (*P* = 1.1×10^−4^) is expressed in the inner ear and is important for balance and hearing [42]. The acid-sensing ion channel gene *ACCN4* (*P* = 1.0×10^−6^) has been implicated in synaptic transmission, pain perception, and mechanoperception [43]. *SLC6A5* (*P* = 3.0×10^−4^) is associated with hyperekplexia, a neurological disorder characterized by an excessive startle response [44]. The neuromedin receptor *NMUR* (*P* = 6.1×10^−4^) is involved in the mammalian circadian oscillator system [45],[46]. Finally, the neurotensin receptor *NTSR1* (*P* = 8.1×10^−4^) mediates hypotension, hyperglycemia, hypothermia, antinociception, and regulation of intestinal motility and secretion [47].

Similarly, the PSGs associated with diet include but are not limited to taste and olfactory receptors. For example, *MGAM* (*P* = 2.4×10^−8^) is essential for the small intestinal digestion of starch, giving it a critical role in human metabolism, as starches of plant origin make up two-thirds of most human diets [48] (see also [49]). *MAN2B1* (*P* = 1×10^−6^) is involved in the cleavage of the alpha form of mannose, a sugar monomer. Defects in this gene cause lysosomal alpha-mannosidosis, a lysosomal storage disease characterized by the accumulation of unbranched oligosaccharide chains [50]. *TCN1* (*P* = 2.9×10^−31^) is a major constituent of secondary granules in neutrophils and facilitates the transport of vitamin B12 into cells, which is important for the normal functioning of the brain and nervous system, and for the formation of blood [51]. In addition, several PSGs participate in “steroid hormone metabolism” (*P* = 8.3×10^−4^) including genes that metabolize xenobiotics and drugs (e.g., *SULT1C3*, *UGT2B7*, and *CYP2C8*). Positive selection in these and other genes is likely to have been influenced by changes in food preferences during mammalian evolution.

### Differences between Primates and Rodents

Few functional enrichments were evident for the PSGs identified by the branch- and clade-specific LRTs, primarily because these sets were quite small in size. However, the more powerful LRTs, such as those for the primate and rodent clades (Figure 1C,E), did produce significantly lower *P*-values for genes of certain functional categories than for others. Interestingly, these categories were dramatically different for the primate- and rodent-clade LRTs, with nearly all of the primate categories relating to sensory perception, and nearly all of the rodent categories relating to immunity and defense (Table 4). Indeed, the PSGs identified by the primate-clade test include several taste and olfactory receptors, as well as receptors for the sensation of pain (e.g., *MRGPRE*, *NPFF2*) and color vision (e.g., *OPN1SW*), and receptors involved in immunity (e.g., *CCR1*). The PSGs identified by the rodent-clade test include few such genes, but they include many genes involved in responses to wounding, inflammation, and stress, as well as genes involved in complement activation and innate immunity. Thus, we find little evidence that genes directly involved in brain development and function have (as a group) been driven by positive selection in primates, but many genes that provide sensory information to the brain do appear to have experienced positive selection. These changes in sensory perception could conceivably have been brought on by, or could have contributed to, increased brain size and complexity in primates.

### Bayesian Inference of Selection Histories

To gain further insight into the patterns of positive selection that have shaped present-day mammalian genes, we devised a model that allows for probabilistic inferences about the selection histories of individual genes. A selection history is defined as an assignment to each branch of the phylogeny of one of two evolutionary modes: positive selection (each site evolves with *ω*_0_<1, *ω*_0_ = 1, or *ω*_2_>1) or absence of positive selection (each site evolves with *ω*_0_<1 or *ω*_0_ = 1). The model allows a posterior distribution over selection histories to be inferred for each gene, and it allows for estimates of the number of genes under positive selection on individual branches and clades that consider uncertainty about selection histories. Unlike the branch- and clade-specific LRTs—which are simple one-sided hypothesis tests and are necessarily conservative about rejection of the null hypothesis—this model considers all candidate histories symmetrically, and allows for “soft” (probabilistic), rather than absolute, choices of history at each gene.

**Figure 4:**
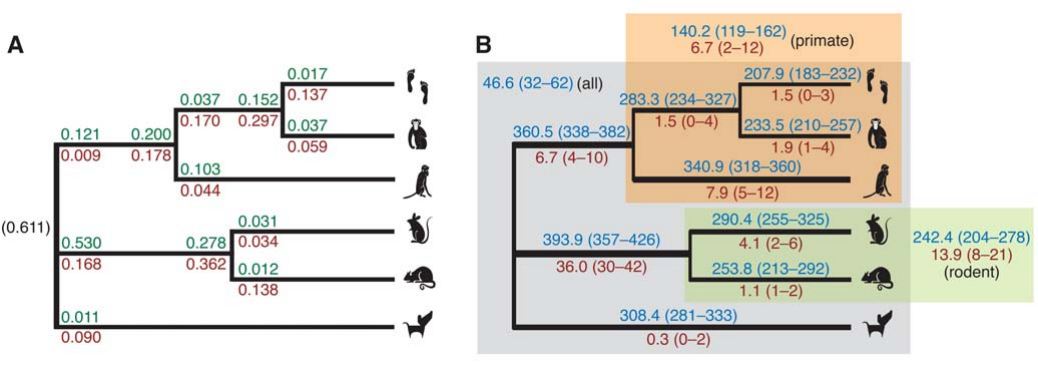
Patterns of positive selection on the mammalian phylogeny. (A) Probabilities that each gene gains (green) or loses (red) positive selection on each branch, under the Bayesian switching model. Switching events are allowed to occur early (near ancestor) or late (near descendant) on internal branches, and early on external branches. The prior probability of selection at the root of the tree is shown in parentheses. (The primate-rodent ancestor is treated as the root for this analysis; see Text S1.) The values shown are posterior means. The full posterior distributions are summarized in Figure S2. (B) Expected numbers of genes under positive selection on each branch (blue) and under positive selection only on each branch (red), out of the 544 PSGs examined, with 95% credible intervals in parentheses. Branch thicknesses are proportional to numbers in blue. Similar estimates are also shown for genes under positive selection on all branches of the primate and rodent clades (blue), on only the branches of these clades (red), and on all branches of the tree (blue). All estimates are based on 10,000 iterations of the Gibbs sampler, excluding a 100 iteration burn-in period. On each iteration, all switching parameters and the selection histories for all genes were sampled (see Text S1).

Briefly, the model is defined in terms of a simple switching process along the branches of the phylogeny. It has separate parameters for the rates of gain and loss of positive selection at several switch points on the tree, with two switch points per internal branch and one per external branch (see Figure 4A and Methods). The joint posterior distribution of these parameters and of all selection histories is inferred from the data by a Gibbs sampling algorithm (see Methods and Text S1). The inference procedure is computationally intensive, so it was applied only to the 544 genes identified by one or more LRTs as showing significant evidence of positive selection. Because in these cases the null model of no positive selection had already been rejected by a conservative test, the history without selection on any branch was excluded, leaving 2^9^−1 = 511 possible histories for the nine-branch (unrooted) phylogeny. To reduce computational cost, the inference of selection histories was conditioned on the maximum likelihood estimates of the parameters of the codon models (see Methods).

The inferred rates of gain and loss are quite variable (Figure 4A and Figure S2), with posterior means ranging from about 0.01 to 0.53. These rates are sharply reduced for the external branches of the tree, probably in large part because of diminished power to detect changes in selective mode on these branches. The number of genes inferred to be under selection also varies by branch, but not as dramatically, with expected values ranging between 207.9 and 393.9 and many 95% credible intervals overlapping (Figure 4B). Despite differences at individual branches, gains and losses appear to be roughly in equilibrium overall, with 61% of genes estimated to have been under selection at the root, and between 38% and 62% (averaging 50%) under selection at the leaves. The slight tendency to lose selection over time could reflect an ascertainment bias for genes that experienced selection early in mammalian evolution, which will tend to display signatures of selection on multiple long branches of the tree and therefore will be more easily detectable by the LRTs. The branches with the most genes under selection (such as those leading to the rodent and primate ancestors, and to dog and macaque) are generally long (see Figure 1A), suggesting power may influence these estimates. Nevertheless, the unusually high rate of gain on the branch to the rodents, and the comparatively low rate of loss on that branch (both having fairly low posterior variance; Figure S2), suggest not just differences in power but a real tendency for a net gain of selection on this branch, perhaps due to larger population sizes in the rodents. Whether because of power or a genuine increase in selection, the rodent branch appears to play a major role in the identification of PSGs. An expected 72% of the 544 candidate PSGs are under selection on this branch.

The posterior distributions over histories suggest that few genes have experienced positive selection specific to individual branches or clades (Figure 4B). Instead, most genes appear to have switched between evolutionary modes multiple times. The estimated number of mode switches per gene (averaging across genes but considering the joint posterior distribution for all selection histories) is 1.6 (95% CI: 1.5–1.7), with 0.6 gains (0.5–0.7) and 1.0 losses (0.9–1.1). An expected 91% of PSGs have experienced at least one mode switch, and an expected 53% have experienced two or more switches. 54% of PSGs have 95% CIs excluding zero switches (i.e., with high confidence, these genes have switched modes at least once), and 10% have 95% CIs also excluding one switch (with high confidence, they have switched modes at least twice). Thus, this analysis suggests that positive selection tends to be gained and lost relatively frequently in mammalian genes. Episodic positive selection has been observed and analyzed in detail at individual loci (e.g., [52],[53]) but to our knowledge genome-wide evidence of this phenomenon in mammalian phylogenies has not previously been reported. Interestingly, our observations are qualitatively compatible with Gillespie's theoretical model of an episodic molecular clock [54], although our model differs from his in detail.

By pooling information across genes and allowing for uncertainty in selection histories, this method estimates much larger numbers of genes under positive selection on each branch of the tree than do the more conservative LRTs (Figure 1). For example, the expected number of genes under selection on the branch to the primates is 360.5 (95% CI 338–382), compared with 21 genes identified by the corresponding LRT, and the expected number under selection on the branch to the rodents is 393.9 (357–426), compared with 56 identified by the corresponding LRT. In this analysis, the estimated numbers of genes that have experienced positive selection on the various primate and rodent lineages are not dramatically different, suggesting that the sharp differences from the LRTs in large part reflect inequalities in power. They also suggest that the numbers of genes under selection in recent human and chimpanzee evolution are not as different as they appear from LRTs, which will identify only the most extreme cases [9]. Indeed, the 95% CIs for the human and chimpanzee estimates heavily overlap.

### Examples of Genes with Complex Selection Histories

In addition to being useful in a bulk statistical analysis of all PSGs, the Bayesian framework can be used to identify the single most likely selection history for each gene. In some cases, these histories are consistent with known functional differences between species, and help to shed light on the evolutionary basis of these differences. For example, the sweet receptor *TAS1R2* has been shown in knock-out experiments to be responsible for differences between species in preferences for sweet tastes [55]. (Humans can taste several natural and artificial sweeteners that mice cannot, such as monellin, thaumatin, aspartame, and neohesperidin dihydrochalcone.) This gene is predicted to have experienced selection on the primate clade and on the branches leading to the primate and rodent clades (posterior probability [PP] = 0.20), suggesting that positive selection on *TAS1R2* in both primates and rodents could have contributed to differences in sweet taste preferences. Another example is the integral membrane glycoprotein *GYPC*, which plays an important role in regulating the mechanical stability of red blood cells. In humans, *GYPC* has been associated with malaria susceptibility, and predicted to have undergone recent positive selection [56]. However, we find evidence that *GYPC* has experienced positive selection on all branches of the primate clade (PP = 0.66), suggesting longer-term selective pressure that have also affected nonhuman primates. A third example is *CGA*, which encodes the alpha subunit of the four human glycoprotein hormones (chorionic gonadotropin, luteinizing hormone, follicle stimulating hormone, and thyroid stimulating hormone). This gene shows strong evidence of positive selection specific to the primate clade (PP = 0.82), consistent with the proposal that relatively recent adaptations in pregnancy and development have played a critical role in the evolution of the human endocrine system [57]. Interestingly, the closely related genes *CGB1* and *CGB2* (which encode two of the six beta subunits of chorionic gonadotropin) are thought to have originated by gene duplication in the common ancestor of humans and great apes [58], and these events could have contributed to positive selection on *CGA*. Finally, the complement components *C7* and *C8B*, which encode proteases in the membrane attack complex, are predicted with high probability to be under selection in rodents only (*C7*: PP = 0.98 for selection in mouse; *C8B*: PP = 0.93 for selection in mouse and rat). Differences in complement proteases are thought to explain certain differences in the immune responses of humans and rodents [59].

### Gene Expression

**Figure 5:**
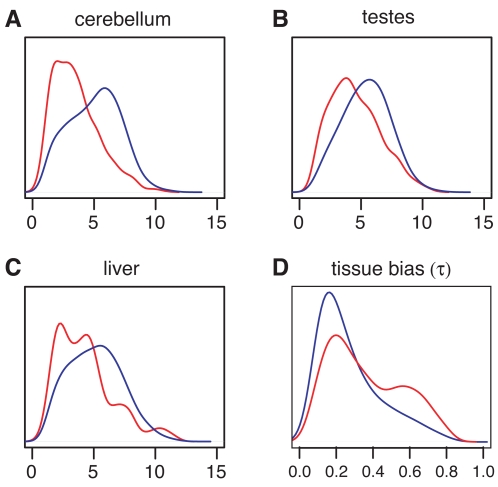
Distributions of expression levels in PSGs (red) and non-PSGs (blue) for three tissue types. (A–C) Distributions as estimated from Affymetrix Human Exon 1.0 ST Array data by the RMA algorithm [88]. The other eight tissue types showed similar differences between PSGs and non-PSGs (Figure S7). (D) Distribution of degree of tissue bias in expression levels for PSGs (red) and non-PSGs (blue), as measured by the statistic *τ* [61] (Methods). An alternative measure of tissue bias (*γ*) showed a similar pattern.

We examined the human mRNA expression levels of PSGs non-PSGs using public data from the Affymetrix Human Exon 1.0 ST Array, which contains probes for nearly all of our genes and permits accurate estimation of expression levels [60]. Our most striking finding was that PSGs show reduced expression levels in all of the 11 available tissues (breast, cerebellum, heart, kidney, liver, muscle, pancreas, prostate, spleen, testes, and thyroid; see Methods). In particular, a significantly smaller fraction of PSGs than of non-PSGs produce a hybridization signal above the background level for the array (*P*<4×10^−4^ in all tissues for PSGs defined by the all-branch test, one-sided FET). Moreover, among genes expressed above background, expression levels are significantly lower for PSGs than for non-PSGs (*P*<7×10^−5^ in all tissues, one-sided MWU test; Figures 5A–C). PSGs also show significantly greater tissue bias than non-PSGs, as measured by the statistic *τ* [61] (Figure 5D) or by an alternative statistic here denoted *γ* [17] (Methods). The differences in expression level and tissue bias between the two sets of genes do not appear to be explained by differences in false negative or false positive rates in the detection of positive selection, and the differences in expression level do not appear to be a consequence of the differences in tissue bias (Text S1). In addition, the observed differences remain if the genes that belong to strongly enriched GO categories (Table 2) are excluded, indicating they cannot be attributed to particular classes of PSGs known to have tissue-specific expression patterns, such as those involved in immunity or spermatogenesis. That expression levels are reduced in all tissues further suggests the existence of a general relationship between expression patterns and the likelihood of positive selection.

Consistent with previous observations (e.g., [62]), we found a significant negative correlation of *ω* with expression level in all 11 tissues (Spearman's rank correlation coefficient *ρ* ranged from −0.25 to −0.43). In addition, we observed a positive correlation of *ω* with tissue bias, as measured by *τ* (*ρ* = 0.24) [63],[64]. (Similar correlations were observed when the log likelihood ratio in the test for positive selection on any branch—which increases with increasing evidence for selection—was used in place of *ω*.) Unlike in previous studies, however, we were able to examine these correlations separately for positively and non-positively selected genes, using the set of PSGs identified by the all-branches LRT. Interestingly, the correlations of *ω* with expression level *τ* are much stronger within the non-PSGs than within the PSGs, indicating that the observed correlations are primarily driven by negative rather than positive selection (see also [65]). Thus, while genes expressed at low levels and/or in a tissue-specific manner show an increased tendency to have experienced positive selection, the strength of positive selection does not appear to be strongly correlated with their expression patterns (see Discussion).

Of the 15,823 genes that were tested for positive selection and had detectable expression in at least one tissue, 1,509 showed a strong preference for one tissue and were designated as tissue specific (*γ_t_*>0.25 for some tissue *t* and *γ_t_*>0.25 for all *t*′ ≠ *t*; see Methods). Based on this designation, spleen- and testes-specific genes were strongly enriched for PSGs: 22 of 174 (12.6%) spleen-specific genes were PSGs, compared with only 2.2% of other genes (*P* = 8.7×10^−11^, one-sided FET); and 45 of 715 (6.3%) testes-specific genes were PSGs, compared with 2.1% of other genes (*P* = 8.2×10^−10^). There were also significant, but weaker, enrichments for PSGs among liver-specific (*P* = 9.1×10^−3^) and breast-specific (*P* = 1.0×10^−2^) genes. Not surprisingly, the spleen-specific PSGs generally appear to be immune-related, and many of the testes-specific PSGs are involved in spermatogenesis or sperm adhesion (they include *ADAM2* and *SPAM1*; Table 3). The liver and breast specific genes are more heterogeneous. In contrast, only 2 of 254 (0.7%) cerebellum-specific genes were PSGs, compared with 2.3% of other genes (P = 0.066, one-sided FET). Only a few tissue-specific genes were identified by the clade tests, so it was not possible to compare the relationships between tissue-specific expression and positive selection in primates versus rodents. However, there were significant enrichments for primate PSGs among spleen-specific genes, and for rodent PSGs among testes-specific genes.

**Table 3:** Summary of individual PSGs discussed in this article.

**Immunity and Defense**	
Cytokine/chemokine	C-C motif: *CCL1* (*P* = 5.2×10^−4^), *CCL20* (*P* = 7.6×10^−4^);
	C-X-C and C-X3-C motifs: *CXCL5* (*P* = 8.1×10^−4^), *CX3CL1* (*P* = 3.0×10^−4^)
Complement	classical pathway: *C1S* (*P* = 2.1×10^−4^), *C4BPA* (*P* = 1.4×10^−4^),
	*C5AR1* (*P* = 6.0×10^−8^), *DAF* (*P* = 2.6×10^−6^)
	alternative pathway: *CFH* (*P* = 1.4×10^−6^), *CD46* (*P* = 2.8×10^−5^),
	*CFI* (*P* = 1.1×10^−4^), *C7* (*P* = 3.3×10^−6^), *CD59* (*P* = 8.2×10^−4^)
Immunoglobin	*SIGLEC2/CD22* (*P* = 1.1×10^−9^), *SIGLEC5* (*P* = 2.7×10^−20^), *SIGLEC6* (*P* = 2.6×10^−5^), *SIGLEC9* (*P* = 4.3×10^−5^), *SIGLEC10* (*P* = 9.5×10^−7^)
	*HAVCR1* (*P* = 6.9×10^−9^) [interacts with IgA/CD79A (*P* = 5.4×10^−9^)]
**Sensory preception**	
Taste receptors	sweet: *TAS1R2* (*P* = 1.42×10^−6^); bitter: *TAS2R1* (*P* = 1.1×10^−7^), *TAS2R5* (*P* = 1.1×10^−4^), *TAS2R13* (*P* = 5.6×10^−7^), *TAS2R14* (*P* = 2.7×10^−4^), *TAS2R42* (*P* = 2.2×10^−5^), *TAS2R49* (*P* = 1.1×10^−3^), *RTP3* (*P* = 5.2×10^−4^)
Neurological processes	neurotransmitter, neurotensin and neuromedin receptors: *SLC6A5* (*P* = 3.0×10^−4^), *NTSR1* (*P* = 8.1×10^−4^), *NMUR* (*P* = 6.1×10^−4^)
	hearing and balance: *TMC2* (*P* = 1.1×10^−4^)
**Metabolism**	
Diet	vitamin B12: *TCN1* (*P* = 2.9×10^−31^); starch digestion: *MGAM* (*P* = 2.4×10^−8^); mannose: *MAN2B1* (*P* = 1.0×10^−6^)
Steriod hormone and drug metabolism	detoxification: *SULT1C3* (*P* = 9.7×10^−13^), *UGT2B7* (*P* = 6.3×10^−10^), *CYP2C8* (*P* = 3.1×10^−4^)
**Fertility**	
Sperm-egg interaction	*ADAM2* (*P* = 2.9×10^−6^), *SPAM1* (*P* = 2.1×10^−4^), *SPACA* (*P* = 4.1×10^−5^), *WBP2NL* (*P* = 6.4×10^−4^)
Cancer related genes	*ADAM2* (*P* = 2.0×10^−6^), *ADAM15* (*P* = 5.4×10^−4^), *ADAM29* (*P* = 3.4×10^−4^), *UNQ5982/ADAM32* (*P* = 6.2×10^−7^), *CCDC54* (*P* = 3.3×10^−4^)

**Table 4:** GO categories showing an excess of small *P*-values under the primate- and rodent-clade tests.

Primate clade			
Category	Description	No. genes	MWU *P*-value
	**Biological process**		
GO:0007606	Sensory perception of chemical stimulus	195	**7.7×10^−22^**
GO:0007608	sensory perception of smell	176	**3.0×10^−18^**
GO:0007600	sensory perception	477	**9.2×10^−14^**
GO:0007186	G-protein coupled receptor protein signaling pathway	684	**5.3×10^−13^**
GO:0007166	cell surface receptor linked signal transduction	1180	**1.6×10^−10^**
GO:0050877	neurological process	711	**3.9×10^−07^**
	**Molecular function**		
GO:0004984	olfactory receptor activity	171	**6.4×10^−22^**
GO:0004930	G-protein coupled receptor activity	528	**5.1×10^−15^**
GO:0001584	rhodopsin-like receptor activity	452	**3.0×10^−12^**
GO:0004888	transmembrane receptor activity	827	**3.3×10^−11^**
GO:0004872	receptor activity	1209	**3.2×10^−09^**
GO:0004871	signal transducer activity	1560	**1.8×10^−05^**
GO:0060089	molecular transducer activity	1560	**1.8×10^−05^**
	**Cellular component**		
GO:0044425	membrane part	3691	**1.5×10^−06^**
GO:0016021	integral to membrane	3381	**1.8×10^−06^**
GO:0031224	intrinsic to membrane	3398	**2.3×10^−06^**
GO:0016020	membrane	4489	**9.6×10^−06^**

Rodent clade			
Category	Description	No. genes	MWU *P*-value
	**Biological process**		
GO:0006952	defense response	248	**5.3×10^−10^**
GO:0009611	response to wounding	226	**2.0×10^−09^**
GO:0006955	immune response	270	**3.5×10^−09^**
GO:0002376	immune system process	378	**1.3×10^−08^**
GO:0006954	inflammatory response	163	**1.4×10^−08^**
GO:0006950	response to stress	532	**3.4×10^−07^**
GO:0009605	response to external stimulus	328	**1.3×10^−06^**
GO:0002541	activation of plasma proteins during acute inflammatory response	18	**1.3×10^−06^**
GO:0006956	complement activation	18	**1.3×10^−06^**
GO:0050896	response to stimulus	1164	**2.3×10^−06^**
GO:0002455	humoral immune response mediated by circulating immunoglobulin	15	**7.1×10^−06^**
GO:0006958	complement activation, classical pathway	14	**8.7×10^−06^**
	**Cellular component**		
GO:0005576	extracellular region	601	**1.1×10^−05^**

Shown are numbers of genes classified in each category or a descendant category (out of 14,425 considered in the primate-clade LRT and 10,762 considered in the rodent-clade LRT) and *P* -values from the Mann-Whitney *U* (MWU) test. Bold indicates FWER<0.05.

Despite our large data set, we found no indication of a correlation between expression in the primate brain and recent positive selection in protein-coding regions [66] (see [67],[68]). Indeed, we found some evidence to the contrary: PSGs identified by the primate-clade test show more sharply reduced expression levels (compared with non-PSGs) in the cerebellum than in any other tissue; cerebellum-specific genes are depleted, not enriched, for PSGs; and none of the primate PSGs show tissue-specific expression in the cerebellum. These findings, of course, do not rule out positive selection in individual genes of great importance in brain development, nor do they rule out positive selection on gene expression.

### Average Rates of Protein Evolution and the Impact of Population Size

While positive selection was our primary focus, our data set also provides an opportunity to compare the average rates of protein evolution in various mammalian lineages. We estimated a separate nonsynonymous-synonymous rate ratio *ω* for each branch of the six-species phylogeny, pooling data from all ortholog sets (Figure 1A). Consistent with previous findings [6],[8], we observe that protein-coding genes, on average, have experienced moderately strong purifying selection (*ω* « 1) on all branches of the phylogeny, but that estimates of *ω* vary considerably within the mammals. These estimates are largest for the hominids (*ω*≈0.25), smallest for the non-primate mammals (0.12<*ω*≤0.14), and intermediate for non-hominid primates (0.17<*ω*<0.21). It is thought that increased estimates of *ω* in hominids primarily result from weakened purifying selection, owing to reduced effective population sizes [69],[5]. The intermediate values for non-hominid primates may also be influenced by population size.

To examine the relationship between *ω* and population size further, we made use of a theoretical relationship between *ω* and the scaled selection coefficient *γ* (see [70],[71]), which holds if nonsynonymous substitutions have equal (and small) selection coefficients, if synonymous substitutions are neutral, and if population sizes are sufficiently large (Methods). This relationship allows ratios of population sizes to be estimated from ratios of *ω* estimates, under the assumption of constant selection coefficients across species. Here we further assumed that the ancestral population sizes of humans and the chimpanzee subspecies *Pan troglodytes versus* (to which the sequenced animal belonged) were roughly the same (*N_h_* = *N_c_*) [5], and estimated the ratio of *ω_m_* in macaque to *ω_h_* in human/chimpanzee from our 10,980 human-chimpanzee-macaque ortholog trios. Our estimate of *ω_m_* / *ω_h_* = 0.732 implies an estimate for the ratio of the macaque to human ancestral population sizes of *N_m_* / *N_h_* = 1.41 [bootstrapping 95% CI (1.15, 1.64)]. In comparison, the ancestral macaque population size has been estimated at ∼73,000 [72] and ancestral human and chimpanzee population sizes at 40,000–70,000 [73],[74], which would imply a ratio of 1.04–1.82, in reasonable agreement with our estimate. We used the same theoretical relationship to devise a LRT indicating whether or not each gene deviated significantly from the assumed model with *N_m_* / *N_h_* = 1.41 (Methods). For the vast majority (96%) of the 10,980 genes examined, no significant deviation was observed, indicating that the differences in selection pressure in macaque and the hominids are generally well-explained by differences in population size.

### Power Comparison

**Figure 6:**
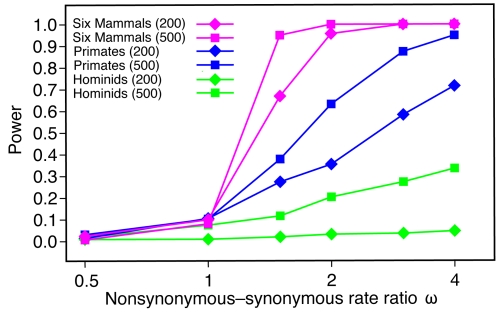
Power of the LRT for selection on any branch of the phylogeny as a function of the nonsynonymous-synonymous rate ratio *ω*. Power is defined as the fraction of tests resulting in nominal *P*<0.05. (The effect of controlling for multiple comparisons is shown in Figure S3.) When *ω*≤1, these fractions are estimates of the false positive rate. Each data point is based on 1000 data sets simulated with *evolver* [84] under the assumption of a constant *ω* among lineages and among sites (model M0). All other parameters (including the transition-transversion ratio *κ*, the codon frequencies, and the branch lengths) were fixed at values estimated from the real data. Results are shown for short (200-codon) and long (500-codon) genes and three sets of species: hominids (human and chimpanzee), primates (human, chimpanzee, and macaque), and all six mammals. Details on the computation of *P*-values are given in Text S1. Note the logarithmic scale on the *x*-axis.

To compare the power of our LRTs with the power of previous tests based on hominid or primate genomes, we simulated data sets under a range of parameter values and measured the fraction of cases in which positive selection was predicted (Figure 6). These experiments show that power increases substantially when the set of species under consideration is expanded from the two hominid species to the three primates then to all six mammals. With hominid species only, power is poor even when selection is quite strong (e.g., ∼20% with a constant *ω* = 2 and ∼40% with *ω* = 4), suggesting that a genome-wide scan will tend to identify only the most extreme cases of positive selection. If a rigorous correction for multiple testing is applied, a test based on hominids only has essentially no power, even for fairly long genes under strong selection (Figure S3; see also [5]). The situation is considerably improved by the addition of the macaque genome, but power remains poor when controlling for multiple testing unless genes are long and selection is strong. When all six mammals are considered, however, power increases substantially. With the full data set, power is reasonably good (≥70%) even when genes are short and selection is moderate in strength; it remains good when multiple comparisons are considered (Figure S3). The absolute estimates of power from these experiments depend on the simplifying assumptions used in the simulations (including the unrealistic assumption of constant *ω* among lineages and among sites), and they must be interpreted cautiously. However, estimates of relative power—which will be less sensitive to these simplifying assumptions—indicate a substantial improvement is achieved by the addition of the three non-primate mammals.

## Discussion

Since it first became possible to compare the sequences of complete mammalian genomes about five years ago, a number of genome-wide scans for positively selected genes (PSGs) have been conducted using phylogenetic methods [4],[5],[6],[7],[8],[9]. These studies have provided a valuable initial assessement of the genome-wide landscape of positive selection in mammals, but they have left many important questions unanswered. The analysis presented here, by incorporating non-primate mammalian genomes into a genome-wide scan for positive selection, represents a significant step forward. The larger, more divergent group of species improves power significantly, and the use of a nontrivial phylogeny provides insight into the particular patterns of positive selection that have helped to shape present-day genes. To our knowledge this is the largest and most detailed genome-wide analysis of positive selection to date, not only in mammals but in any group of organisms (although extensive analyses, similar in some respects, have been performed recently in *Drosophila* [75],[76]).

One finding of particular interest was that several whole pathways are especially rich in PSGs. Examples include the classical and alternative pathways for complement-mediated immunity and the *FAS*/*p53* apoptotic pathway (Figures S1, S4 and S5). These findings suggest that positive selection may frequently act directly on whole protein complexes or pathways (see [77],[78]). Alternatively, adaptive changes in one protein may sometimes have a cascade effect, leading to changes in other genes that bring a system back into equilibrium. Whether or not all changes affecting a pathway are driven by positive selection, one might expect to see similarities in the selection histories of gene with closely related functions. Indeed, we have found that genes with similar selection histories on average have substantially greater similarity in their GO categories than do genes with more divergent histories (Figure S6). The observations that multiple interacting genes often show evidence of positive selection and that positive selection is frequently episodic may well be connected. For example, in some cases a transient external force could induce a burst of changes in multiple genes that participate in the same pathway, either separately or by triggering a cascade of interdependent events. Further unraveling the (co-)evolutionary histories of interacting PSGs promises to be a fertile area for future work. Care will be required to distinguish between true co-evolution and correlations that can be explained by dependencies on expression levels or other covariates of evolutionary rate [79].

Our finding that PSGs are expressed at lower levels and in a more tissue-specific manner than non-PSGs is consistent with a well-known negative correlation *ω* with expression level, and a positive correlation of *ω* with tissue bias (*τ* or *γ*). Various explanations have been proposed for the observed decrease in *ω* among genes expressed at high levels and/or expressed broadly across tissues, including selection for translational efficiency, selection against misfolding, or increased selection due to pleiotropy [62],[68],[65]. In any case, these genes do appear to experience a reduction in their evolutionary “flexibility” compared with genes expressed at low levels and/or nonuniformly across tissues. Our observation of decreased rates of positive selection among these genes—and increased rates among low-expression/high-tissue-bias genes—is consistent with this characterization. Interestingly, however, we observe that correlations of *ω* with expression level and *τ* hold strongly within non-PSGs, but are much less pronounced within PSGs. Thus, expression levels and patterns are strongly correlated with both the strength of negative selection and the likelihood of positive selection, but they are only weakly correlated with the strength of positive selection. It appears that genes may be more likely to come under positive selection if they are in a state of evolutionary flexibility brought on by reduced or tissue-specific expression, but once positive selection has taken hold their subsequent evolutionary course is not strongly dependent on their expression patterns.

As additional mammalian genomes become available, the statistical power to detect positive selection will improve. However, most forthcoming genomes are being sequenced at low coverage, and will inevitably exhibit increased levels of error in base calls, genome assemblies, ortholog identification (due to short contigs), and alignment—all of which can lead to spurious signals for positive selection. (The same errors tend to produce false negatives, rather than false positives, in the identification of conserved elements.) Thus, careful data quality controls will be needed to take advantage of these data. In addition, when considering the impact of additional sequences on statistical power, it is useful to distinguish between positive selection that acts continuously (or in recurrent episodes) over a long evolutionary period, and positive selection that acts transiently or in a lineage-specific manner. Deep phylogenetic sequencing should generally improve detection power for continuous or recurrent positive selection, but power for transient selection depends strongly on the sequenced species and the lineages of interest. For example, the genome sequences of a dozen non-primate mammals will likely have little effect on the power to detect human-specific selection, while the gorilla and neanderthal genomes could help considerably. There are fundamental limitations in the detection of weak, transient, or highly localized positive selection that will not be overcome by any amount of genome sequencing. Nevertheless, the availability of several new primate genomes, including those of the orangutan, marmoset, and gorilla, may significantly improve power for PSGs in primates.

Our ability to connect positive selection with function remains rudimentary, but gradual progress is being made. As additional sequence data becomes available, it will become possible to associate selection with individual residues of proteins with greater accuracy. At the same time, more data is becoming available on the specific functional roles of individual amino acids, for example, from structural or mutagenesis studies. As a result, it will increasingly become possible to find direct links between selection and function. Often these links will initially be tentative, as in our site-specific analysis of the *HAVCR1* gene. Nevertheless, they provide a valuable starting point for experimental follow-up. At the same time, more can be done to incorporate non-sequence data—such as structural and expression data—into computational methods for detecting positive selection. Thus, improvements in both computational and experimental methods will be needed to establish deeper and more informative connections between evolutionary dynamics and molecular function.

## Methods

### Ortholog Identification

The latest human (hg18), chimpanzee (panTro2), rhesus macaque (rheMac2), mouse (mm8), rat (rn4), and dog (canFam2) genome assemblies were obtained from the University of California, Santa Cruz (UCSC) Genome Browser. Human-referenced whole-genome alignments were constructed from syntenic pairwise alignments with human (the “syntenic nets”) using the UCSC/MULTIZ alignment pipeline [80],[81]. Low quality bases (Phred score <20) from the chimpanzee, macaque, rat, and dog genomes were converted to ‘N’s in these alignments.

A starting gene set was composed from of the human RefSeq [28], UCSC Known Genes [29], and VEGA [30] annotations (downloaded from UCSC Feb. 19, 2007). Transcripts that lacked annotated coding regions (CDSs), that had CDSs of <100 bp, or that had CDSs whose lengths were not multiples of three were discarded, leaving 88,879 nonredundant transcripts. These transcripts were grouped by same-stranded CDS overlap into 21,115 genes (transcript clusters). All transcripts were mapped from human to each of the other five mammalian species via the syntenic alignments, then subjected to a series of filters designed to minimize the impact of annotation errors, sequence quality, and changes in gene structure on subsequent analyses. Briefly, each human transcript was required (1) to map to the non-human genome via a single chain of sequence alignments including ≥80% of its CDS; (2) after mapping to a non-human species, to have ≤10% of its CDS in sequencing gaps or low quality sequence; (3) to have no frame-shift indels, unless they were compensated for within 15 bases; (4) to have no in-frame stop codons and to have all splice sites conserved. To allow for genes that are mostly conserved but whose start or stop codons have shifted, incomplete transcripts—with ∼10% of bases removed from the 5′ and 3′ ends of the CDS—were also considered. The final collection of ortholog sets was obtained by selecting, for each gene, the (complete or incomplete) transcript that successfully mapped to the largest number of non-human species. In the case of a tie, the transcript with the greatest total CDS length was selected. This procedure resulted in 17,489 genes with ≥2 non-human orthologs, averaging ∼5 species per gene (including human; see Table 1).

To establish 1∶1 orthology, each human gene and putative non-human ortholog was examined for evidence of an inparalog (a paralog arising from a recent duplication [82]) with respect to the other species. Specifically, if either gene had a BLASTN match within the same species (with ≥80% CDS alignment) that was more similar than the two orthologs were to each other, then that gene was considered recently duplicated and was excluded from the analyses of positive selection. The removal of a duplicated gene did not require an ortholog set to be discarded entirely, provided a human gene and ≥2 nonhuman orthologs still remained. A collection of genes and gene predictions from the UCSC Genome Browser were used in the identification of inparalogs. When comparing rodent vs. non-rodent and rodent vs. rodent distances, a simple correction for unequal rates of evolution was applied. Further details are given in Text S1.

### Likelihood Ratio Tests

The LRT for selection on any branch of the phylogeny is essentially Nielsen and Yang's [31] test of site models 2a versus 1a, and the lineage- and clade-specific LRTs are essentially instances of Yang and Nielsen's [26] test 2 (see also [83],[27]). However, to reduce the number of parameters estimated per gene, the complete set of 17,489 genes was divided into eight equally sized classes by G+C content in third codon positions. The branch lengths and the transition-transversion rate ratio *κ* were estimated for each class under the null model, and these estimates were subsequently held fixed, in a G+C dependent way, for the LRTs. Instead of a complete set of branch lengths, a single scale parameter *μ* was estimated per gene. Thus, only the parameters *μ*, *ω*_0_<1 and *p*_0_ for the null model and the additional parameters *ω*_2_>1 and *p*_1_ for the alternative model, were estimated per gene (see [31],[26]). This parameterization speeds up calculations substantially compared to estimating *κ* and a set of branch length per gene, while its sensitivity, specificity and power to detect positive selection are comparable (Text S1). We developed our own software for likelihood computation and parameter estimation to support this parameterization.

For the LRT for selection on any branch, *P*-values were computed empirically, based on simulation experiments. 10,000 alignments were simulated under the ‘nearly neutral model’ (allowing for a fraction *p*_0_ of sites to evolve with *ω*_0_<1 and a fraction 1−*p*_0_ to evolve with *ω*_1_ = 1) for each G+C class using *evolver* [84]. Alignment lengths and values of *μ*, *ω*_0_ and *p*_0_ were drawn from the empirical distribution defined by the real alignments (using estimates obtained under the null model), and the remaining parameters were fixed at global estimates for each G+C class. Log likelihood ratios (LLRs) were then computed exactly as for the real data. The nominal *P* -value for a LLR of *r* was defined as the fraction of all simulated alignments with LLR≥*r*, unless the number of such alignments was <10, in which case we assumed 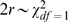 (an adequate approximation for small *P*-values, according to the simulation experiments). The method of Benjamini and Hochberg [85] was used to estimate the appropriate *P*-value threshold for a false discovery rate of <0.05. For the lineage- and clade-specific LRTs, *P*-values were computed assuming the null distribution was a 50∶50 mixture of a 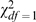 distribution and a point mass at zero (see [27] and discussion in Text S1).

### Bayesian Inference of Selection Histories

Let X = (*X*_1_,…, *X_N_*) be the alignment data, with *X_i_* denoting the alignment for the *i* th gene (1≤*i*≤*N*; here *N* = 544), and let Z = (*Z*_1_,…, *Z_N_*)be the set of selection histories, with *Z_i_* denoting the selection history for the *i* th gene (1≤*Z_i_*≤*M*; here *M* = 511). Recall that a selection history is defined as a pattern of presence or absence of positive selection on the branches of the unrooted phylogeny. Let *Z_ib_* ∈ {0,1} indicate the selective mode (with 1 representing positive selection) for branch *b* ∈ {1,…,*B*} (here *B* = 9) under history *Z_i_*. The parameters of the switching model, denoted *θ*, are defined below. The model assumes independence of genes and independence of histories, and conditional independence of X and *θ* given Z. Thus, the complete data likelihood is given by:
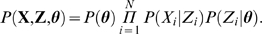
(1)


The probability of a history, *P*(*Z_i_*|***θ***), is a function of the set of switches in selective mode required to explain the history parsimoniously. For each history to be explained parsimoniously, switches must be allowed to occur *early* (near the ancestor) or *late* (near the descendant) on each internal branch, as well as (early) on each external branch (Figure 4A; see Text S1 for a justification of the model). Thus, there are twelve possible switch points, with three of them adjoining each of the four internal nodes of the tree. It is convenient to denote these points 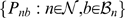 where 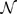 is the set of internal nodes and 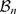 represents the branches adjoining node *n*. Let *V_nb_* ∈ {0,1} and *W_nb_* ∈ {0,1} indicate the selective states before and after point *P_nb_*, respectively. For a given history *Z_i_*, these variables are uniquely determined by parsimony according to a simple algorithm (see Text S1). The four possible values of (*V_nb_*, *W_nb_*) correspond to four possible scenarios at *P_nb_*—gain of selection (0,1), loss of selection (1,0), absence of gain (0,0), or absence of loss (1,1). The probabilities of these scenarios (i.e., the conditional probability of each *W_nb_* given *V_nb_*) are defined by a parameter for gains (*θ_nbG_*) and a parameter for losses (*θ_nbL_*) at each point. In addition, the prior probability of selection at the root of the tree is given by a parameter *θ*_0_. (For this analysis, the most recent common ancestor of the primates and rodents is treated as the root of the tree; see Text S1.) The set of parameters can thus be described as 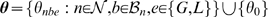. The prior probability of a history *Z_i_* is simply a product of the prior and the relevant switching probabilities:
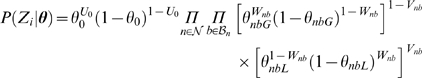
(2)where *U*_0_ represents the selective state at the root.

The switching model effectively defines a prior distribution over histories, which tends to favor simpler histories over more complex ones (typically *θ_nbe_*<0.5). The prior probability for each element of *θ* is defined by a (conjugate) Beta distribution with parameters *α* and *β* (here, *α* = 1, *β* = 9). Because these elements are independent in the prior,
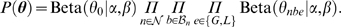
(3)


The term *P* (*X_i_* | *Z_i_*) in equation 1 is simply the likelihood at gene *i* of a branch-site codon model that assumes selection history *Z_i_*. A full Bayesian approach would integrate over the parameters of these codon models, but this would be computationally prohibitive. Instead, we make the Empirical Bayes simplification of conditioning the analysis on maximum likelihood estimates of the parameters of the codon models. The maximized log likelihoods *L_ij_* for all genes *i* and histories *j* are precomputed using existing software (in parallel, on a large computer cluster) and stored in an *N*×*M* matrix, which is then used in the inference of selection histories.

The variables Z and *θ* are unobserved, and the goal is to infer their joint posterior distribution,
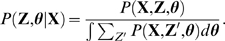
(4)


This inference was accomplished by a Gibbs sampling algorithm that alternates between sampling each *Z_i_* conditional on *X_i_* and a previously sampled *θ*, and sampling each element of *θ* conditional on a previously sampled Z. It is straightforward to derive the required conditional distributions and to sample from them (Text S1). The Gibbs sampler converges rapidly and mixes well. Notice that, because the history without selection on any branch is excluded, all of the histories are described by codon models with the same number of parameters. Therefore, no penalty for parameter number is needed when comparing histories.

After an appropriate burn-in period, each iteration of the Gibbs sampler produces a sample (Z^(*t*)^, *θ* ^(*t*)^) from *P*(Z, *θ*|X). Estimated posterior expected values of interest were obtained by averaging these samples or functions of these samples, and Bayesian 95% confidence intervals were obtained by taking the 0.025 and 0.975 quantiles of the sampled values. For example, the posterior expected number of genes under selection on branch *k* (see Figure 4) was estimated as 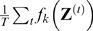, where *T* is the number of samples and the function *f_k_*(Z) counts the number of genes under selection on branch *k* in a set of histories Z.

### Analysis of Over-Represented Functional Categories

Each gene was assigned categories from the GO [32] and PANTHER [86] databases (downloaded on June 26, 2007), based on the Uniprot identifiers of associated transcripts. At least one GO category was identified for 14,137 (86%) genes, and at least one PANTHER category for 13,753 (83%) genes. To account for the hierarchical nature of these databases, each gene was also considered to belong to all parent categories of the ones to which it was directly assigned. For each category *C* and set of PSGs *S*, a 2×2 contingency table was constructed for the numbers of genes assigned or not assigned to *C*, and within and outside*S*, then a (one-sided) *P*-value for independence of rows and columns was computed by Fisher's exact test. In addition, the distributions of LRT *P* -values among the genes assigned to *C* and not assigned to *C* were compared by a (one-sided) Mann-Whitney *U* (MWU) test. (Notice that *S* is not considered in this case.) Nominal *P* -values computed by the FET and MWU tests were corrected for multiple comparisons using the method of Holm [87].

### Gene Expression

The analysis of gene expression was based on the publicly available “Tissues+Mixtures” sample data set for the Affymetrix GeneChip Human Exon 1.0 ST Array (http://www.affymetrix.com/support/technical/sample_data/exon_array_data.affx). The RMA-based probeset summaries [88] and DABG (detected above background) -values were used. Each probeset was assigned genomic coordinates using the “Affy All Exon” track in the UCSC browser (hg17), then was associated with any human gene from our set having an exon on the same strand that completely contained the probeset. Nearly every gene (98%) had at least one probeset.

To calculate a *P*-value for each gene×tissue, the DABG *P*-values of all associated probesets (pooling the three replicates per probeset×tissue) were combined using Fisher's method [89]. A gene was considered to be significantly expressed above background if it had (nominal) *P*<0.001. Similarly, an estimated expression intensity for each gene×tissue was calculated by first taking the median over the three replicates of each RMA-based probeset summary, then taking the median of these values over all probesets associated with the gene. The analysis of expression intensities was restricted to genes significantly expressed above background so that genes expressed at or near the background level did not drive the results.

To measure tissue bias, we used: (1) the statistic *τ* [61], which represents the average difference in normalized expression intensity from that of the tissue of maximal expression, and (2) a statistic, here denoted *γ*, defined as *γ* = max*_t_γ_t_*, where *γ_t_* is the squared cosine of the angle between the expression vector and the coordinate axis associated with *t* (see [17]). In defining genes as tissue specific for tissue *t* we required *γ_t_*>0.25 and *γ_t′_*<0.125 for all *t′* ≠ *t*. Further details are given in Text S1.

### Analysis of Average Rates of Protein Evolution and Population Size

Maximum likelihood estimates of *ω* for each branch were obtained using the codeml program in the PAML software package [84], with F3×4 codon frequencies, estimation of *κ* (fix_kappa = 0) and a single *ω* across sites per branch (model = 1, NSsites = 0). The tree topology shown in Figure 1 was assumed. The alignments for all genes were concatenated for this analysis.

Assuming all non-synonymous mutations at a given gene have the same selection coefficient and all synonymous mutations are neutral, population genetic theory says that *ω* should be given by [70],[90]:
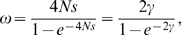
(5)where *γ* = 2*Ns*. Therefore, *γ* can be estimated as *f* ^−1^(*ω*), where *ω* = *f*(*γ*) denotes the function above. (Values of *γ* can be obtained numerically; see Text S1.) Ratios of population sizes can therefore be estimated from ratios of *ω* estimates: 
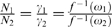
.

The LRT to test whether differences in population size can explain the differences in *ω* in human and macaque was constructed as follows. The null model assumes *ω_h_* = *ω_c_* and *ω_m_* = 0.732*ω_h_* (see Results). The alternative model also assumes *ω_h_* = *ω_c_* but leaves *ω_m_* as a free parameter to be estimated from the data. Because the models are nested, a 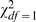 distribution is used for significance testing. This test was applied separately to each gene.

### Website and Browser Track

A website is available at http://compgen.bscb.cornell.edu/projects/mammal-psg/ with definitions of the candidate genes (accession numbers, genomic coordinates, and descriptions), multiple alignments of orthologous gene sets, GO and PANTHER category assignments, detailed results of the LRTs and the Bayesian analysis, and other resources. In addition, the candidate genes and predicted PSGs are displayed as a track in the UCSC Genome Browser (http://genome.ucsc.edu; assembly hg18).

## Supporting Information

Figure S1 Complement component and coagulation pathways.
(0.70 MB TIF)

Figure S2 Boxplot of marginal posterior distributions.
(0.17 MB TIF)

Figure S3 Full power results.
(0.34 MB TIF)

Figure S4 Apoptosis pathway.
(0.47 MB TIF)

Figure S5 p53 signaling pathway.
(0.45 MB TIF)

Figure S6 GO similarity.
(0.29 MB TIF)

Figure S7 Expression results for all tissues.
(0.50 MB TIF)

Table S1 Minimum species configurations required for likelihood ratio tests.
(0.02 MB PDF)

Table S2 GO categories over-represented among predicted PSGs.
(0.11 MB PDF)

Table S3 PANTHER categories over-represented among predicted PSGs.
(0.10 MB PDF)

Text S1 Supplementary methods with complete set of supplementary figures and tables (including some not referenced in main article).
(2.86 MB PDF)
