# Supplementary figures and images for: Patterns of Positive Selection in Six Mammalian Genomes
Source: PLoS Genet. 2008 Aug 1;4(8):e1000144. doi: 10.1371/journal.pgen.1000144 (PMC2483296; doi:10.1371/journal.pgen.1000144)

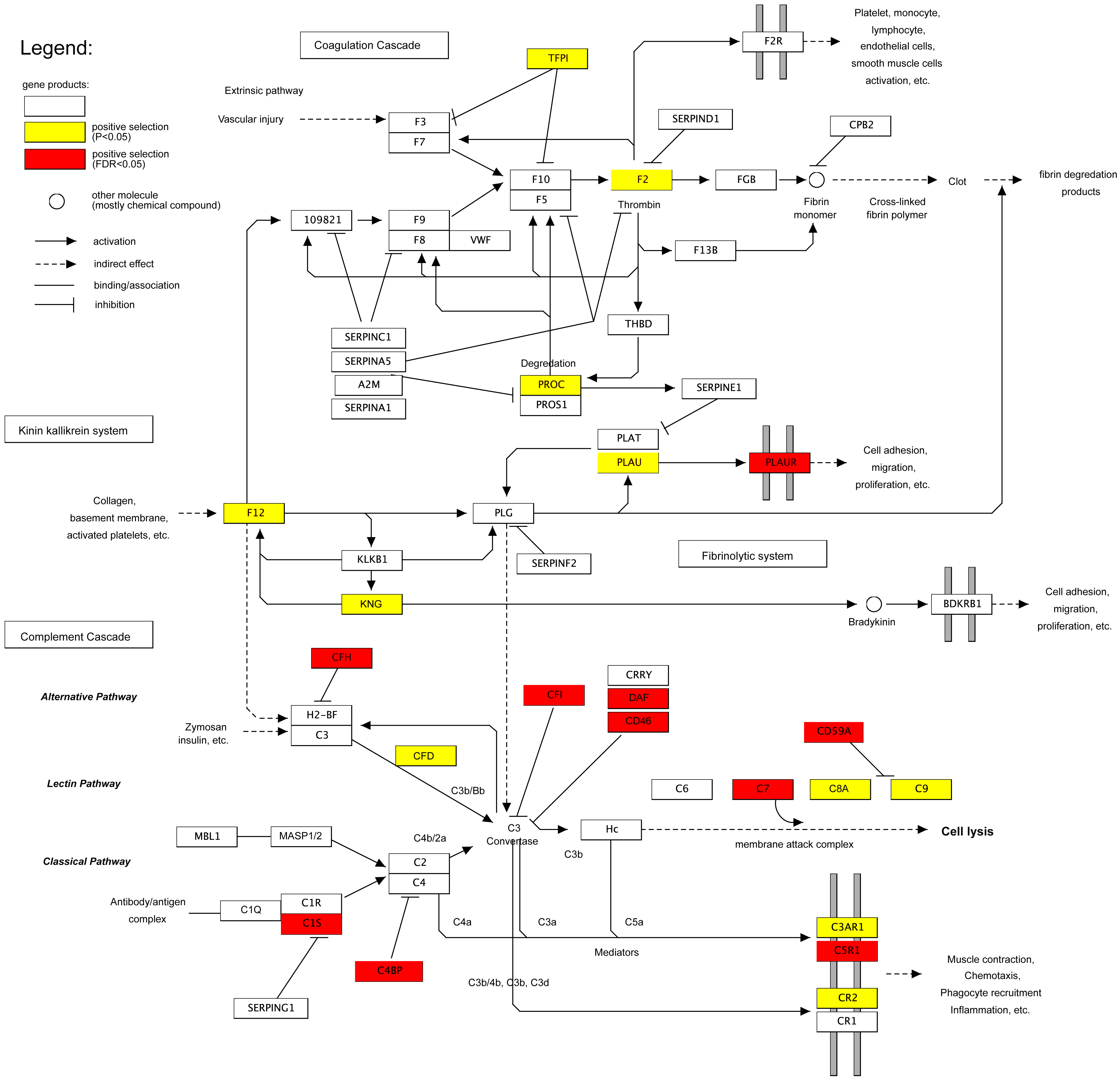

Supplement: Figure S1 — Complement component and coagulation pathways. (0.70 MB TIF) [file pgen.1000144.s001.tif]

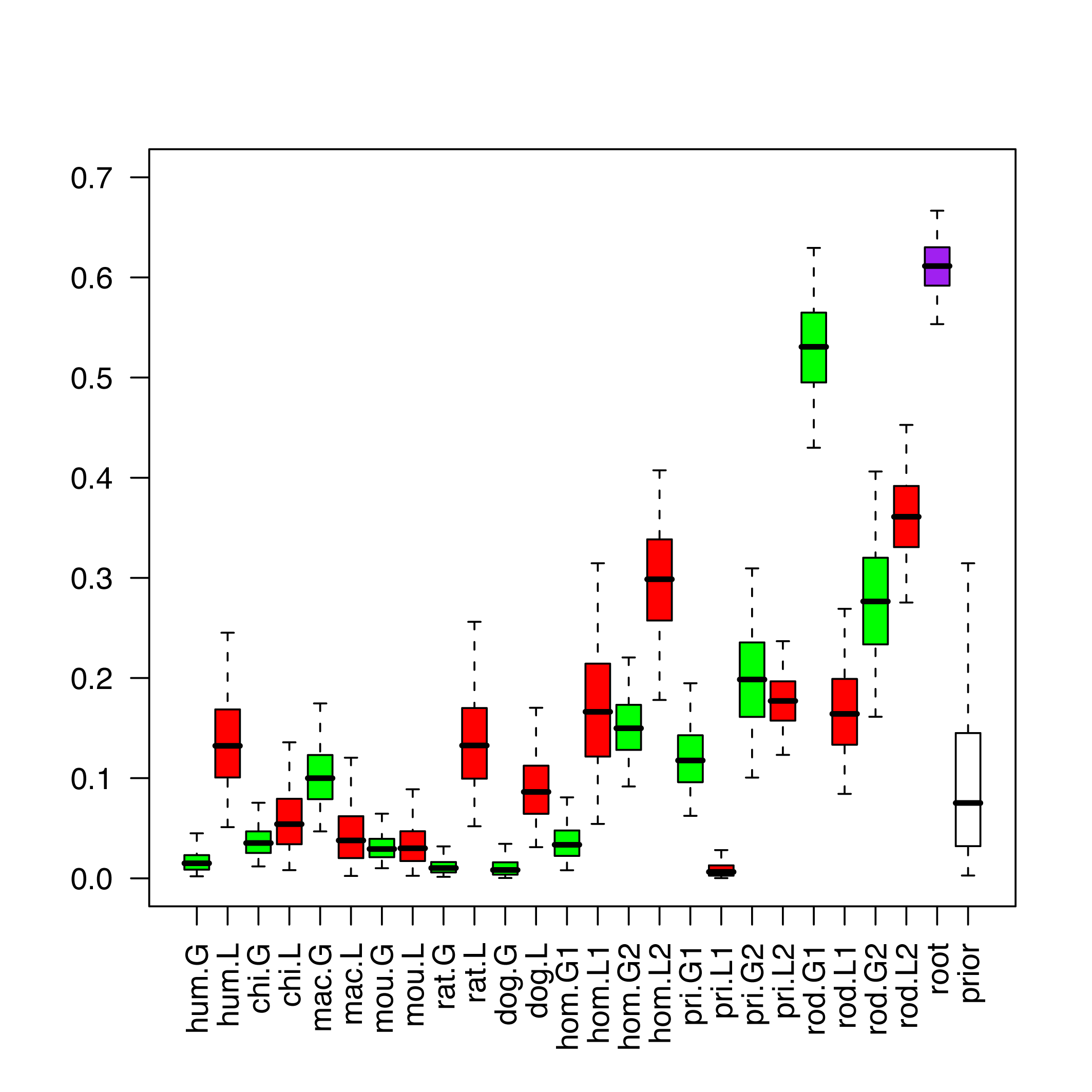

Supplement: Figure S2 — Boxplot of marginal posterior distributions. (0.17 MB TIF) [file pgen.1000144.s002.tif]

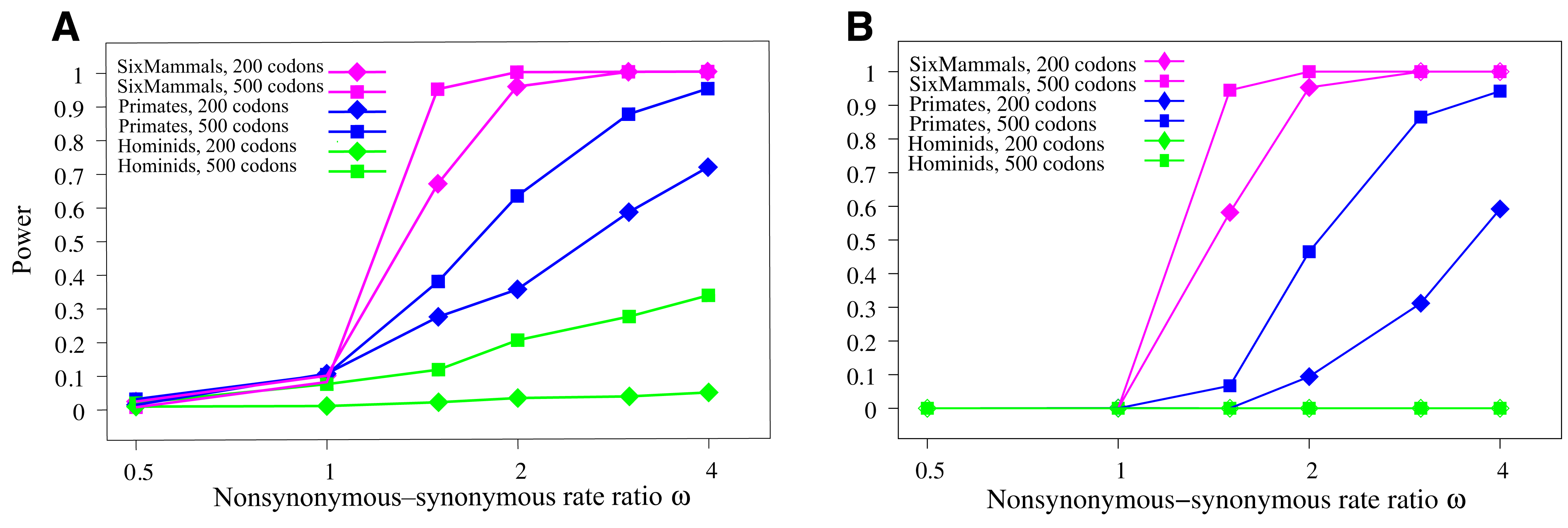

Supplement: Figure S3 — Full power results. (0.34 MB TIF) [file pgen.1000144.s003.tif]

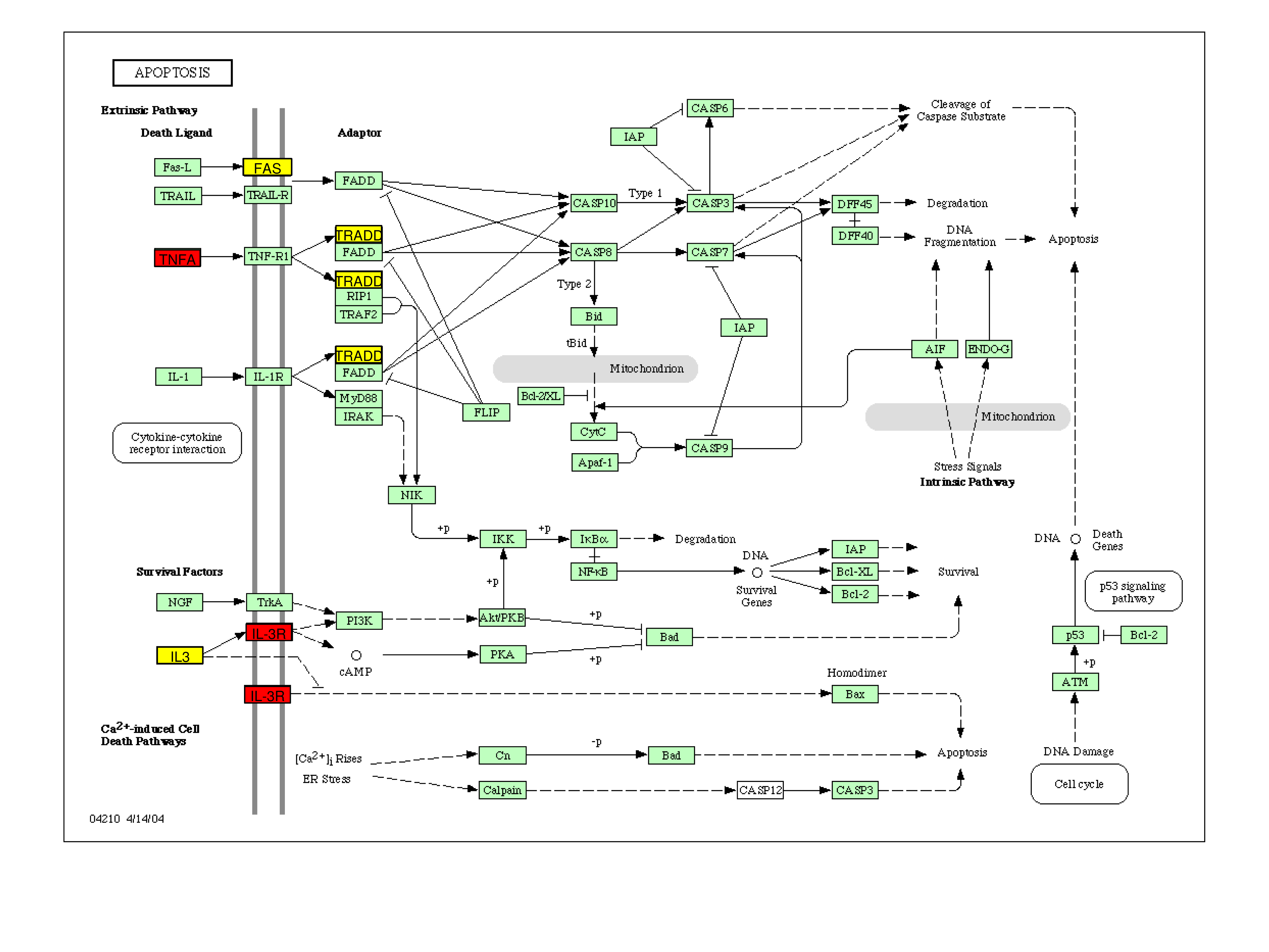

Supplement: Figure S4 — Apoptosis pathway. (0.47 MB TIF) [file pgen.1000144.s004.tif]

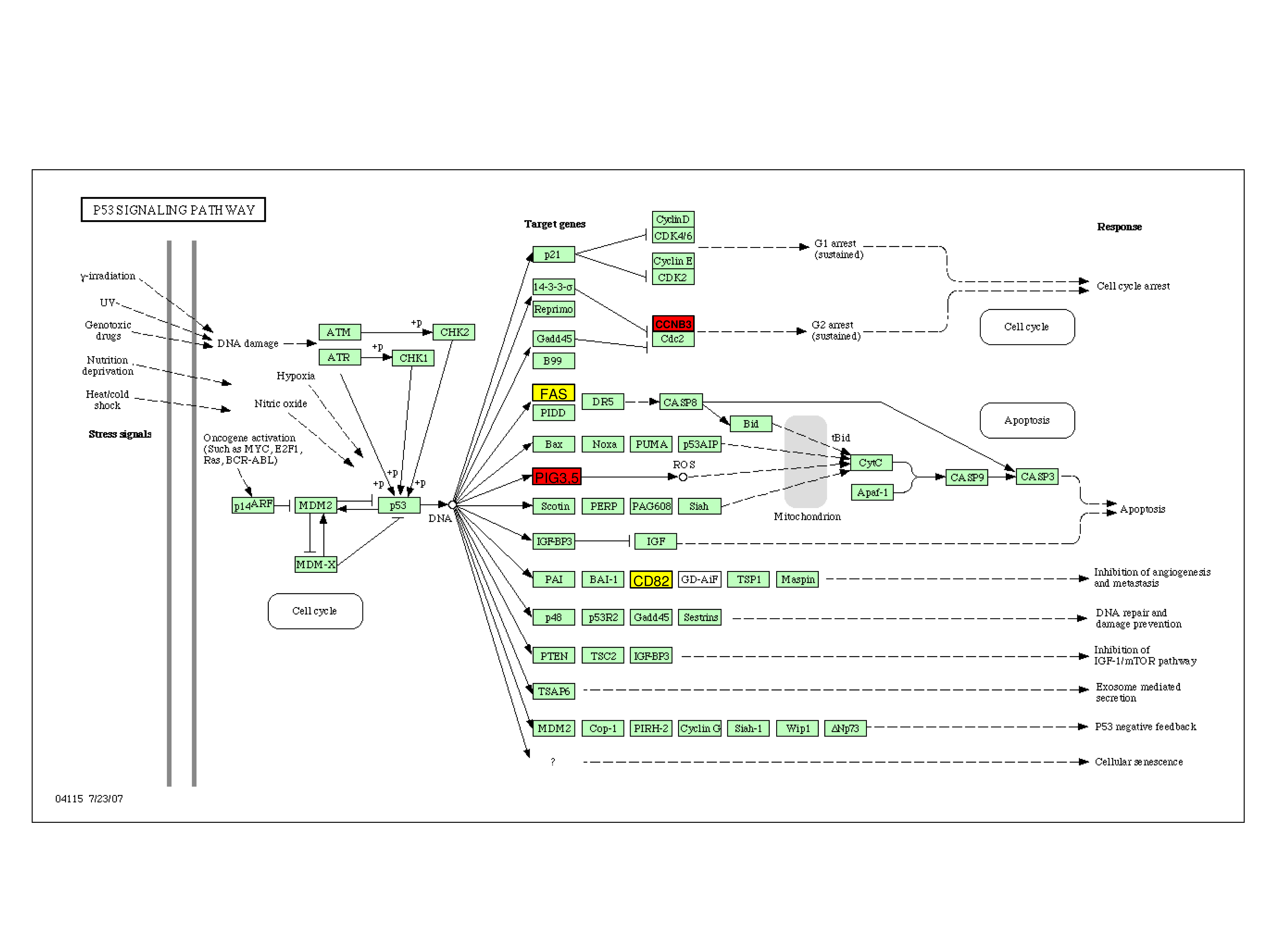

Supplement: Figure S5 — p53 signaling pathway. (0.45 MB TIF) [file pgen.1000144.s005.tif]

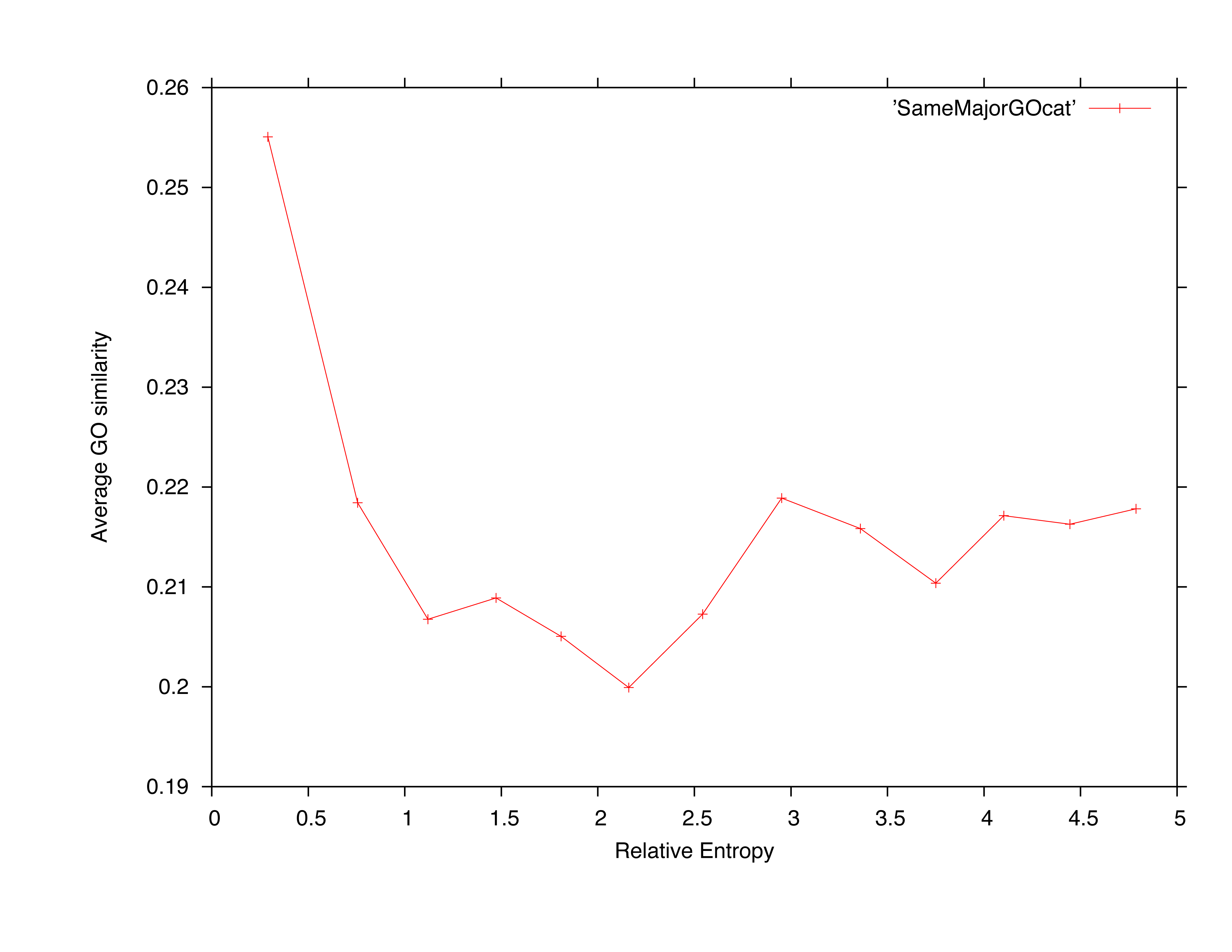

Supplement: Figure S6 — GO similarity. (0.29 MB TIF) [file pgen.1000144.s006.tif]

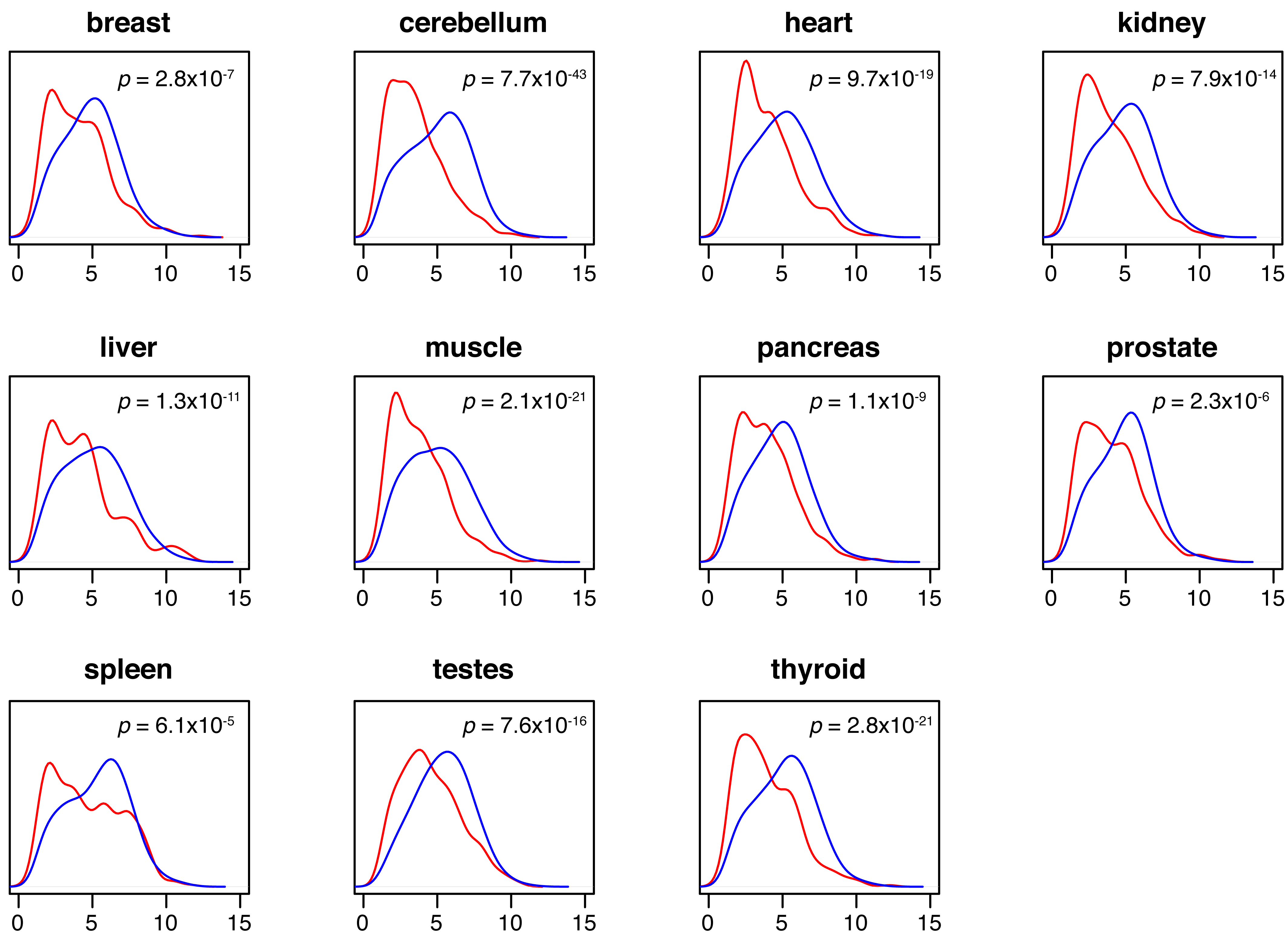

Supplement: Figure S7 — Expression results for all tissues. (0.50 MB TIF) [file pgen.1000144.s007.tif]
