# Supplementary material for: Patterns of Positive Selection in Six Mammalian Genomes
Source: PLoS Genet. 2008 Aug 1;4(8):e1000144. doi: 10.1371/journal.pgen.1000144 (PMC2483296; doi:10.1371/journal.pgen.1000144)
Supplement: Table S1 — Minimum species configurations required for likelihood ratio tests. (0.02 MB PDF) [file pgen.1000144.s008.pdf]

Table S1: Minimum species configurations required for likelihood ratio tests.

LRT	required species	ortholog groups
A: all branches	human, 2 other	16,529
B: primate branch	human, macaque, mouse or rat, dog	9,566
C: primate clade	human, chimp or macaque, 1 other	14,425
D: rodent branch	human, mouse, rat, dog	10,762
E: rodent clade	human, mouse, rat	8,991
F: human lineage	human, chimp, 1 other	14,558
G: chimp lineage	human, chimp, 1 other	14,558
H: hominid branch	human, chimp, macaque, 1 other	10,980
K: macaque lineage	human, macaque, mouse or rat or dog	12,499
