# Supplementary material for: Patterns of Positive Selection in Six Mammalian Genomes
Source: PLoS Genet. 2008 Aug 1;4(8):e1000144. doi: 10.1371/journal.pgen.1000144 (PMC2483296; doi:10.1371/journal.pgen.1000144)
Supplement: Table S2 — GO categories over-represented among predicted PSGs. (0.11 MB PDF) [file pgen.1000144.s009.pdf]

Table S2: GO categories over-represented among predicted PSGs.

		Gene number			Fold	*P*-value	*P*-value
**Category**	**Description**	**All**	**PSGs**	**E[PSGs]**	**Enrich.**	**MWU**	**FET**
	Biological process						
GO:0007606	sensory perception of chemical stimulus	255	24	6.2	3.9	**4.3×10^{-39}**	**1.5×10^{-08}**
GO:0006955	immune response	457	55	11.1	5.0	**1.4×10^{-33}**	**1.4×10^{-22}**
GO:0007608	sensory perception of smell	229	15	5.5	2.7	**6.1×10^{-31}**	4.8×10^{-04}
GO:0050896	response to stimulus	1887	101	45.7	2.2	**4.4×10^{-26}**	**1.1×10^{-14}**
GO:0002376	immune system process	608	59	14.7	4.0	**6.7×10^{-25}**	**1.7×10^{-19}**
GO:0006952	defense response	412	34	10.0	3.4	**1.3×10^{-20}**	**9.8×10^{-10}**
GO:0051707	response to other organism	138	13	3.3	3.9	**3.8×10^{-15}**	6.0×10^{-05}
GO:0007600	sensory perception	559	31	13.5	2.3	**1.1×10^{-12}**	**1.7×10^{-05}**
GO:0002526	acute inflammatory response	55	11	1.3	8.3	**4.7×10^{-11}**	**6.7×10^{-08}**
GO:0050909	sensory perception of taste	25	8	0.6	13.2	**1.4×10^{-10}**	**8.3×10^{-08}**
GO:0009611	response to wounding	321	23	7.8	3.0	**3.2×10^{-10}**	**6.9×10^{-06}**
GO:0051704	multi-organism process	222	17	5.4	3.2	**3.3×10^{-10}**	4.7×10^{-05}
GO:0006954	inflammatory response	233	20	5.6	3.5	**8.4×10^{-10}**	**1.6×10^{-06}**
GO:0002541	activation of plasma proteins during acute inflammatory response	28	7	0.7	10.3	**1.3×10^{-09}**	**3.5×10^{-06}**
GO:0006956	complement activation	28	7	0.7	10.3	**1.3×10^{-09}**	**3.5×10^{-06}**
GO:0009607	response to biotic stimulus	188	14	4.5	3.1	**1.7×10^{-09}**	3.2×10^{-04}
GO:0045087	innate immune response	70	8	1.7	4.7	**1.9×10^{-09}**	3.7×10^{-04}
GO:0009615	response to virus	69	7	1.7	4.2	**3.0×10^{-08}**	2.4×10^{-03}
GO:0009617	response to bacterium	67	6	1.6	3.7	**4.2×10^{-08}**	7.9×10^{-03}
GO:0002682	regulation of immune system process	60	11	1.5	7.6	**8.5×10^{-08}**	**1.0×10^{-06}**
GO:0016064	immunoglobulin mediated immune response	36	8	0.9	9.2	**1.1×10^{-07}**	**1.5×10^{-05}**
GO:0042742	defense response to bacterium	61	5	1.5	3.4	**1.2×10^{-07}**	2.2×10^{-02}
GO:0002253	activation of immune response	40	9	1.0	9.3	**1.2×10^{-07}**	**1.9×10^{-06}**
GO:0007186	G-protein coupled receptor protein signaling pathway	792	39	19.2	2.0	**1.4×10^{-07}**	2.5×10^{-05}
GO:0002252	immune effector process	69	11	1.7	6.6	**1.5×10^{-07}**	**3.0×10^{-06}**
GO:0006959	humoral immune response	56	9	1.4	6.6	**1.6×10^{-07}**	**7.2×10^{-06}**
GO:0002684	positive regulation of immune system process	49	9	1.2	7.6	**1.7×10^{-07}**	**8.4×10^{-06}**
GO:0050778	positive regulation of immune response	49	9	1.2	7.6	**1.7×10^{-07}**	**8.4×10^{-06}**
GO:0050776	regulation of immune response	59	11	1.4	7.7	**1.8×10^{-07}**	**1.0×10^{-06}**
GO:0002455	humoral immune response mediated by circulating immunoglobulin	24	6	0.6	10.3	**3.0×10^{-07}**	**1.8×10^{-05}**
GO:0019724	B cell mediated immunity	37	8	0.9	8.9	**3.2×10^{-07}**	**1.8×10^{-05}**
GO:0006968	cellular defense response	55	5	1.3	3.8	**3.5×10^{-07}**	1.1×10^{-02}
GO:0019882	antigen processing and presentation	27	4	0.7	6.1	**5.7×10^{-07}**	5.0×10^{-03}
GO:0006958	complement activation, classical pathway	23	6	0.6	10.8	**6.1×10^{-07}**	**1.4×10^{-05}**
GO:0050877	neurological process	811	34	19.6	1.7	**7.5×10^{-07}**	1.5×10^{-03}
GO:0006957	complement activation, alternative pathway	11	2	0.3	7.5	**1.5×10^{-06}**	2.8×10^{-02}
GO:0019835	cytolysis	15	3	0.4	8.3	**2.2×10^{-06}**	5.2×10^{-03}
GO:0051240	positive regulation of multicellular organismal process	61	10	1.5	6.8	**2.6×10^{-06}**	**1.1×10^{-05}**
GO:0002449	lymphocyte mediated immunity	53	10	1.3	7.8	**5.7×10^{-06}**	**2.5×10^{-06}**
GO:0009605	response to external stimulus	462	27	11.2	2.4	**5.8×10^{-06}**	4.2×10^{-05}
GO:0002460	adaptive immune response based on somatic recombination of immune receptors built from immunoglobulin superfamily domains	57	13	1.4	9.4	**8.0×10^{-06}**	**1.1×10^{-08}**
GO:0002250	adaptive immune response	58	13	1.4	9.3	**1.5×10^{-05}**	**1.1×10^{-08}**
GO:0007338	single fertilization	39	4	0.9	4.2	**1.7×10^{-05}**	1.4×10^{-02}
	Molecular function						
GO:0004984	olfactory receptor activity	229	15	5.5	2.7	**6.9×10^{-36}**	4.8×10^{-04}
GO:0001584	rhodopsin-like receptor activity	540	30	13.1	2.3	**8.4×10^{-18}**	2.2×10^{-05}
GO:0004930	G-protein coupled receptor activity	625	37	15.1	2.4	**2.5×10^{-14}**	**5.1×10^{-07}**
GO:0004888	(*) transmembrane receptor activity	972	55	23.5	2.3	**4.0×10^{-12}**	**3.3×10^{-09}**
GO:0004872	(*) receptor activity	1411	89	34.1	2.6	**2.5×10^{-10}**	**1.9×10^{-17}**
GO:0008527	taste receptor activity	14	5	0.3	14.8	**1.3×10^{-08}**	**1.4×10^{-05}**
GO:0008009	chemokine activity	34	5	0.8	6.1	**3.7×10^{-07}**	1.3×10^{-03}
GO:0042379	chemokine receptor binding	34	5	0.8	6.1	**3.7×10^{-07}**	1.3×10^{-03}
GO:0030414	protease inhibitor activity	110	6	2.7	2.3	**4.1×10^{-07}**	5.1×10^{-02}
GO:0004866	endopeptidase inhibitor activity	110	6	2.7	2.3	**4.1×10^{-07}**	5.1×10^{-02}

GO:0019965	interleukin binding	33	1	0.8	1.3	$\mathbf{2.2 \times 10^{-06}}$	5.5×10^{-01}
GO:0005125	cytokine activity	184	13	4.5	2.9	$\mathbf{7.8 \times 10^{-06}}$	5.8×10^{-04}
GO:0008173	RNA methyltransferase activity	19	1	0.5	2.2	$\mathbf{8.4 \times 10^{-06}}$	3.7×10^{-01}
GO:0004907	interleukin receptor activity	28	1	0.7	1.5	$\mathbf{2.1 \times 10^{-05}}$	5.0×10^{-01}
GO:0017171	serine hydrolase activity	150	9	3.6	2.5	$\mathbf{2.1 \times 10^{-05}}$	1.1×10^{-02}
	Cellular component						
GO:0005576	extracellular region	889	43	21.5	2.0	$\mathbf{2.6 \times 10^{-11}}$	4.5×10^{-05}
GO:0005615	extracellular space	354	19	8.6	2.2	$\mathbf{6.8 \times 10^{-08}}$	4.1×10^{-03}
GO:0042611	MHC protein complex	14	4	0.3	11.8	$\mathbf{2.8 \times 10^{-07}}$	2.8×10^{-04}
GO:0031224	(*) intrinsic to membrane	3817	168	92.4	1.8	$\mathbf{7.7 \times 10^{-06}}$	$\mathbf{1.4 \times 10^{-16}}$
GO:0042612	MHC class I protein complex	12	2	0.3	6.9	$\mathbf{8.9 \times 10^{-06}}$	3.3×10^{-02}
GO:0016021	(*) integral to membrane	3799	168	91.9	1.8	$\mathbf{8.9 \times 10^{-06}}$	$\mathbf{1.0 \times 10^{-16}}$

Bold indicates FWER $<$0.05 (Holm correction).
