# Supplementary material for: Patterns of Positive Selection in Six Mammalian Genomes
Source: PLoS Genet. 2008 Aug 1;4(8):e1000144. doi: 10.1371/journal.pgen.1000144 (PMC2483296; doi:10.1371/journal.pgen.1000144)
Supplement: Table S3 — PANTHER categories over-represented among predicted PSGs. (0.10 MB PDF) [file pgen.1000144.s010.pdf]

Table S3: PANTHER categories over-represented among genes predicted to be under positive selection.

		**Gene number**			**Fold**	***P*-value**	***P*-value**
**Category**	**Description**	**All**	**PSGs**	**E[PSGs]**	**Enrich.**	**MWU**	**FET**
	Biological process						
BP00148	Immunity and defense	968	76	23.4	3.2	**2.2×10^{-25}**	**3.0×10^{-20}**
BP00184	Olfaction	122	8	3.0	2.7	**8.6×10^{-15}**	9.6×10^{-03}
BP00183	Chemosensory perception	128	8	3.1	2.6	**1.3×10^{-13}**	1.3×10^{-02}
BP00155	Macrophage-mediated immunity	97	10	2.3	4.3	**4.6×10^{-09}**	**1.2×10^{-04}**
BP00149	T-cell mediated immunity	134	15	3.2	4.6	**1.2×10^{-08}**	**8.7×10^{-07}**
BP00157	Natural killer cell mediated immunity	35	7	0.8	8.3	**1.6×10^{-08}**	**1.7×10^{-05}**
BP00107	Cytokine and chemokine mediated signaling pathway	192	10	4.6	2.2	**4.1×10^{-08}**	1.9×10^{-02}
BP00255	Cytokine/chemokine mediated immunity	88	7	2.1	3.3	**7.6×10^{-08}**	5.5×10^{-03}
BP00176	Blood clotting	69	4	1.7	2.4	**2.2×10^{-07}**	8.6×10^{-02}
BP00152	B-cell- and antibody-mediated immunity	73	16	1.8	9.1	**4.8×10^{-07}**	**1.5×10^{-11}**
BP00156	Interferon-mediated immunity	56	7	1.4	5.2	**8.0×10^{-07}**	3.8×10^{-04}
BP00153	Complement-mediated immunity	40	7	1.0	7.2	**6.0×10^{-06}**	**4.3×10^{-05}**
BP00150	MHCI-mediated immunity	12	2	0.3	6.9	**1.6×10^{-05}**	3.3×10^{-02}
BP00151	MHCII-mediated immunity	12	3	0.3	10.3	**3.4×10^{-05}**	2.6×10^{-03}
BP00288	Granulocyte-mediated immunity	47	7	1.1	6.2	**3.4×10^{-05}**	1.3×10^{-04}
BP00240	Fertilization	26	4	0.6	6.4	**6.2×10^{-05}**	3.3×10^{-03}
BP00299	Steroid hormone metabolism	21	3	0.5	5.9	**8.3×10^{-05}**	1.4×10^{-02}
	Molecular function						
MF00173	Defense/immunity protein	223	46	5.4	8.5	**1.4×10^{-35}**	**9.7×10^{-30}**
MF00004	Immunoglobulin receptor family member	66	27	1.6	16.9	**4.4×10^{-19}**	**1.0×10^{-26}**
MF00224	KRAB box transcription factor	409	9	9.9	0.9	**4.2×10^{-13}**	6.6×10^{-01}
MF00018	Chemokine	29	4	0.7	5.7	**2.0×10^{-08}**	5.0×10^{-03}
MF00102	Protease inhibitor	97	3	2.3	1.3	**1.1×10^{-07}**	4.2×10^{-01}
MF00216	Serine protease	140	10	3.4	3.0	**1.6×10^{-06}**	2.2×10^{-03}
MF00250	Serine protease inhibitor	63	2	1.5	1.3	**7.5×10^{-06}**	4.5×10^{-01}
MF00175	Major histocompatibility complex antigen	15	4	0.4	11.0	**1.1×10^{-05}**	3.7×10^{-04}
MF00177	Other defense and immunity protein	50	4	1.2	3.3	**1.7×10^{-05}**	3.2×10^{-02}
MF00174	Complement component	35	6	0.8	7.1	**3.6×10^{-05}**	1.7×10^{-04}
MF00176	Antibacterial response protein	26	1	0.6	1.6	**5.8×10^{-05}**	4.7×10^{-01}

Bold indicates FWER <0.05 (Holm correction).
