# Supplementary material for: Patterns of Positive Selection in Six Mammalian Genomes
Source: PLoS Genet. 2008 Aug 1;4(8):e1000144. doi: 10.1371/journal.pgen.1000144 (PMC2483296; doi:10.1371/journal.pgen.1000144)
Supplement: Text S1 — Supplementary methods with complete set of supplementary figures and tables (including some not referenced in main article). (2.86 MB PDF) [file pgen.1000144.s011.pdf]

Patterns of positive selection on the mammalian tree
Supplementary Material

C. Kosiol, T. Vinar, R. R. Da Fonseca, M. J. Hubisz, C. D. Bustamante, R. Nielsen, A. Siepel

Text S1

1 Ortholog identification

To accurately identify orthologous sequences in the six mammalian species, we used syntenic whole-genome alignments produced by the MULTIZ program [1] on the following assemblies available through the UCSC genome browser [2]: human (hg18), chimpanzee (panTro2), macaque (rheMac2), mouse (mm8), rat (rn4), and dog (canFam2). The human genome was used as a reference genome. We used the pairwise syntenic nets [3] with respect to human genome as an input to MULTIZ pipeline to reduce the likelihood of paralogous alignments. We compensated for low sequence quality in some regions by masking the bases in chimpanzee, macaque, rat, and dog with sequence quality lower than 20.

To identify genes within these alignments, we used the union of all human annotated genes in RefSeq [4], UCSC Known Genes [5], and VEGA [6], downloaded from the UCSC genome browser on February 19, 2007. We discarded transcripts without annotated coding regions (CDSs), CDSs shorter than 100 bp, as well as CDSs with length other than multiple of three. Clustering of the remaining 88,879 transcripts (some of them may be duplicates from different annotation databases) resulted in an initial set of 21,115 gene clusters in the human genome.

We then used our syntenic alignments to map these genes to other five mammalian species. For the purpose of the positive selection analysis, we are interested in a high-confidence set of orthologous alignments. To ensure the quality of our data set, we have subjected the resulting alignments to a series of filters designed to minimize impact of annotation errors, sequence quality, and changes in gene structure on subsequent analysis. In particular, we require that: (1) a transcript maps to the non-human genome via a single chain of sequence alignments that includes at least 80% of CDS (as determined by building process of UCSC syntenic maps), (2) no more than 10% of CDS falls into known sequencing gaps or low quality sequence in the non-human genome, (3) there are no frame-shifts in the non-human CDS, unless they are compensated for within 15 bases, (4) and there are no in-frame stop codons in the non-human CDS, and all splice sites are conserved.

In an earlier study [7], we have found that start sites and stop codons are often not conserved between species. Therefore, we also consider incomplete transcripts, where we remove $\sim$10% of coding sequence from both ends of the complete transcript, and subject them to the same series of filters.

For each gene cluster we have selected the transcript (complete or incomplete) that has been successfully mapped to the largest number of species, using CDS length as a secondary criteria. This procedure resulted in 17,500 genes that were successfully mapped to at least two additional species besides human. For technical reasons, we have removed 11 additional transcripts, obtaining orthologous multiple alignments for 17,489 genes, with average of $\sim$4 non-human orthologs per gene. The number of genes in this data set for each species is shown in Table 1.

For recently duplicated genes it is impossible to establish 1:1 orthologous relationships of human gene and its non-human lineage ortholog. Moreover, these genes often occur in tandem clusters, where it is hard to distinguish orthologous from paralogous relationships. We have developed a pairwise test for these recent duplications (i.e., duplication occurring after split from the non-human species lineage), and to avoid potential false positives, we have removed from our set orthologs that have such a recent non-pseudogenized

duplication in either human lineage or in the non-human lineage. (Note that we do not necessarily remove the affected gene completely from the analysis. For example, if there is a recent duplication specific to the dog lineage, we can still use orthologs of the same gene in chimp, macaque, mouse and rat.)

To detect recent duplications, we used a collection of gene predictions from UCSC genome browser using the following sources: RefSeq (human, chimpanzee, mouse, rat, dog), transmapped RefSeq (macaque), N-scan predictions (all species), ENSEMBL predictions (human, mouse, rat), and UCSC known genes (human, mouse, rat).

For each of the 17,500 genes that previously passed our filters, and in each species where the gene was successfully mapped, we used nucleotide blast to locate the closest non-overlapping gene prediction with alignment covering at least 80% of the CDS. If a gene in the human genome has a closer match within the human than to its counterpart in the non-human species mapped through syntenic alignment, we detected a duplication on human lineage that is recent with respect to that non-human species. Similarly, if the counterpart of the human gene (mapped through syntenic alignment) in non-human genome has closer match among gene predictions within the same genome than to its corresponding human gene, we detected a recent duplication on that other species lineage that is recent with respect to that species. When comparing distances, we apply a simple correction based on Jukes-Cantor distance to compensate for faster evolution on rodent lineage.

2 Likelihood ratio tests for positively selected genes

2.1 Effect of pooling genes by G+C class

As noted in the text, the genes were partitioned into 8 equally sized groups by third-codon-position G+C content. The branch length proportions and the transition/transversion rate ratio κ were estimated by pooling the data within each class, and only an overall scale factor for branch lengths (μ) and the selection parameters ($\omega_0, \omega_1, p_0, p_1$) were estimated per gene. This strategy was intended to improve computational efficiency, by substantially decreasing the number of parameters to be estimated per gene. It also had the potential to increase the power of the test, by reducing the number of degrees of freedom in the LRT. However, if the assumptions of equal branch-length proportions and equal κ values per G+C class are not well-supported by the data, it could potentially lead to inflated false-positive rates and/or reduced power. Here, we investigate by simulation whether or not this parameterization influences either the power or the false-positive rates of the LRTs.

We selected 250 genes randomly from each of the 8 G+C classes, for a total of 2,000 genes representing the full set of 17,489. For each of these 2,000 genes, we estimated κ and a complete set of branch lengths using model 1a [8]. We stored these sets of parameter estimates, along with the length of each alignment, in a list $\mathcal{L}_C$ for each G+C class C. We then sampled parameter sets (with replacement) 2,000 times from each list $\mathcal{L}_C$ and used them to generate six-species synthetic alignments with *evolver* [9], assuming a constant ω among lineages and sites (model M0). This process was repeated for several different values of ω, so that no assumption about the distribution of this parameter was required. Thus, for each G+C class and each ω we obtained 2,000 simulated data sets, with lengths, branch-length proportions, and κ values reflecting those of the corresponding G+C class in the original data set.

The all-branch LRT was then applied to these simulated alignments using two different strategies (separately for each value of ω): (i) by estimating κ and the branch-length proportions once per G+C class, then estimating only the remaining parameters per gene, as for the main analysis; and (ii) by estimating all parameters per gene. Each synthetic gene was then predicted as a PSG if its (empirical) P-value was less than a designated threshold, and a non-PSG otherwise. The fraction of genes predicted to be PSGs (the sensitivity or power for $\omega > 1$ and the false positive rate for $\omega \leq 1$) was then plotted as a function of ω, separately for strategies (i) and (ii) (Fig. S8). These experiments indicated that pooling by G+C class has almost no effect

on the sensitivity or specificity of the test. Similar results are seen if sensitivity and specificity are examined together in a receiver-operating characteristic (ROC) curve (Fig. S9). Thus, G+C pooling seems to have essentially no cost in terms of false positive rates or power, but it has the benefit of a substantial savings in computation, by reducing the number of parameters to be estimated per gene (by nine, in this case).

3 Calculation of P-values using chi-square and empirical distributions

For the LRT for positive selection on all branches of the phylogeny, we used empirical P-values derived from simulations. Ideally, a similar approach would also be used for the branch and clade LRTs. However, the simulation of the empirical distributions is computationally expensive, and to save thousands of processor-hours of simulation time, we have instead assumed a 50:50 mixture of a $\chi^2_{df=1}$ distribution and a point mass at zero for these LRTs, as suggested by Zhang et al. [10].

Here, we examine this assumption by comparing empirical and chi-square-derived P-values for a specific branch (the one leading to macaque), and a specific clade (the primates). As with the all-branch test (see Methods), 10,000 alignments were simulated under model 1a, log-likelihood ratios were calculated for the appropriate lineage or clade-specific models, and (nominal) empirical P-values were derived from these models. Figure S10 shows that the χ^2-based P-values are generally conservative for both LRTs. This is true even when a 50:50 mixture of a $\chi^2_{df=1}$ distribution and point mass at zero is used; $\chi^2_{df=1}$ and $\chi^2_{df=2}$ distributions are more conservative. Thus, the use of the approximation will not produce an inflated false positive rate, but it could result in some spurious false negatives. Nevertheless, we find that the approximation has little effect on the set of PSGs identified for the analysis. If the empirical P-values are used in place of the χ^2-based P-values, the number of identified PSGs changes only by four (primate clade) or two (macaque branch).

4 Analysis of over-represented functional categories

4.1 Possible bias from alignment depth

A possible concern with the enrichment analysis is that it might reflect differences between categories in the alignment depth per gene. For example, if genes in the "sensory perception of smell" category are significantly more likely than other genes to have full six-species alignments (as opposed to alignments of three, four, or five species, due to recent duplication, low sequence quality, etc.), then an enrichment for PSGs in this category might simply reflect an increase in power for PSG detection.

To examine this possibility, we divided all alignments into high-depth (five or six species) and low-depth (three or four species) classes, and looked for functional enrichments within each class. We found that there are significant differences in the functional categories of high- and low-depth genes, but these differences cannot explain the PSG enrichments. In particular, the categories that are over-represented among high-depth genes generally relate to housekeeping and development functions, and are essentially disjoint with the PSG-enriched categories. Many of the PSG-enriched categories relating to sensory perception are actually quite strongly enriched in low-depth genes, for which the power to detect positive selection will be reduced—a trend that runs in the opposite direction of what would be required for the PSG enrichments to be an artifact of differences in power. Accordingly, if the analysis of functional enrichments for PSGs is redone with just the high-depth genes, the immunity and defense categories still are found to be highly enriched. The sensory perception genes are no longer significantly enriched, but this is probably because there are many fewer of them among the high-depth genes. In summary, we find no evidence that the observed enrichments for PSGs are related to differences in detection power due to alignment depth. Recent

duplication likely contributes to the enrichments for sensory perception among both PSGs and low-depth genes.

We also examined the possibility that the observed differences in the functional categories identified by the primate- and rodent-clade LRTs might be artifacts of differences in alignment depth. We found that, even when only high-depth genes are considered, the MWU test for the primate-clade LRT primarily identifies categories related to sensory perception, while the MWU test for the rodent-clade LRT primarily identifies categories related to immunity and defense. The results are similar if only genes that include both rodent species are considered, confirming that the enrichment for sensory perception categories in primates is not simply due to an absence of rodent data for genes in these categories (for example, because of recent duplications).

4.2 Possible bias from gene length

A similar possibility is that differences in gene length (i.e., total coding sequence length) could produce spurious functional enrichments, because the power to detect positive selection depends on length (see Fig. 6) and the genes assigned to different GO categories sometimes have quite different length distributions. We examined this possibility in two ways. First, if gene length were driving the detection of PSGs, then a pronounced difference in the length distributions of PSGs and non-PSGs would be expected. In fact, these distributions are highly similar (Fig. S11) and the null hypothesis that both sets of lengths are drawn from the same distribution cannot be rejected ($P = 0.33$, one-sided MWU). This suggests that it is unlikely that length is a dominant factor in the analysis. Second, we examined the length distributions of the enriched categories of genes (Table 2), to see whether gene length might be an important factor for particular categories of interest. By a MWU test comparing the length distribution within each category with that of other genes, most categories enriched for PSGs did not contain significantly longer genes than other categories. Indeed, seven PSG-enriched categories—including 'cytokine activity,' 'olfactory receptor activity,' and 'response to bacterium'—showed a significant shift toward shorter genes in their length distributions. However, there were two categories of genes—'integral to membrane' and 'transmembrane receptor activity' (marked by asterisks in Table 2)—that did show significant shifts toward longer genes, and it is possible that the enrichments for these GO categories are influenced by increased detection power. These categories have not been discussed at length in the paper, and this finding of a possible bias does not alter the main conclusions of our functional analysis.

5 Bayesian inference of selection histories

5.1 Justification of the model

Gains and losses of positive selection would ideally be modeled in continuous time, and would be allowed to occur at any point along branches (see, e.g., [11]). However, making inferences about the quantities of interest here (e.g., numbers of genes under selection on particular branches, the probability that each gene has an episodic history) would require a complex model and would be computationally prohibitive at the scale of our analysis. In addition, it is likely that our six-species data set would provide only weak information about mid-branch gains and losses. Instead, our model attempts to exploit the discrete-branch nature of the Yang-Nielsen branch-site models, by defining a simple switching process that induces a probability distribution over the finite set of selection histories considered by those models. As noted in the text, this approach allows the likelihood computation and inference of selection histories to be decoupled (if the maximum likelihood values for codon-model parameters are assumed), making it possible to apply the method efficiently to large sets of genes.

To explain every possible branch-wise selection history by a parsimonious set of switches, there must be switch points for both "early" and "late" switches on internal branches of the tree. A single early switch point is sufficient for external branches. To see why two switches are needed, suppose only early switches are allowed (i.e., a switch is possible at the ancestral end of each branch). Such a model will allow any selection history to be determined by a set of switches (each branch is simply switched "on" or "off," as necessary, to be consistent with a given history), but the set of switches will not be parsimonious. The problem arises when the branch ancestral to an internal node has one selective mode and the two branches that descend from the node have the other selective mode. In this case, it is far more plausible biologically that a single change in selection occurred soon before the divergence of the descendant species, than that two independent (and convergent) events occurred in the descendant species, as will be required by the early-switch model. Redefining the model so that events occur late rather than early does not solve the problem, because this requires that both descendant branches always have the same mode, and therefore cannot capture all histories. Allowing a single switch at the midpoint of each branch also is problematic, because it leads to branches that are only partly under selection, which is not compatible with the Yang-Nielsen models. The introduction of early and late switch points allows all selection histories to be explained parsimoniously, in a way that is fully compatible with a discrete-branch selection model.

It is worth noting that, unlike the early-switch model, the double-switch model does not allow a set of switches to be uniquely determined by a selection history. For example, a single switch on an ancestral branch could be replaced by two switches on descendant branches. Even more unlikely, three switches could be introduced in place of zero switches, where all three branches adjoining an internal node have the same selective mode. To address this problem, we invoke the parsimony principle, and select the history with the fewest switches (which is unique; see below). We justify this decision by noting that all nonparsimonious scenarios require at least one additional switch within the set of switch points surrounding a single internal node. Thus, these scenarios represent cases of multiple rare events occuring during a short time period, and it is reasonable to assume that their probabilities are quite small.

Finally, the issue of the root of the phylogeny deserves further comment. As noted in the text, the selection histories are defined with respect to an unrooted tree, and their likelihoods are evaluated accordingly. However, the switching model actually requires a root, because branches must be oriented in order to define gains and losses. Because the tree is believed to be rooted on the branch to dog (e.g., [12]), and the primate-rodent and (primate,rodent)-dog divergences are believed to have occurred relatively near one another in evolutionary time (making the branch between the corresponding nodes in the phylogeny fairly short), we address this problem by treating the primate-rodent most recent common ancestor as the root of the tree, and all three adjoining branches as descendants.

5.2 Mapping selection histories to switches

Here we describe the algorithm for defining a unique, parsimonious set of switches corresponding to a particular selection history Z. As in the text, let $Z_b \in \{0, 1\}$ represent the selective mode of branch $b \in \{1, \ldots, B\}$. In addition, let the switch point for internal node $n \in \mathcal{N}$ and adjoining branch $b \in \mathcal{B}_n$ be denoted P_{nb}, and let $V_{nb} \in \{0, 1\}$ and $W_{nb} \in \{0, 1\}$ indicate the selective states before and after point P_{nb}, respectively. Let (U_{nb}, T_{nb}) be a permutation of (V_{nb}, W_{nb}) such that U_{nb} represents the *internal* state (i.e., near node n) and T_{nb} represents the *external* state (i.e., away from n). That is,

$$(U_{nb}, T_{nb}) = \begin{cases} (W_{nb}, V_{nb}) & b \text{ is ancestral} \\ (V_{nb}, W_{nb}) & b \text{ is descendant} \end{cases} \quad (1)$$

Because there can be no switches between switchpoints, it must be true that $U_{nb} = U_{nb'}$ for all b and b' adjacent to the same node n. Similarly, external states are determined by the states of branches in the

selection history; that is, $T_{nb} = Z_b$ for all n and b. Thus, the U_{nb} variables, together with the Z_b variables, completely determine the switches. Because all U_{nb} must be equal for a given n, only one value is needed for each n, call it U_n. It is not hard to see that setting each U_n to the majority value of the flanking Z_b variables must result in a parsimonious set of switches; that is $U_n = I(\sum_{b\in\mathcal{B}_n} Z_b \geq 2)$. Setting U_n this way requires at most one switch per internal node, while setting it otherwise would require at least two.

The algorithm, then, is simply to set each U_n to the majority value of the adjoining Z_b variables, and to set each T_{nb} equal to the corresponding Z_b. The (V_{nb}, W_{nb}) variables can be determined by permuting the corresponding (U_{nb}, T_{nb}) variables.

5.3 Gibbs sampling algorithm

As described in the text, the complete data likelihood under the hierarchical Bayes model is,

$$P(\mathbf{X}, \mathbf{Z}, \boldsymbol{\theta}) = P(\boldsymbol{\theta}) \prod_{i=1}^{N} P(X_i|Z_i)P(Z_i|\boldsymbol{\theta}), \tag{2}$$

with

$$\begin{aligned} P(\boldsymbol{\theta}) &= \text{Beta}(\theta_0|\alpha,\beta) \prod_{n\in\mathcal{N}} \prod_{b\in\mathcal{B}_n} \prod_{e\in\{G,L\}} \text{Beta}(\theta_{nbe}|\alpha,\beta) \\ P(Z_i|\boldsymbol{\theta}) &= \theta_0^{U_{i0}}(1-\theta_0)^{1-U_{i0}} \prod_{n\in\mathcal{N}} \prod_{b\in\mathcal{B}(n)} \left[\theta_{nbG}^{W_{inb}}(1-\theta_{nbG})^{1-W_{inb}}\right]^{1-V_{inb}} \left[\theta_{nbL}^{1-W_{inb}}(1-\theta_{nbL})^{W_{inb}}\right]^{V_{inb}} \\ P(X_i|Z_i = j) &= \exp(L_{ij}), \end{aligned}$$

where N is the number of genes, M is the number of histories, $\mathbf{X} = (X_1, \ldots, X_N)$ is the set of alignments, $\mathbf{Z} = (Z_1, \ldots, Z_N)$ is the set of histories, $\mathcal{N}$ is the set of internal nodes, $\mathcal{B}_n$ is the set of branches adjoining internal node n, U_{i0} is the selective state at the root for gene i, (V_{inb}, W_{inb}) are the selective states before and after switch point P_{nb} for gene i, $\boldsymbol{\theta} = \{\theta_{nbe} : n \in \mathcal{N}, b \in \mathcal{B}_n, e \in \{G, L\}\} \cup \{\theta_0\}$ is the set of parameters, L_{ij} is the precomputed maximum log likelihood for gene i under history j, and (α, β) defines the prior for all parameters.

Combining these equations yields:

$$\begin{aligned} P(\mathbf{X}, \mathbf{Z}, \boldsymbol{\theta}) =& \text{Beta}(\theta_0|\alpha,\beta)\, \theta_0^{U_0'}(1-\theta_0)^{N-U_0'} \exp(L') \\ &\times \prod_{n\in\mathcal{N}} \prod_{b\in\mathcal{B}(n)} \theta_{nbG}^{A_{nb}}(1-\theta_{nbG})^{B_{nb}} \theta_{nbL}^{C_{nb}}(1-\theta_{nbL})^{D_{nb}} \\ &\times \prod_{e\in\{G,L\}} \text{Beta}(\theta_{nbe}|\alpha,\beta) \end{aligned} \tag{3}$$

where $U_0' = \sum_{i=1}^N U_{i0}$, $L' = \sum_{i=1}^N L_{i,Z_i}$, $A_{nb} = \sum_{i=1}^N W_{inb}(1-V_{inb})$, $B_{nb} = \sum_{i=1}^N (1-W_{inb})(1-V_{inb})$, $C_{nb} = \sum_{i=1}^N (1-W_{inb})V_{inb}$, and $D_{nb} = \sum_{i=1}^N W_{inb}V_{inb}$.

The Gibbs sampler alternates between sampling each Z_i conditional on all other parameters and sampling each component of $\boldsymbol{\theta}$ conditional on all other parameters. The Z_is are conditionally independent of each other, and each Z_i is conditionally independent of $(X_1, \ldots, X_{i-1}, X_{i+1}, X_N)$ given X_i. Hence, the conditional distribution for Z_i is given by:

$$\begin{aligned} P(Z_i|X_i, \boldsymbol{\theta}) &= \frac{P(X_i, Z_i, \boldsymbol{\theta})}{\sum_{Z_i'} P(X_i, Z_i', \boldsymbol{\theta})} \\ &= \frac{\exp(L_{i,Z_i} - L_{i,\max})P(Z_i|\boldsymbol{\theta})}{\sum_{Z_i'} \exp(L_{i,Z_i'} - L_{i,\max})P(Z_i'|\boldsymbol{\theta})} \end{aligned} \tag{4}$$

where $P(Z_i|\boldsymbol{\theta})$ is defined as above, the $P(\boldsymbol{\theta})$ term cancels, and, to prevent underflow, the numerator and denominator are divided by $\exp(L_{i,\max})$ with $L_{i,\max} = \max_j L_{i,j}$. Thus, each Z_i can be sampled from an M-component multinomial distribution, which can be computed by calculating $\exp(L_{i,j} - L_{i,\max})P(Z_i = j|\boldsymbol{\theta})$ for all j and then normalizing.

Similarly, the components of $\boldsymbol{\theta}$ are conditionally independent of one another and of $\mathbf{X}$ given $\mathbf{Z}$. After cancellation of terms involving $\mathbf{X}$ and other elements of $\boldsymbol{\theta}$, the distribution for an individual component, say θ_{nbG} is given by:

$$\begin{aligned} P(\theta_{nbG}|\mathbf{Z}) &= \frac{\text{Beta}(\theta_{nbG}|\alpha,\beta)\,\theta_{nbG}^{A_{nb}}(1-\theta_{nbG})^{B_{nb}}}{\int \text{Beta}(\theta|\alpha,\beta)\,\theta^{A_{nb}}(1-\theta)^{B_{nb}}\,d\theta} \\ &= \text{Beta}\left(\theta_{nbG}|\alpha + A_{nb}, \beta + B_{nb}\right) \end{aligned} \tag{5}$$

Thus, θ_{nbG} can be sampled from a Beta distribution, in the ordinary way for a Bernoulli random variable with a conjugate Beta prior. We sample Beta random variables by sampling two Gamma random variables and taking their ratio. The Gamma variables are sampled using Best's rejection algorithm.

Similarly, the distributions for θ_{nbL} and θ_0 are given by:

$$P(\theta_{nbL}|\mathbf{Z}) = \text{Beta}\left(\theta_{nbL}|\alpha + C_{nb}, \beta + D_{nb}\right) \tag{6}$$

$$P(\theta_0|\mathbf{Z}) = \text{Beta}\left(\theta_0|\alpha + U_0', \beta + N - U_0'\right). \tag{7}$$

The Gibbs sampling algorithm, then, initializes $\boldsymbol{\theta}$ to an appropriate value, samples each Z_i from a multinomial distribution, computes the counts $A_{nb}, B_{nb}, C_{nb}, D_{nb}$, and U_0' from the sampled $\mathbf{Z}$, samples θ_0 and each θ_{nbe} from a Beta distribution, samples each Z_i again, and so on. Convergence of the algorithm was assessed by plotting the sampled values and $\log P(\mathbf{X}, \mathbf{Z}, \boldsymbol{\theta})$ versus the iterations of the sampler. It converges very rapidly. The sampler was run for 10,000 iterations with a generous burn-in period of 100 iteration. Multiple runs with various initializations produced essentially identical results.

5.4 Alternative switching models

In addition to the model discussed above, we examined two simpler models. The first alternative model assumed a single switching parameter θ that was shared across all branches and represented both the rate of loss and the rate of gain. The second model allowed for separate rates of gain and loss, but assumed these rates were the same on all branches. The three candidate models are therefore nested, with the two-parameter model being a special case of the full model, and the one-parameter model being a special case of the two-parameter model.

All models produced qualitatively similar results for the number of genes under selection per branch or clade (Table S4). However, the inferred rates under the full model differed significantly both for gains and losses and across branches, implying that separate parameters were warranted, and the use of the full model substantially altered the estimated numbers of genes under selection on certain branches (Table S4). Under the one-parameter model, the gain/loss rate θ was estimated at 0.13 (95% CI 0.12–0.14), while under the two parameter model, the loss and gain rates were both estimated at 0.13 (0.12–0.15). The estimated number of switches per gene was nearly identical under all models (1.6).

6 Gene expression

6.1 Measures of tissue bias

Two statistics were used to measure tissue bias. The first, denoted τ, is defined as [13]:

$$\tau = \frac{\sum_t 1 - v_t/v_{\max}}{N-1}, \tag{8}$$

where v_t is the estimated expression intensity in tissue t, $v_{\text{max}} = \max_t v_t$, and N is the number of tissues. τ represents the average difference in normalized expression intensity from that of the tissue of maximal expression. The second statistic, here denoted γ_t, is defined for each tissue t as [14]:

$$\gamma_t = \cos^2\theta = \left(\frac{v_t}{|\mathbf{v}|}\right)^2 = \frac{v_t^2}{\sum_{t'} v_{t'}^2}, \tag{9}$$

where $\mathbf{v}$ is the 11-dimensional expression vector, $|\mathbf{v}|$ is its Euclidean length, v_t is the component associated with tissue t, and θ is the angle between $\mathbf{v}$ and the coordinate axis associated with tissue t. Note that $\sum_t \gamma_t = 1$ for each gene. As a general measure of tissue bias for each gene, we use $\gamma = \max_t \gamma_t$. Both τ and γ range between 0 and 1, with larger values indicating greater tissue bias.

In defining genes as tissue specific for tissue t we required $\gamma_t > 0.25$ and $\gamma_{t'} < 0.125$ for all $t' \neq t$. Thus, the expression intensity for tissue t must be at least half of the total intensity, measured in Euclidean space ($v_t/|\mathbf{v}| > 0.5$), with $\gamma_{t'} < 0.125$ ensuring that all other tissue have substantially reduced intensities.

6.2 Expression patterns in primate and rodent PSGs

We also examined the expression patterns of genes identified by the LRTs for positive selection in the primate clade or the rodent clade. The primate PSGs showed reduced expression levels in most tissues, with statistically significant reductions in the cerebellum, kidney, spleen, and testes. Perhaps not surprisingly, the rodent PSGs and non-PSGs did not generally show significantly different (human) expression levels, except in liver and cerebellum, where expression in PSGs was slightly reduced. A modest increase in tissue bias was seen for both primate and rodent PSGs. Only a few tissue-specific genes were identified by the clade tests, so it was not possible to compare the relationships between tissue-specific expression and positive selection in primates and in rodents. However, we did find significant enrichments for primate PSGs among spleen-specific genes, and for rodent PSGs among testes-specific genes.

6.3 Possible biases in the expression analysis

A possible concern is that the finding of decreased expression levels and increased tissue bias among PSGs is a spurious result stemming from a dependency of detection power and/or false positive (FP) rates on degree of constraint at sites experiencing neutral evolution or negative selection (“background” sites). Specifically, suppose FP rates for PSG detection are higher among non-PSGs with $\omega \approx 1$ than for those with $\omega \ll 1$. In addition, suppose true PSGs are more likely to be detected if their background sites have higher average ω values, resulting in higher average ω for the genes as a whole. Finally, suppose there is a strong negative correlation between ω and expression level, and a strong positive correlation between ω and tissue bias, as has been widely reported (e.g., [15])—but suppose that this relationship can be explained completely in terms of background sites, and reflects no dependency on positive selection. In such a case, the argument goes, FPs among the PSGs would be enriched for decreased expression and increased tissue bias, and false negatives (FNs) among the non-PSGs would be enriched for increased expression and decreased tissue bias, leading to an artifactual difference in expression levels and patterns between the two classes of genes.

It is difficult to correct the analysis for background ω in a direct way, because the power to identify particular sites under positive selection is weak, and estimates of foreground and background ω are confounded with each other and with the detection of positive selection itself. Instead, we used a simple, conservative method to eliminate FPs showing low expression (or high tissue bias; these attributes were handled separately) and FNs showing high expression (or low tissue bias). We divided the genes into classes of “low” and “high” expression level (tissue bias), and then defined a new set of PSGs by using a very stringent P-value threshold for low-expression (high-bias) genes (FDR < 0.01) and a considerably less stringent threshold for high-expression (low-bias) genes (FDR < 0.1). Thus, the low-expression (high-bias) PSGs most likely to

be false positives were transferred to the non-PSG set, and the high-expression (low-bias) non-PSGs most likely to be false negatives were transferred to the PSG set. This procedure is conservative in that it necessarily diminishes the difference in expression levels and patterns between the two sets. The analysis was based on the results of the all-branch test, as was the main analysis of expression levels and patterns. The genes were partitioned into classes of "low" and "high" expression based on their median (across the 11 tissues) RMA-based expression levels, with the median of this value across all genes serving as the cutoff (i.e., the "low" and "high" classes were equal in size). Similar results were obtained using the maximum expression level across tissues for each gene, and using the expression level measured from a tissue mixture. The genes were partitioned into classes of "low" and "high" tissue-bias in a similar way based on the τ statistic.

Even after this conservative correction, significantly reduced expression levels were observed in PSGs in nearly all tissues, although in many cases the reduction is considerably less dramatic than it was before the correction was applied (Fig. S12; c.f. Fig. S7). Similarly, tissue bias remains significantly increased in PSGs relative to non-PSGs, with a less pronounced difference following the correction (Fig. S13; c.f. Fig. 5D). We conclude that an association of FPs and FNs with expression levels and patterns may partially contribute to the observed difference between PSGs and non-PSGs, but that it is unlikely to explain these differences completely.

Another concern about the expression analysis is that reduced expression levels among PSGs may simply be a consequence of increased tissue bias. That is, if genes that show a bias for expression in particular tissues have reduced average expression, and if the choice of tissue is randomly distributed across genes, then an overall reduction in the average expression level for each tissue would result. The question remains, then, whether PSGs show reduced expression levels not only on average, but in the tissues in which their expression is favored. To address this question, we found the maximum expression level across the 11 tissues for each gene, and then compared the distributions of these values for PSGs and non-PSGs. By this comparison, PSGs still show significantly lower expression levels than non-PSGs ($P = 3.6 \times 10^{-6}$; data not shown), although the difference is considerably less pronounced than for certain individual tissues such as cerebellum, muscle, and thyroid (Fig. S7). Thus, differences in tissue bias may partly explain differences in expression level, but the explanation does not appear to be complete. This question cannot be fully resolved without expression data from a larger number of tissues.

7 Estimation of population size ratios

In addition to the phylogenetic interpretation ω, we use a population genetic interpretation of ω which was first introduces by Halpern and Bruno [16] and further developed by Nielsen and Yang [17] and Thorne et al. [18]. The probability that a new mutation eventually becomes fixed is

$$\Pr(fixation) = \frac{1-e^{-2s}}{1-e^{-4Ns}} \approx \frac{2s}{1-e^{-4Ns}} \tag{10}$$

if we assume that the selection coefficient s is small and N is large and equal to the effective population size [19]. In the neutral case ($s \approx 0$) this is simply the the initial frequency of the mutation $\frac{1}{2N}$.

The fixation rate ν of new mutations is the product of the mutation rate μ per site, the chromosomal population size in mammalian species $2N$ and the probability of fixation

$$\nu_S = \mu \times 2N \times \frac{\mu 2s}{1-e^{-4Ns}} = \frac{\mu 4Ns}{1-e^{-4Ns}} \quad . \tag{11}$$

The rate of substitution of neutral mutations is

$$\nu_0 = \frac{\mu 2N}{2N} = \mu \quad . \tag{12}$$

We can now compare the relative rates of neutral mutations (Eq. 12) and selected mutations (Eq. 11). If we assume that all non-synonymous mutations at the same amino acid site have the same selection coefficient and that all synonymous mutations are neutral we have

$$\omega = \frac{\nu_s}{\nu_0} = \frac{4Ns}{1 - e^{-4Ns}} = \frac{2\gamma}{1 - e^{-2\gamma}} \quad , \tag{13}$$

where $\gamma = 2Ns$.

We have calculated $\omega = \frac{\sum dN}{\sum dS}$, where (dN, dS) was estimated for each of the 10,980 genes human-chimp-macaque trios using PAML [9]. To generate confidence intervals for dN/dS, we used 10,000 non-parametric bootstrap samples generated by sampling with replacement (dN, dS) pairs estimated from the 10,980 genes. Finally numerical estimates of $\gamma = 2Ns$ where obtained by using the “uniroot” function in R.

References

[1] Blanchette M, Kent WJ, Riemer C, Elnitski L, Smit AFA, et al. (2004) Aligning multiple genomic sequences with the threaded blockset aligner. Genome Res 14:708–715.

[2] Kuhn R, Karolchik D, Zweig A, Trumbower H, Thomas D, et al. (2006) The UCSC genome browser database: update 2007. Nucleic Acids Res .

[3] Kent WJ, Baertsch R, Hinrichs A, Miller W, Haussler D (2003) Evolution's cauldron: duplication, deletion, and rearrangement in the mouse and human genomes. Proc Natl Acad Sci USA 100:11484–11489.

[4] Pruitt KD, Tatusova T, Maglott DR (2005) NCBI Reference Sequence (RefSeq): a curated non-redundant sequence database of genomes, transcripts and proteins. Nucleic Acids Res 33:501–504.

[5] Hsu F, Kent WJ, Clawson H, Kuhn RM, Diekhans M, et al. (2006) The UCSC Known Genes. Bioinformatics 22:1036–1046.

[6] Ashurst JL, Chen CK, Gilbert JGR, Jekosch K, Keenan S, et al. (2005) The Vertebrate Genome Annotation (Vega) database. Nucleic Acids Res 33:459–465.

[7] Rhesus Macaque Genome Sequencing and Analysis Consortium (2007) Evolutionary and biomedical insights from the rhesus macaque genome. Science 13:222–234.

[8] Nielsen R, Yang Z (1998) Likelihood models for detecting positively selected amino acid sites and applications to the HIV-1 envelope gene. Genetics 148:929–936.

[9] Yang Z (1997) PAML: A program package for phylogenetic analysis by maximum likelihood. CABIOS 13:555–556.

[10] Zhang J, Nielsen R, Yang Z (2005) Evaluation of an improved branch-site likelihood method for detecting positive selection at the molecular level. Mol Biol Evol 22:2472–2479.

[11] Guindon S, Rodrigo AG, Dyer KA, Huelsenbeck JP (2004) Modeling the site-specific variation of selection patterns along lineages. Proc Natl Acad Sci U S A 101:12957–12962.

[12] Lunter G (2007) Dog as an outgroup to human and mouse. PLoS Comput Biol 3:e74.

[13] Yanai I, Benjamin H, Shmoish M, Chalifa-Caspi V, Shklar M, et al. (2005) Genome-wide midrange transcription profiles reveal expression level relationships in human tissue specification. Bioinformatics 21:650–659.

[14] Haygood R, Fedrigo O, Hanson B, Yokoyama KD, Wray GA (2007) Promoter regions of many neural- and nutrition-related genes have experienced positive selection during human evolution. Nat Genet 39:1140–1144.

[15] Drummond DA, Bloom JD, Adami C, Wilke CO, Arnold FH (2005) Why highly expressed proteins evolve slowly. Proc Natl Acad Sci U S A 102:14338–14343.

[16] Halpern AL, Bruno WJ (1998) Evolutionary distances for protein-coding sequences: modeling site-specific residue frequencies. Mol Biol Evol 15:910–917.

[17] Nielsen R, Yang Z (2003) Estimating the distribution of selection coefficients from phylogenetic data with applications to mitochondrial and viral DNA. Mol Biol Evol 20:1231–1239.

[18] Thorne JL, Choi SC, Yu J, Higgs PG, Kishino H (2007) Population genetics without intraspecific data. Mol Biol Evol 24:1667–1677. doi:10.1093/molbev/msm085.

[19] Kimura M, Ohta T (1969) The Average Number of Generations until Fixation of a Mutant Gene in a Finite Population. Genetics 61:763–771.

[20] Irizarry RA, Bolstad BM, Collin F, Cope LM, Hobbs B, et al. (2003) Summaries of Affymetrix GeneChip probe level data. Nucleic Acids Res 31:e15.

Table S1: Minimum species configurations required for likelihood ratio tests.

LRT	required species	ortholog groups
A: all branches	human, 2 other	16,529
B: primate branch	human, macaque, mouse or rat, dog	9,566
C: primate clade	human, chimp or macaque, 1 other	14,425
D: rodent branch	human, mouse, rat, dog	10,762
E: rodent clade	human, mouse, rat	8,991
F: human lineage	human, chimp, 1 other	14,558
G: chimp lineage	human, chimp, 1 other	14,558
H: hominid branch	human, chimp, macaque, 1 other	10,980
K: macaque lineage	human, macaque, mouse or rat or dog	12,499

Table S2: GO categories over-represented among predicted PSGs.

Category	Description	Gene number All	Gene number PSGs	Gene number E[PSGs]	Fold Enrich.	*P*-value MWU	*P*-value FET
	Biological process						
GO:0007606	sensory perception of chemical stimulus	255	24	6.2	3.9	$\mathbf{4.3 \times 10^{-39}}$	$\mathbf{1.5 \times 10^{-08}}$
GO:0006955	immune response	457	55	11.1	5.0	$\mathbf{1.4 \times 10^{-33}}$	$\mathbf{1.4 \times 10^{-22}}$
GO:0007608	sensory perception of smell	229	15	5.5	2.7	$\mathbf{6.1 \times 10^{-31}}$	4.8×10^{-04}
GO:0050896	response to stimulus	1887	101	45.7	2.2	$\mathbf{4.4 \times 10^{-26}}$	$\mathbf{1.1 \times 10^{-14}}$
GO:0002376	immune system process	608	59	14.7	4.0	$\mathbf{6.7 \times 10^{-25}}$	$\mathbf{1.7 \times 10^{-19}}$
GO:0006952	defense response	412	34	10.0	3.4	$\mathbf{1.3 \times 10^{-20}}$	$\mathbf{9.8 \times 10^{-10}}$
GO:0051707	response to other organism	138	13	3.3	3.9	$\mathbf{3.8 \times 10^{-15}}$	6.0×10^{-05}
GO:0007600	sensory perception	559	31	13.5	2.3	$\mathbf{1.1 \times 10^{-12}}$	$\mathbf{1.7 \times 10^{-05}}$
GO:0002526	acute inflammatory response	55	11	1.3	8.3	$\mathbf{4.7 \times 10^{-11}}$	$\mathbf{6.7 \times 10^{-08}}$
GO:0050909	sensory perception of taste	25	8	0.6	13.2	$\mathbf{1.4 \times 10^{-10}}$	$\mathbf{8.3 \times 10^{-08}}$
GO:0009611	response to wounding	321	23	7.8	3.0	$\mathbf{3.2 \times 10^{-10}}$	$\mathbf{6.9 \times 10^{-06}}$
GO:0051704	multi-organism process	222	17	5.4	3.2	$\mathbf{3.3 \times 10^{-10}}$	4.7×10^{-05}
GO:0006954	inflammatory response	233	20	5.6	3.5	$\mathbf{8.4 \times 10^{-10}}$	$\mathbf{1.6 \times 10^{-06}}$
GO:0002541	activation of plasma proteins during acute inflammatory response	28	7	0.7	10.3	$\mathbf{1.3 \times 10^{-09}}$	$\mathbf{3.5 \times 10^{-06}}$
GO:0006956	complement activation	28	7	0.7	10.3	$\mathbf{1.3 \times 10^{-09}}$	$\mathbf{3.5 \times 10^{-06}}$
GO:0009607	response to biotic stimulus	188	14	4.5	3.1	$\mathbf{1.7 \times 10^{-09}}$	3.2×10^{-04}
GO:0045087	innate immune response	70	8	1.7	4.7	$\mathbf{1.9 \times 10^{-09}}$	3.7×10^{-04}
GO:0009615	response to virus	69	7	1.7	4.2	$\mathbf{3.0 \times 10^{-08}}$	2.4×10^{-03}
GO:0009617	response to bacterium	67	6	1.6	3.7	$\mathbf{4.2 \times 10^{-08}}$	7.9×10^{-03}
GO:0002682	regulation of immune system process	60	11	1.5	7.6	$\mathbf{8.5 \times 10^{-08}}$	$\mathbf{1.0 \times 10^{-06}}$
GO:0016064	immunoglobulin mediated immune response	36	8	0.9	9.2	$\mathbf{1.1 \times 10^{-07}}$	$\mathbf{1.5 \times 10^{-05}}$
GO:0042742	defense response to bacterium	61	5	1.5	3.4	$\mathbf{1.2 \times 10^{-07}}$	2.2×10^{-02}
GO:0002253	activation of immune response	40	9	1.0	9.3	$\mathbf{1.2 \times 10^{-07}}$	$\mathbf{1.9 \times 10^{-06}}$
GO:0007186	G-protein coupled receptor protein signaling pathway	792	39	19.2	2.0	$\mathbf{1.4 \times 10^{-07}}$	2.5×10^{-05}
GO:0002252	immune effector process	69	11	1.7	6.6	$\mathbf{1.5 \times 10^{-07}}$	$\mathbf{3.0 \times 10^{-06}}$
GO:0006959	humoral immune response	56	9	1.4	6.6	$\mathbf{1.6 \times 10^{-07}}$	$\mathbf{7.2 \times 10^{-06}}$
GO:0002684	positive regulation of immune system process	49	9	1.2	7.6	$\mathbf{1.7 \times 10^{-07}}$	$\mathbf{8.4 \times 10^{-06}}$
GO:0050778	positive regulation of immune response	49	9	1.2	7.6	$\mathbf{1.7 \times 10^{-07}}$	$\mathbf{8.4 \times 10^{-06}}$
GO:0050776	regulation of immune response	59	11	1.4	7.7	$\mathbf{1.8 \times 10^{-07}}$	$\mathbf{1.0 \times 10^{-06}}$
GO:0002455	humoral immune response mediated by circulating immunoglobulin	24	6	0.6	10.3	$\mathbf{3.0 \times 10^{-07}}$	$\mathbf{1.8 \times 10^{-05}}$
GO:0019724	B cell mediated immunity	37	8	0.9	8.9	$\mathbf{3.2 \times 10^{-07}}$	$\mathbf{1.8 \times 10^{-05}}$
GO:0006968	cellular defense response	55	5	1.3	3.8	$\mathbf{3.5 \times 10^{-07}}$	1.1×10^{-02}
GO:0019882	antigen processing and presentation	27	4	0.7	6.1	$\mathbf{5.7 \times 10^{-07}}$	5.0×10^{-03}
GO:0006958	complement activation, classical pathway	23	6	0.6	10.8	$\mathbf{6.1 \times 10^{-07}}$	$\mathbf{1.4 \times 10^{-05}}$
GO:0050877	neurological process	811	34	19.6	1.7	$\mathbf{7.5 \times 10^{-07}}$	1.5×10^{-03}
GO:0006957	complement activation, alternative pathway	11	2	0.3	7.5	$\mathbf{1.5 \times 10^{-06}}$	2.8×10^{-02}
GO:0019835	cytolysis	15	3	0.4	8.3	$\mathbf{2.2 \times 10^{-06}}$	5.2×10^{-03}
GO:0051240	positive regulation of multicellular organismal process	61	10	1.5	6.8	$\mathbf{2.6 \times 10^{-06}}$	$\mathbf{1.1 \times 10^{-05}}$
GO:0002449	lymphocyte mediated immunity	53	10	1.3	7.8	$\mathbf{5.7 \times 10^{-06}}$	$\mathbf{2.5 \times 10^{-06}}$
GO:0009605	response to external stimulus	462	27	11.2	2.4	$\mathbf{5.8 \times 10^{-06}}$	4.2×10^{-05}
GO:0002460	adaptive immune response based on somatic recombination of immune receptors built from immunoglobulin superfamily domains	57	13	1.4	9.4	$\mathbf{8.0 \times 10^{-06}}$	$\mathbf{1.1 \times 10^{-08}}$
GO:0002250	adaptive immune response	58	13	1.4	9.3	$\mathbf{1.5 \times 10^{-05}}$	$\mathbf{1.1 \times 10^{-08}}$
GO:0007338	single fertilization	39	4	0.9	4.2	$\mathbf{1.7 \times 10^{-05}}$	1.4×10^{-02}
	Molecular function						
GO:0004984	olfactory receptor activity	229	15	5.5	2.7	$\mathbf{6.9 \times 10^{-36}}$	4.8×10^{-04}
GO:0001584	rhodopsin-like receptor activity	540	30	13.1	2.3	$\mathbf{8.4 \times 10^{-18}}$	2.2×10^{-05}
GO:0004930	G-protein coupled receptor activity	625	37	15.1	2.4	$\mathbf{2.5 \times 10^{-14}}$	$\mathbf{5.1 \times 10^{-07}}$
GO:0004888	(*) transmembrane receptor activity	972	55	23.5	2.3	$\mathbf{4.0 \times 10^{-12}}$	$\mathbf{3.3 \times 10^{-09}}$
GO:0004872	(*) receptor activity	1411	89	34.1	2.6	$\mathbf{2.5 \times 10^{-10}}$	$\mathbf{1.9 \times 10^{-17}}$
GO:0008527	taste receptor activity	14	5	0.3	14.8	$\mathbf{1.3 \times 10^{-08}}$	$\mathbf{1.4 \times 10^{-05}}$
GO:0008009	chemokine activity	34	5	0.8	6.1	$\mathbf{3.7 \times 10^{-07}}$	1.3×10^{-03}
GO:0042379	chemokine receptor binding	34	5	0.8	6.1	$\mathbf{3.7 \times 10^{-07}}$	1.3×10^{-03}
GO:0030414	protease inhibitor activity	110	6	2.7	2.3	$\mathbf{4.1 \times 10^{-07}}$	5.1×10^{-02}
GO:0004866	endopeptidase inhibitor activity	110	6	2.7	2.3	$\mathbf{4.1 \times 10^{-07}}$	5.1×10^{-02}

GO:0019965	interleukin binding	33	1	0.8	1.3	$\mathbf{2.2 \times 10^{-06}}$	5.5×10^{-01}
GO:0005125	cytokine activity	184	13	4.5	2.9	$\mathbf{7.8 \times 10^{-06}}$	5.8×10^{-04}
GO:0008173	RNA methyltransferase activity	19	1	0.5	2.2	$\mathbf{8.4 \times 10^{-06}}$	3.7×10^{-01}
GO:0004907	interleukin receptor activity	28	1	0.7	1.5	$\mathbf{2.1 \times 10^{-05}}$	5.0×10^{-01}
GO:0017171	serine hydrolase activity	150	9	3.6	2.5	$\mathbf{2.1 \times 10^{-05}}$	1.1×10^{-02}
	Cellular component						
GO:0005576	extracellular region	889	43	21.5	2.0	$\mathbf{2.6 \times 10^{-11}}$	4.5×10^{-05}
GO:0005615	extracellular space	354	19	8.6	2.2	$\mathbf{6.8 \times 10^{-08}}$	4.1×10^{-03}
GO:0042611	MHC protein complex	14	4	0.3	11.8	$\mathbf{2.8 \times 10^{-07}}$	2.8×10^{-04}
GO:0031224	(*) intrinsic to membrane	3817	168	92.4	1.8	$\mathbf{7.7 \times 10^{-06}}$	$\mathbf{1.4 \times 10^{-16}}$
GO:0042612	MHC class I protein complex	12	2	0.3	6.9	$\mathbf{8.9 \times 10^{-06}}$	3.3×10^{-02}
GO:0016021	(*) integral to membrane	3799	168	91.9	1.8	$\mathbf{8.9 \times 10^{-06}}$	$\mathbf{1.0 \times 10^{-16}}$

Bold indicates FWER $<$0.05 (Holm correction).

Table S3: PANTHER categories over-represented among genes predicted to be under positive selection.

Category	Description	Gene number All	PSGs	E[PSGs]	Fold Enrich.	*P*-value MWU	*P*-value FET
	Biological process						
BP00148	Immunity and defense	968	76	23.4	3.2	$\mathbf{2.2 \times 10^{-25}}$	$\mathbf{3.0 \times 10^{-20}}$
BP00184	Olfaction	122	8	3.0	2.7	$\mathbf{8.6 \times 10^{-15}}$	9.6×10^{-03}
BP00183	Chemosensory perception	128	8	3.1	2.6	$\mathbf{1.3 \times 10^{-13}}$	1.3×10^{-02}
BP00155	Macrophage-mediated immunity	97	10	2.3	4.3	$\mathbf{4.6 \times 10^{-09}}$	$\mathbf{1.2 \times 10^{-04}}$
BP00149	T-cell mediated immunity	134	15	3.2	4.6	$\mathbf{1.2 \times 10^{-08}}$	$\mathbf{8.7 \times 10^{-07}}$
BP00157	Natural killer cell mediated immunity	35	7	0.8	8.3	$\mathbf{1.6 \times 10^{-08}}$	$\mathbf{1.7 \times 10^{-05}}$
BP00107	Cytokine and chemokine mediated signaling pathway	192	10	4.6	2.2	$\mathbf{4.1 \times 10^{-08}}$	1.9×10^{-02}
BP00255	Cytokine/chemokine mediated immunity	88	7	2.1	3.3	$\mathbf{7.6 \times 10^{-08}}$	5.5×10^{-03}
BP00176	Blood clotting	69	4	1.7	2.4	$\mathbf{2.2 \times 10^{-07}}$	8.6×10^{-02}
BP00152	B-cell- and antibody-mediated immunity	73	16	1.8	9.1	$\mathbf{4.8 \times 10^{-07}}$	$\mathbf{1.5 \times 10^{-11}}$
BP00156	Interferon-mediated immunity	56	7	1.4	5.2	$\mathbf{8.0 \times 10^{-07}}$	3.8×10^{-04}
BP00153	Complement-mediated immunity	40	7	1.0	7.2	$\mathbf{6.0 \times 10^{-06}}$	$\mathbf{4.3 \times 10^{-05}}$
BP00150	MHCI-mediated immunity	12	2	0.3	6.9	$\mathbf{1.6 \times 10^{-05}}$	3.3×10^{-02}
BP00151	MHCII-mediated immunity	12	3	0.3	10.3	$\mathbf{3.4 \times 10^{-05}}$	2.6×10^{-03}
BP00288	Granulocyte-mediated immunity	47	7	1.1	6.2	$\mathbf{3.4 \times 10^{-05}}$	1.3×10^{-04}
BP00240	Fertilization	26	4	0.6	6.4	$\mathbf{6.2 \times 10^{-05}}$	3.3×10^{-03}
BP00299	Steroid hormone metabolism	21	3	0.5	5.9	$\mathbf{8.3 \times 10^{-05}}$	1.4×10^{-02}
	Molecular function						
MF00173	Defense/immunity protein	223	46	5.4	8.5	$\mathbf{1.4 \times 10^{-35}}$	$\mathbf{9.7 \times 10^{-30}}$
MF00004	Immunoglobulin receptor family member	66	27	1.6	16.9	$\mathbf{4.4 \times 10^{-19}}$	$\mathbf{1.0 \times 10^{-26}}$
MF00224	KRAB box transcription factor	409	9	9.9	0.9	$\mathbf{4.2 \times 10^{-13}}$	6.6×10^{-01}
MF00018	Chemokine	29	4	0.7	5.7	$\mathbf{2.0 \times 10^{-08}}$	5.0×10^{-03}
MF00102	Protease inhibitor	97	3	2.3	1.3	$\mathbf{1.1 \times 10^{-07}}$	4.2×10^{-01}
MF00216	Serine protease	140	10	3.4	3.0	$\mathbf{1.6 \times 10^{-06}}$	2.2×10^{-03}
MF00250	Serine protease inhibitor	63	2	1.5	1.3	$\mathbf{7.5 \times 10^{-06}}$	4.5×10^{-01}
MF00175	Major histocompatibility complex antigen	15	4	0.4	11.0	$\mathbf{1.1 \times 10^{-05}}$	3.7×10^{-04}
MF00177	Other defense and immunity protein	50	4	1.2	3.3	$\mathbf{1.7 \times 10^{-05}}$	3.2×10^{-02}
MF00174	Complement component	35	6	0.8	7.1	$\mathbf{3.6 \times 10^{-05}}$	1.7×10^{-04}
MF00176	Antibacterial response protein	26	1	0.6	1.6	$\mathbf{5.8 \times 10^{-05}}$	4.7×10^{-01}

Bold indicates FWER <0.05 (Holm correction).

Table S4: Numbers of genes under selection by various switching models

branch/ clade	lineage[a]	type[b]	model 1p[c]	2p[d]	full[e]
branch	human	all	244.0	243.5	207.9
		only	2.6	2.6	1.5
	chimpanzee	all	252.3	251.7	233.5
		only	2.9	2.9	1.9
	hominid	all	280.4	280.2	283.3
		only	3.0	3.1	1.5
	macaque	all	313.9	313.4	340.9
		only	12.6	12.7	7.9
	primate	all	320.1	319.8	360.5
		only	6.4	6.4	6.7
	mouse	all	304.9	304.3	290.4
		only	6.6	6.6	4.1
	rat	all	294.3	293.9	253.9
		only	2.2	2.2	1.1
	rodent	all	339.7	339.2	393.9
		only	21.7	21.8	36.0
	dog	all	295.0	294.5	308.4
		only	4.9	4.9	0.3
clade	primate	all	132.7	132.2	140.2
		only	8.2	8.2	6.7
	rodent	all	239.8	239.2	242.4
		only	14.5	14.5	13.9
	all	–	49.6	49.4	46.6

[a]Branch or clade of interest. *Hominid* is branch leading to human and chimpanzee, *primate* is branch leading to hominid and macaque, and *rodent* is branch leading to mouse and rat (see Fig. 4).

[b]Whether number represents sum over all histories in which branch or clade of interest is under selection or the single history in which only that branch (or only the branches in that clade) is under selection.

[c]One-parameter model (equal gain and loss rates shared across all branches).

[d]Two-parameter model (separate gain and loss rates shared across all branches).

[e]Full model (separate gain and loss rates for all branches).

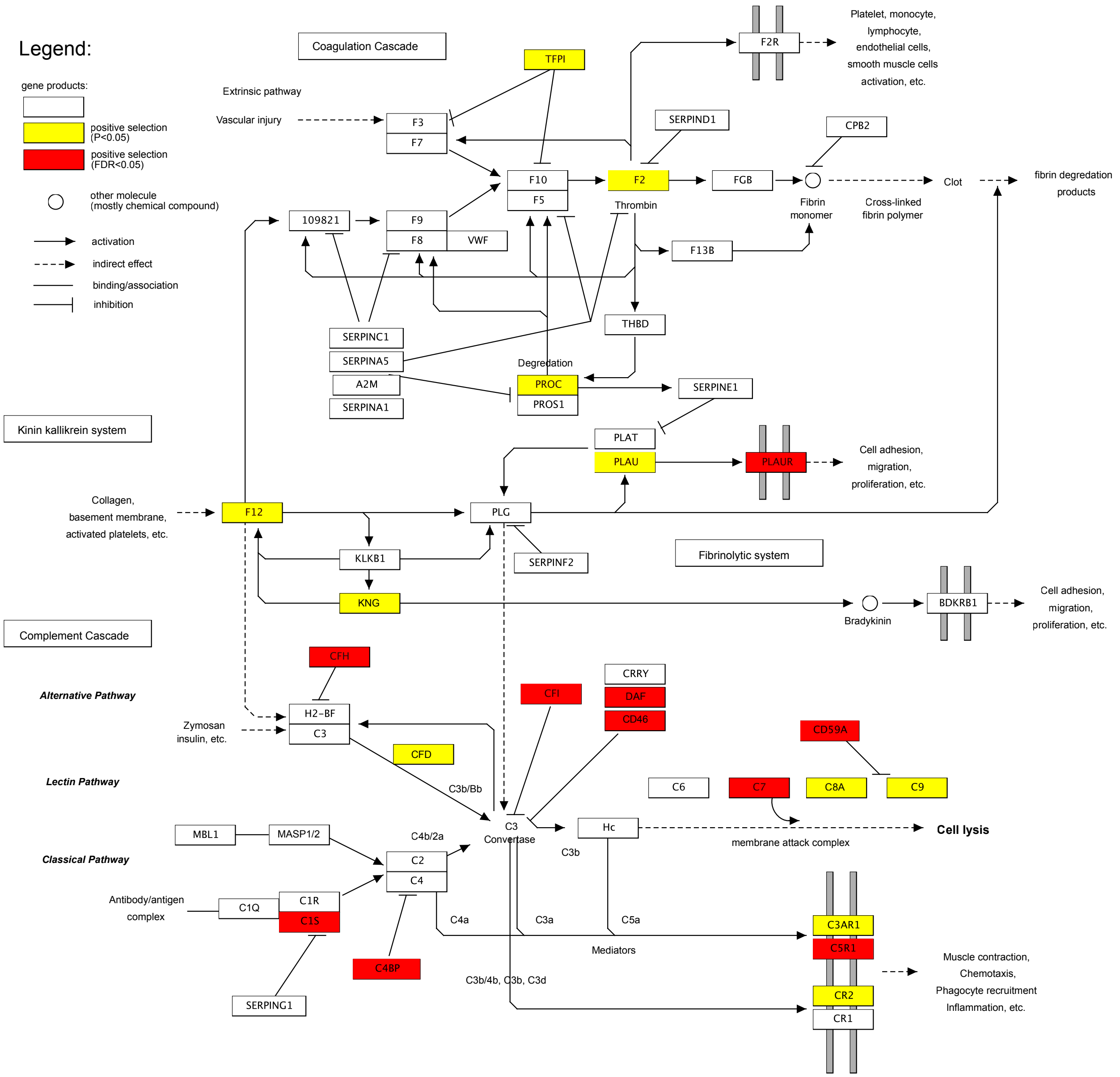

Figure S1: Complement component and coagulation pathways (as represented in the KEGG database) with PSGs highlighted.

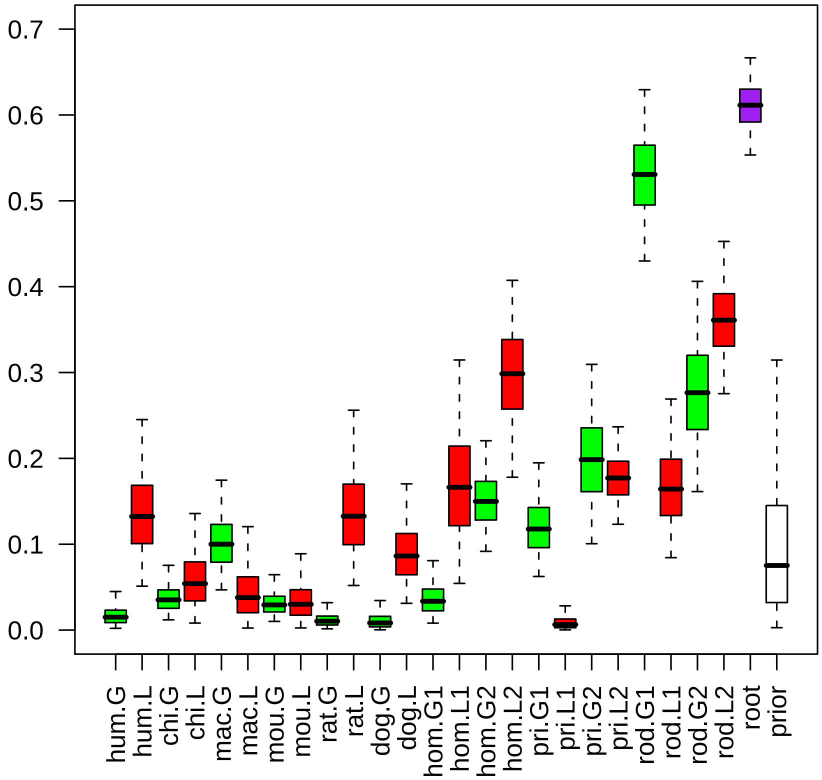

Figure S2: Boxplot showing marginal posterior distributions for elements of $\boldsymbol{\theta}$, as inferred by the Gibbs sampling algorithm. The y-axis indicates the parameter values and the different parameters are shown along the x-axis. Branches are those leading to human (*hum*), chimpanzee (*chi*), macaque (*mac*), mouse (*mou*), rat (*rat*), dog (*dog*), the hominid ancestor (*hom*), the primate ancestor (*pri*), and the rodent ancestor (*rod*). Gain parameters are indicated by ".G" and shown in green, and loss parameters are indicated by ".L" and shown in red. Two sets of parameters are shown for ancestral branches, corresponding to (1) early and (2) late events. The posterior densities for the parameter defining the probability of selection at the root of the tree (θ_0) is also indicated ("root"), as is the prior density assumed for all parameters (Beta(1, 9)). As in a standard boxplot, the box boundaries indicate the lower and upper quartiles (0.25 and 0.75 quantiles) and the horizontal bar indicates the median, but here the whiskers indicate 95% credible intervals (i.e., 0.025 and 0.975 quantiles) rather than the extreme values of all samples. The number of samples was 9900.

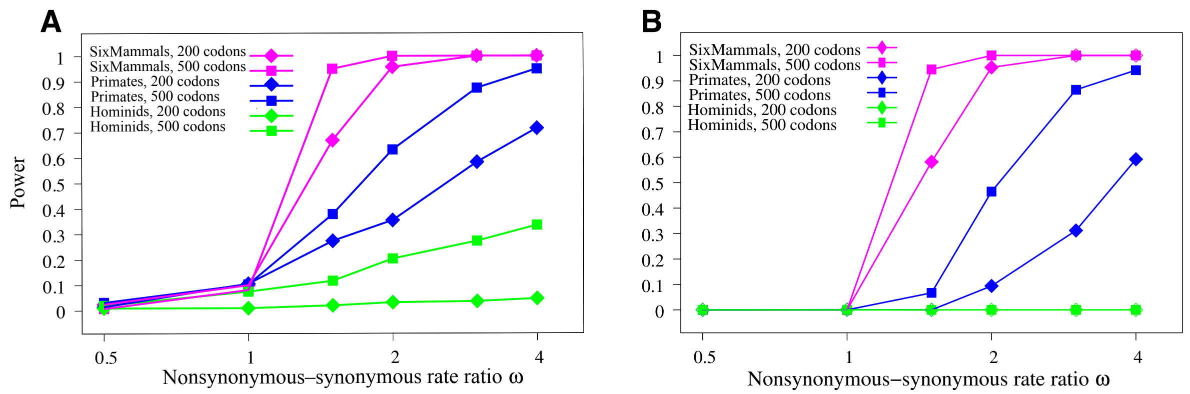

Figure S3: Power of the LRT for selection on any branch of the phylogeny as a function of the nonsynonymous-synonymous rate ratio ω. Power is defined as the fraction of tests resulting in (A) nominal $P < 0.05$ or (B) nominal P less than the threshold required to ensure FDR < 0.05 for the real data set. Simulations were conducted as described in Fig. 6.

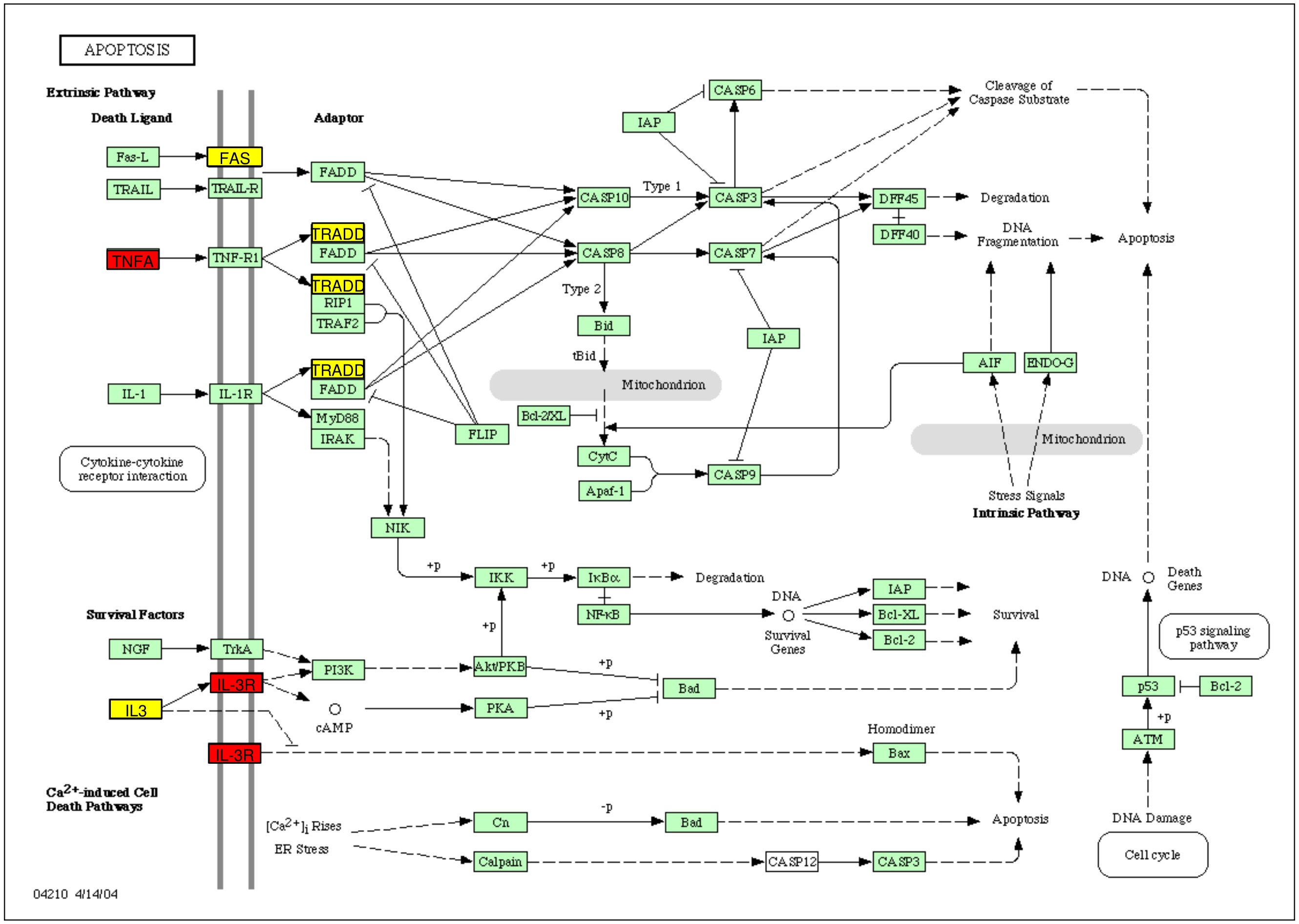

Figure S4: Apoptosis pathway from KEGG with PSGs highlighted. Legend is as in Fig. S1.

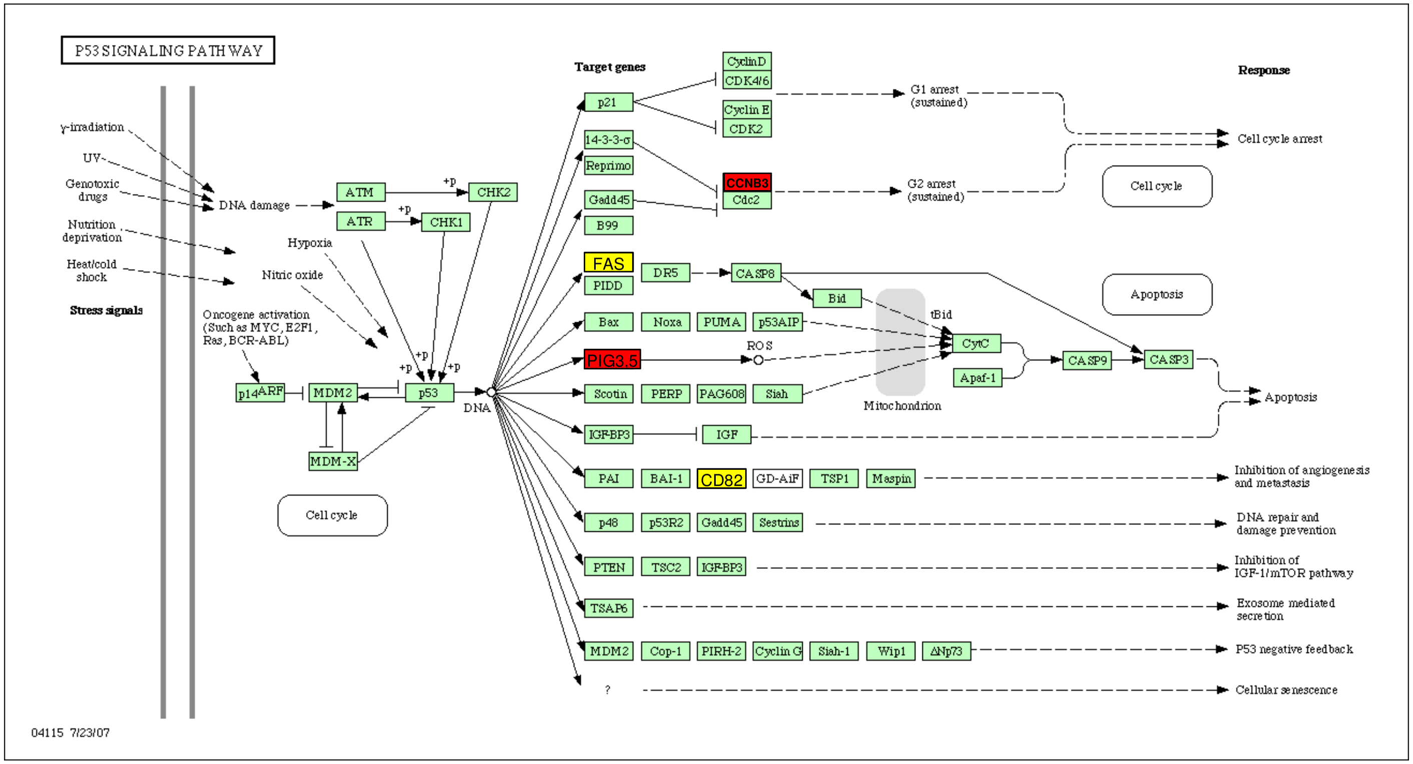

Figure S5: p53 signaling pathway from KEGG with PSGs highlighted. Legend is as in Fig. S1.

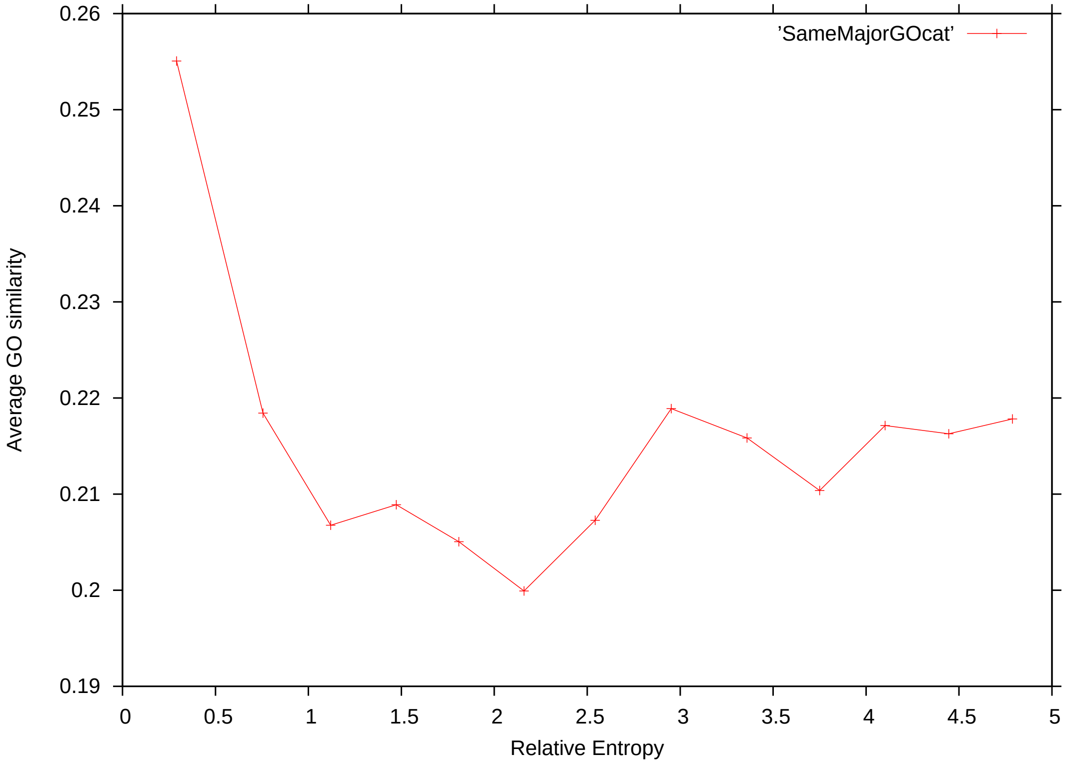

Figure S6: A measure of GO similarity measure vs. the relative entropy calculated from the posterior distributions of the selection histories of gene pairs. The measure of GO similarity for a gene pair i and j is defined as $s_{ij} = \frac{|G_i \cap G_j|}{|G_i \cup G_j|}$, where G_x is the set of all GO categories of gene x. Gene pairs were binned according to relative entropy and an average GO similarity was calculated for each bin. Only pairs assigned to the same major category (i.e., biological process, molecular function and cellular component) were compared. Gene pairs with low relative entropy tend on average to have higher GO similarity scores, suggesting that genes with similar selection histories carry similar functions and that some degree of co-evolution is frequent.

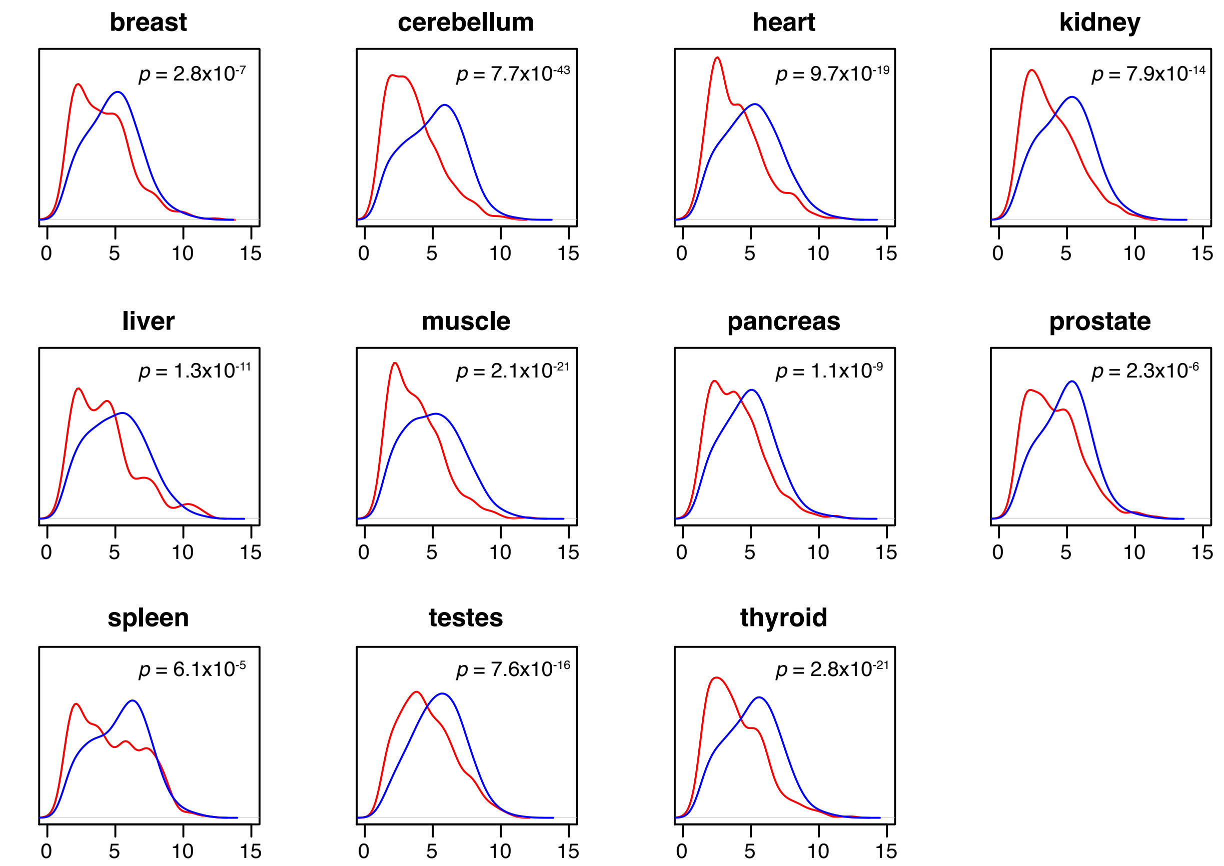

Figure S7: Distributions of expression levels in PSGs (red) and non-PSGs (blue) for all eleven tissue types, as estimated from Affymetrix Human Exon 1.0 ST Array data by the RMA algorithm [20]. One-sided MWU P-values are shown, indicating significance of leftward shift of PSG distribution.

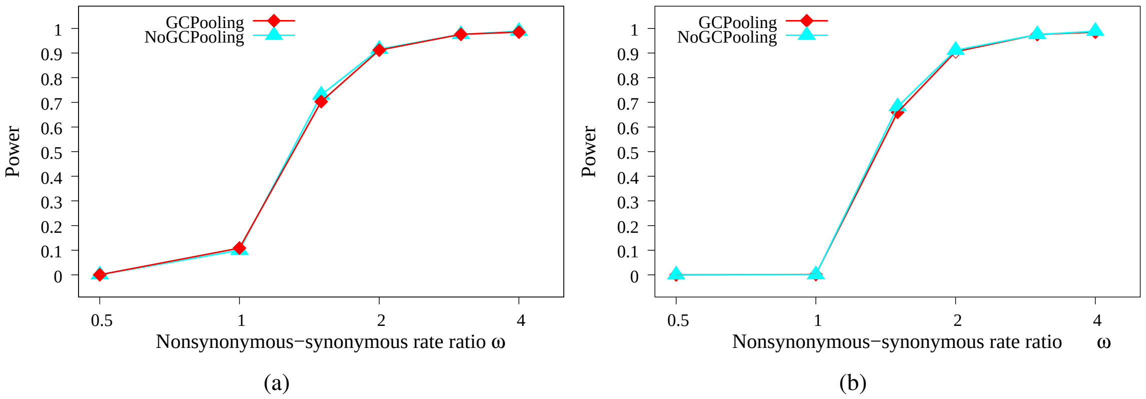

Figure S8: Fraction of genes predicted to be under positive selection by the all-branch LRT, shown as a function of the nonsynonymous-synonymous rate ratio ω. Plotted are the fractions of tests resulting in (a) nominal $P < 0.05$ or (b) nominal P less than the threshold required to ensure FDR < 0.05 for the real data set. These fractions represent sensitivity or power for $\omega > 1$, and false positive rates for $\omega \leq 1$. Separate curves are shown for the cases of G+C pooling and full estimation of all parameters per gene.

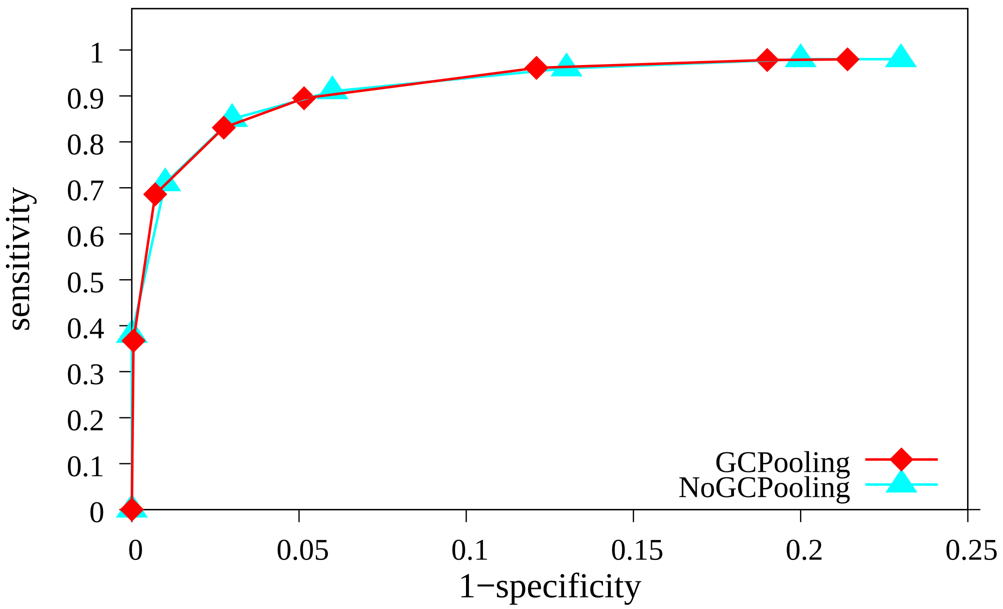

Figure S9: Receiver-operating characteristic (ROC) curve showing the trade-off between sensitivity and specificity for the all-branch LRT as the P-value threshold is varied. Sensitivity is defined as the fraction of synthetic data sets with $\omega = 1.5$ predicted to be under positive selection, and 1–specificity is the fraction of data sets with $\omega = 0.5$ predicted to be under positive seletion. Separate curves are shown for the cases of G+C pooling and full estimation of all parameters per gene.

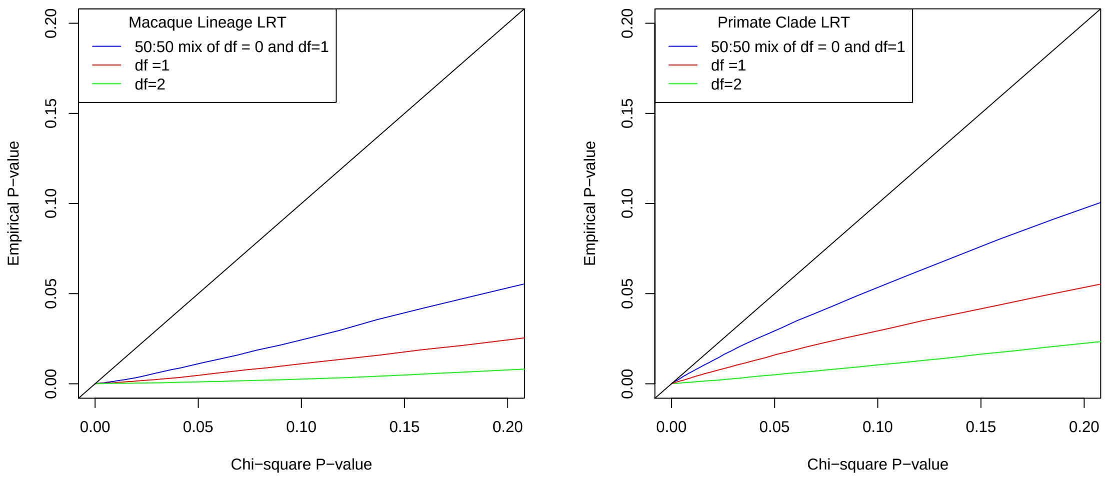

(a) (b)

Figure S10: Comparison of empirical and χ^2-based P-values for the LRTs for (a) the macaque lineage and (b) the primate clade. Shown are P-values based on a 50:50 mixture of a $\chi^2_{df=1}$ distribution and a point mass at zero, a $\chi^2_{df=1}$ distribution, and a $\chi^2_{df=2}$ distribution. The χ^2-based P-values can be seen to be conservative.

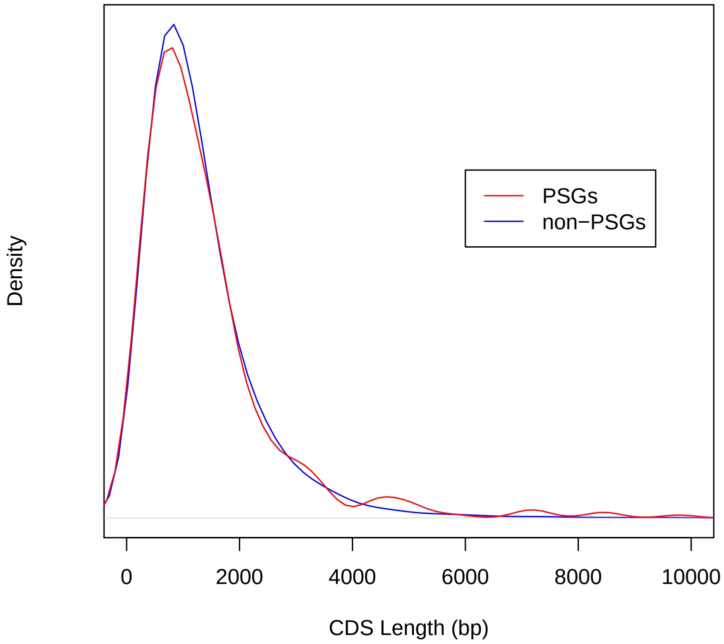

Figure S11: Distributions of coding sequence (CDS) lengths for positively selected genes (PSGs) and non-positively selected genes (non-PSGs). The PSG length distribution has a slightly heavier tail, resulting in a somewhat higher mean (1426.1 bp [sd 1393.5] vs. 1296.5 bp [sd 1018.4] for non-PSGs, but the medians are similar (1063 bp for PSGs, 1050 bp for non-PSGs) and the PSG distribution does not show a significant rightward shift according to a one-sided Mann-Whitney U test ($P = 0.33$). Plotted are kernel density estimates based on a Gaussian kernel with a smoothing bandwidth standard deviation of 300 bp ('density' function in R).

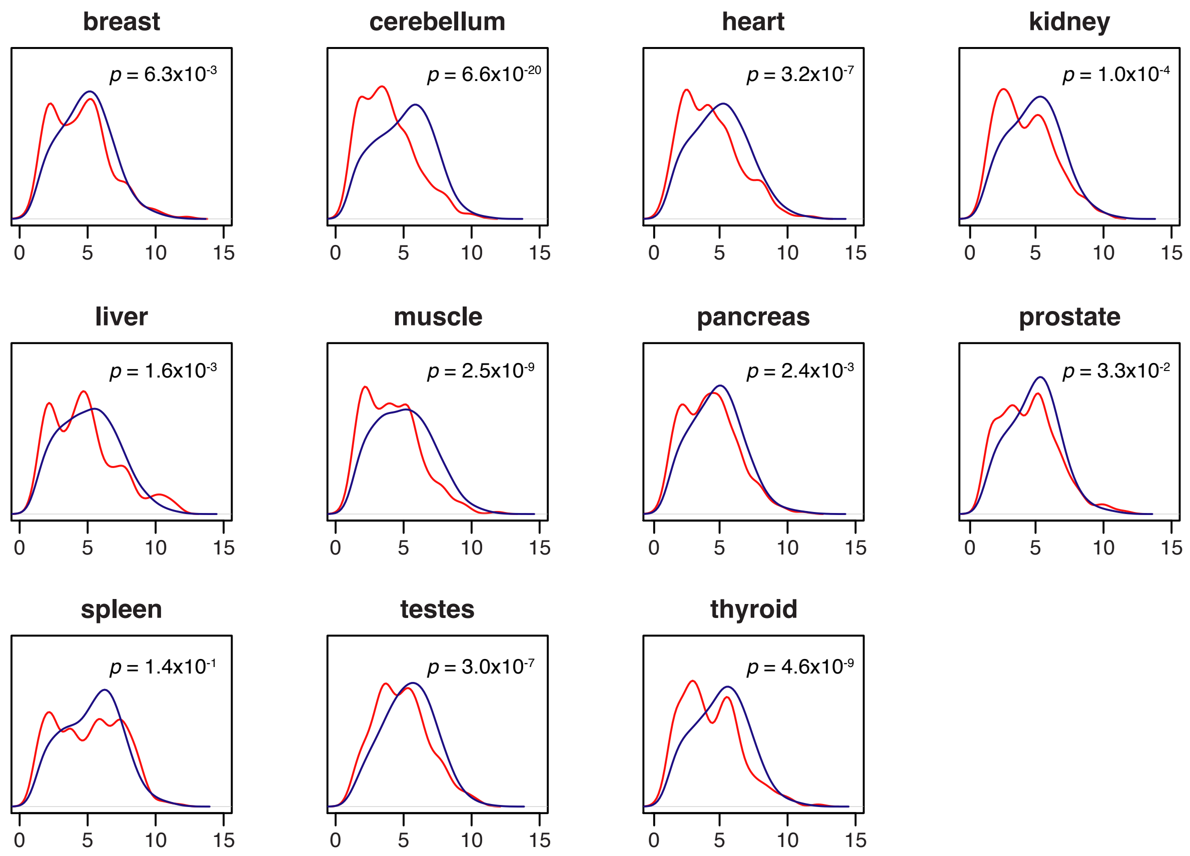

Figure S12: Distributions of expression levels in PSGs (red) and non-PSGs (blue), where PSGs are conservatively defined by an FDR threshold of 0.01 for genes with low expression and an FDR threshold of 0.10 for genes with high expression (see Text S1).

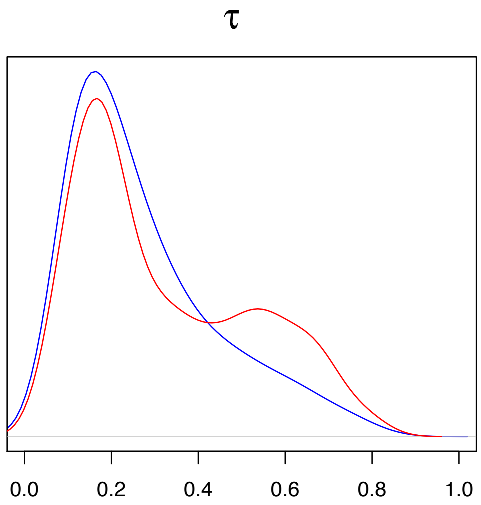

Figure S13: Distribution of degree of tissue bias in expression (τ) in PSGs (red) and non-PSGs (blue), where PSGs are conservatively defined by an FDR threshold of 0.01 for genes with high tissue bias and an FDR threshold of 0.10 for genes with low tissue bias (see Text S1). The rightward shift of the PSG distribution remains significant ($P = 1.8 \times 10^{-4}$, one-sided MWU test).
